# Black-Tailed Prairie Dogs, Cattle, and the Conservation of North America’s Arid Grasslands

**DOI:** 10.1371/journal.pone.0118602

**Published:** 2015-03-11

**Authors:** Rodrigo Sierra–Corona, Ana Davidson, Ed L. Fredrickson, Hugo Luna-Soria, Humberto Suzan-Azpiri, Eduardo Ponce-Guevara, Gerardo Ceballos

**Affiliations:** 1 Instituto de Ecología, Universidad Nacional Autónoma de México, Mexico DF, México; 2 Department of Ecology and Evolution, Stony Brook University, Stony Brook, New York, United States of America; 3 Department of Agriculture, Eastern Kentucky University, Richmond, Kentucky, United States of America; 4 Facultad de Ciencias Naturales, Universidad Autónoma de Querétaro, Querétaro, México; University of Brasilia, BRAZIL

## Abstract

Prairie dogs (*Cynomys* spp.) have been eliminated from over 95% of their historic range in large part from direct eradication campaigns to reduce their purported competition with cattle for forage. Despite the longstanding importance of this issue to grassland management and conservation, the ecological interactions between cattle and prairie dogs have not been well examined. We address this issue through two complementary experiments to determine if cattle and prairie dogs form a mutualistic grazing association similar to that between prairie dogs and American bison. Our experimental results show that cattle preferentially graze along prairie dog colony edges and use their colony centers for resting, resembling the mutualistic relationship prairie dogs have with American bison. Our results also show that prairie dog colonies are not only an important component of the grassland mosaic for maintaining biodiversity, but also provide benefits to cattle, thereby challenging the long-standing view of prairie dogs as an undesirable pest species in grasslands.

## Introduction

Grasslands cover approximately 55 million km^2^ (43%) of the terrestrial Earth surface, providing livelihoods for nearly 800 million people, being the most important ecosystem for the provision of the global food supply, and are considered biodiversity hotspots [1]. Yet, grasslands have been subject to intense human pressure due to increasing demand for the expansion of agricultural lands, urbanization, water extraction, and mineral exploitation, causing declines in grassland ecological health and economic productivity [2,3]. Climate change is also important, causing increases in the duration and frequency of droughts, and exacerbating widespread invasion of shrubs into grasslands [4]. In North America, grasslands have dramatically declined; tallgrass, mixed-grass, and shortgrass prairie cover only 1%, 20%, and 30%, of their historical extent, respectively, with the remaining portions being highly fragmented [5,6].

Grasslands are fundamentally shaped by two key functional groups that have co-evolved for thousands of years, namely, large migratory mammalian herbivores and small to medium-sized burrowing herbivorous mammals [7,8]. Both functional groups of herbivores play keystone and/or ecosystem engineering roles and have complementary and interactive effects on grassland ecosystem structure and function [9–12].

In North America, American bison (*Bison bison*), through their grazing and wallowing, create grazing lawns and increase grassland biodiversity, prevent encroachment of shrubs through trampling and consumption of woody vegetation, and increase nutrient availability through the deposition of dung and urine [13]. Grazing by bison impacts plant survival, stimulates plant nitrogen uptake and aboveground production, and alters grassland community structure and ecosystem processes [14,15].

Prairie dogs also increase habitat heterogeneity and biodiversity of grassland ecosystems by creating islands of unique habitat [12]. Like many other burrowing mammals, prairie dogs are highly social, and aggregate into large colonies where they transform the landscape through their burrowing and foraging activities [16,17]. They are prey of a wide variety to predators and their borrows provide refuge for numerous animals [18]. Prairie dog grazing increases forage quality by reducing leaf age and enhancing plant nitrogen uptake, attracting large herbivores to their colonies [19,20]. The center of the prairie dog colonies, where most borrow-mounds appear, is dominated by bare ground and a low mat of heavily-grazed forbs and a mix of perennial and annual grasses [21]. In contrast, the edges of the colonies, which experience less impact by prairie dogs, are characterized by fewer burrows, taller vegetation, and moderately grazed annual and perennial grasses [21]. Prairie dogs also maintain the presence of grasslands and prevent their succession into shrubland by clipping shrubs and consuming their seedlings [12,22].

Bison and prairie dogs have co-evolved for thousands of years and constitute a grazing association, whereby bison preferentially graze along the edges of prairie dog colonies because of the availability of high quality forage; they also tend to rest within the center of colonies [19,23]. Bison that graze within prairie dog colonies have been shown to gain more weight compared to those that feed in off-colony grasslands [24]. Likewise, bison benefit prairie dogs by increasing nutrient quality of vegetation through their grazing and deposition of dung and urine [19,23,25], and their grazing lowers vegetation height, improving the ability of prairie dogs to detect predators [26].

Black-tailed prairie dogs (*Cynomys ludovicianus*) historically ranged across 40 million hectares of North America’s central grasslands. But, their populations have declined by more than 98% primarily as a result of habitat loss to agriculture, introduction of plague from Eurasia, and eradication campaigns designed to eliminate their purported competition with cattle for grazing resources [8,27,28]. In addition to the expenditure of millions of public tax dollars on eradication efforts to support private industry, the loss of prairie dogs has had a dramatic consequences on the ecological integrity of North America’s grassland ecosystem. The decline in prairie dogs is largely responsible for the near extinction of the black-footed ferret (*Mustela nigripens*), declines in other prairie dog-dependent species, encroachment of mesquite (*Prosopis glandulosa*) and other woody shrubs dispersed by cattle, and reduction in the economic productivity of this ecosystem [27–30]. Despite research showing that management strategies utilizing large-scale, lethal-control are neither scientifically justified nor cost effective, these “control” programs are still employed today and remain funded by taxpayers [7,12,31,32].

Domestic cattle (*Bos taurus*) have largely supplanted native American bison which were near-exterminated during the 19th century [13,33]. Bison and cattle are ecologically similar, [13] and while overgrazing by cattle has caused widespread desertification because of poor rangeland management [28,34], the activities of domestic cattle may partially substitute the functional role of the American bison [13,35,36]. In fact, cattle and prairie dogs seem to have a grazing association similar to that of bison and prairie dogs, with important interactive impacts on grasslands [8,27,37,38]. Cattle are frequent visitors to black-tailed prairie dog colonies [39,40], and in some areas where conservative grazing management schemes are employed, prairie dog populations have increased up to two-fold [8].

The Janos Biosphere Reserve (JBR), located in northwest Chihuahua, Mexico maintains one of the largest remnants of desert grassland in northwestern Mexico, and one of the largest remaining prairie dog colony complexes in North America [28,41]. Like most of the semi-arid grassland ecosystems in North America, the Janos grasslands have been transformed by the synergistic effects of chronic overgrazing due to poor cattle management, drought and climate change, and the expansion of industrial agriculture [28,41]. These changes in the Janos region have resulted in a 75% decline of the once 55,000 ha prairie dog complex, and widspread expansion of shrublands into native grasslands [28,41].

Despite the longstanding importance of effective and conservation-based management of grasslands, the ecological interactions between cattle and prairie dogs remains little studied [12]. Understanding and applying the ecological principles of the interactions between cattle and prairie dog is fundamental to informing sustainable grassland management policies and procedures. To understand this interaction and the impact on grassland health, we studied the ecological relationships between cattle and prairie dogs in the desert grasslands of the Janos Biosphere Reserve, Chihuahua, Mexico.

Our objective was to determine if cattle and prairie dogs form grazing associations similar to those described between prairie dogs and American bison [23,26]. We specifically addressed the following questions: i) Do cattle have habitat preferences for prairie dog colonies or other habitat types? ii) Do cattle show temporal variability in habitat selection, as bison do? iii) Do cattle selectively graze in particular areas of prairie dog colonies, such as colony edges? and iv) How can the results from this research be applied to conservation strategies for semi-arid grasslands?

## Materials and Methods

### Study site

We conducted our research within a 1,700 ha fenced area (30° 52’ 58.13” N, 108° 27’ 21.64” W) located in the “El Uno” Ecological Reserve within the Janos Biosphere Reserve, Chihuahua, northwestern Mexico [41]. Native annual grasses dominate the plant community, while native perennial grasses are sparse [8]. The mean annual precipitation in the region is 317 mm, and most of it occurs during the summer monsoon period. The mean annual temperature is 16.95°C [42].

### Experimental design

We conducted two complementary experiments to understand the relationship between habitat selection by cattle and the presence of black-tailed prairie dog colonies: 1) a Large-scale cattle habitat preference experiment and 2) a Small-scale cattle grazing preference experiment (descriptions provided below). We repeated all the design elements of each experiment three times from 2006–2007 in order to capture cattle grazing behavior under different periods of plant productivity: 1) low forage availability during the summer dry season (June-July) in 2006; 2) high forage availability at the end of the growing season in the fall (September-October) in 2006; and 3) the grass dormancy season during the winter (December-January) in 2006/2007. Both experiments were conducted using the recommended conservative grazing conditions (<40% use of available forage) [43], with the suggested stocking rate for this region being roughly 30–60 hectare by animal unit considering a 50% available forage consumption [44,45], because our goal, here, was to understand the ecological relationships between domestic cattle and black-tailed prairie dogs under conservative grazing.

Transportation and handling of domestic cattle in both experiments was according to the Norma Oficial Mexicana / Oficial Mexican Norm NOM-051-ZOO-1995 (Trato humanitario en la movilizacion de animales / Humanitarian treatment in animal mobilization) [46]. We followed low stress handling techniques performed by trained cattle technicians, in order to avoid stress and ensure humane treatment of all animals used in our study. No official permit was necessary to perform this experiment because pasture raised domestic cattle are not considered an experimental animal species under the Norma Oficial Mexicana NOM-062-ZOO-1999 (Especificaciones tecnicas para la produccion, cuidado y uso de los animales de laboratorio / Technical specifications for production, care and use of lab animals) [47]. All the facilities used in both experiments (trailer, paddocks, GPS collars and electric fences) were commercial equipment designed for cattle management. For the use of GPS collars we followed the American Society of Mastozoology recommendations [48]. Additionally, the animals were routinely observed and checked by a trained veterinarian. All the permissions and permits required for fieldwork and utilization of domestic cattle were requested and authorized by the cattle owners and the administration of The Nature Conservancy’s “El Uno” Ecological Reserve.

### Large-scale cattle habitat preference experiment

The first experiment assessed cattle habitat preference (vegetation types) by activity type (grazing, resting and walking) within a 1,700 ha pasture. We followed the movements of 36 cows (*Bos taurus*), randomly separated into three replicated groups of 12 individuals, to evaluate cattle habitat selection. Each cow was fitted with a Global Position System (GPS) collar equipped with movement sensors (Lotek, 2200LR) programmed to record spatial locations every 5-minutes. Cows were allowed to graze freely and move throughout the pasture for 6 consecutive days. Only the 12 cows fitted with the GPS collars where present within 1,700 ha pasture at the time, which represents 141 ha by animal unit. To record cattle activities, we used movement sensor data together and cattle movement patterns (see Peinetti *et al*. 2011 for details in the Peinetti s) [49].

**Fig 1:**
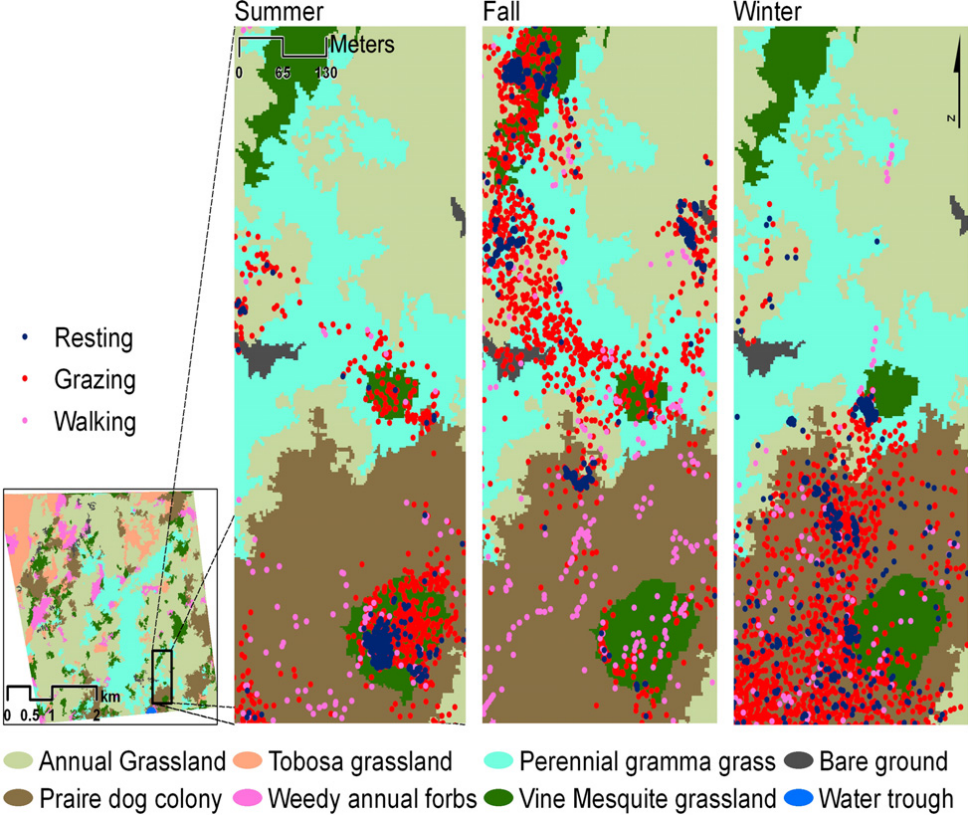
Spatial pattern of the three classes of cattle activity relative to the vegetation/ground cover types through seasons. Colored dots indicate cattle locations by activity across all weeks during each sample season.

We used a QuickBird satellite image with 0.6 m resolution object-oriented classification (see Laliberte *et al*. 2007) [50] to develop a vegetation map, enabling us to assess habitats with which cattle most associated. We identified six vegetation types: 1) Annual grassland [which covered 50% of the pasture and was dominated by six weeks three awn (*Aristida adscencionis*)]; 2) perennial gramma grassland [14% cover, dominated by perennial blue grama (*Bouteloua gracilis*.)]; 3) tobosa grassland [10% cover, dominated by tobosa, *Hilaria mutica*]; 4) vine mesquite grassland [8% cover, dominated by vine mesquite, *Panicum obtusum*]; 5) weedy annual forbs [5% cover; dominated by Russian thistle, *Salsola kali* & Palmer’s amaranth *(Amaranthus palmeri*]; and 6) prairie dog colonies [12% cover] (Fig. 1).

**Data analysis.** We performed a multivariate analysis of variance (MANOVA) with Repeated Measurements Analysis of Variance, to test the hypothesis of no difference in cattle habitat selection (location x vegetation) by activity and season, to test the null hypothesis of there being no variation in cattle habitat selection (records x vegetation) by activity and season. When the MANOVA tests where significant, we used general linear models (GLM) to identify the source of variation. There were no significant differences among replicates (i.e., 12 cow group) within seasons, so we performed complementary Chi-Square Goodness-of-Fit Test by season (all season locations x vegetation) to test the null hypothesis of random habitat use by activity (activity x vegetation). To determine the type of vegetation cattle preferred by activity by season, within the pasture, we used Bonferroni confidence intervals, calculating the percent cattle used each habitat type. When the percentage of availability of any given vegetation type was below the confidence interval, we considered it to have been significantly selected (P <0.001). When availability was above the confidence interval, that particular habitat was significantly avoided.

### Small-scale cattle grazing preference experiment

We conducted a second experiment to determine if cattle preferentially forage on a particular section of prairie dog colonies (i.e., grazing zones): the colony center, along colony edges, or off colonies. To identify the grazing patterns, we established in three independent prairie dog colonies, three blocks containing three 60 m X 60 m experimental plots. A cattle-grade electric fence enclosed each plot and encompassed the three grazing zones. To determine if cattle grazing location preference changes with the proportion of colony occupancy of the landscape, we varied the proportion of the grazing zones across the three plots on each colony: plot 1 consisted of 25% prairie dog colony and 75% off-colony grassland; plot 2 consisted of 50% prairie dog colony and 50% off-colony grassland; and plot 3 consisted of 75% prairie dog colony and 25% off-colony grassland. We calculated the prairie dog colony edge area for each plot by multiplying the average edge width by its length, using ArcMap 9.0 (ESRI). Four cows grazed each plot for 4 hours with a stocking rate of 65 ha by animal unit, once each season during the early morning grazing activity peak [51]. We recorded the type of cattle activity (grazing, resting and walking) observed for each cow within each plot every three minutes from a portable observation tower located 30 m from the edge of each plot.

**Data analysis** We performed a multivariate analysis of variance (MANOVA) with a Repeated Measurements Analysis approach to test the null hypothesis of cattle grazing zone selection by season. When MANOVA tests where significant, we performed a Chi-Square-Goodness-of-Fit to make comparisons of the cattle grazing zones (grazing x zone) by season. We also used Bonferroni confidence intervals to determine the preferred grazing zone; only grazing records where used in this analysis.

All data were assessed for normality, and if needed, normalized by log transformations using JMP version 8.

## Results

### Large-scale cattle habitat preference experiment

**Fig 2:**
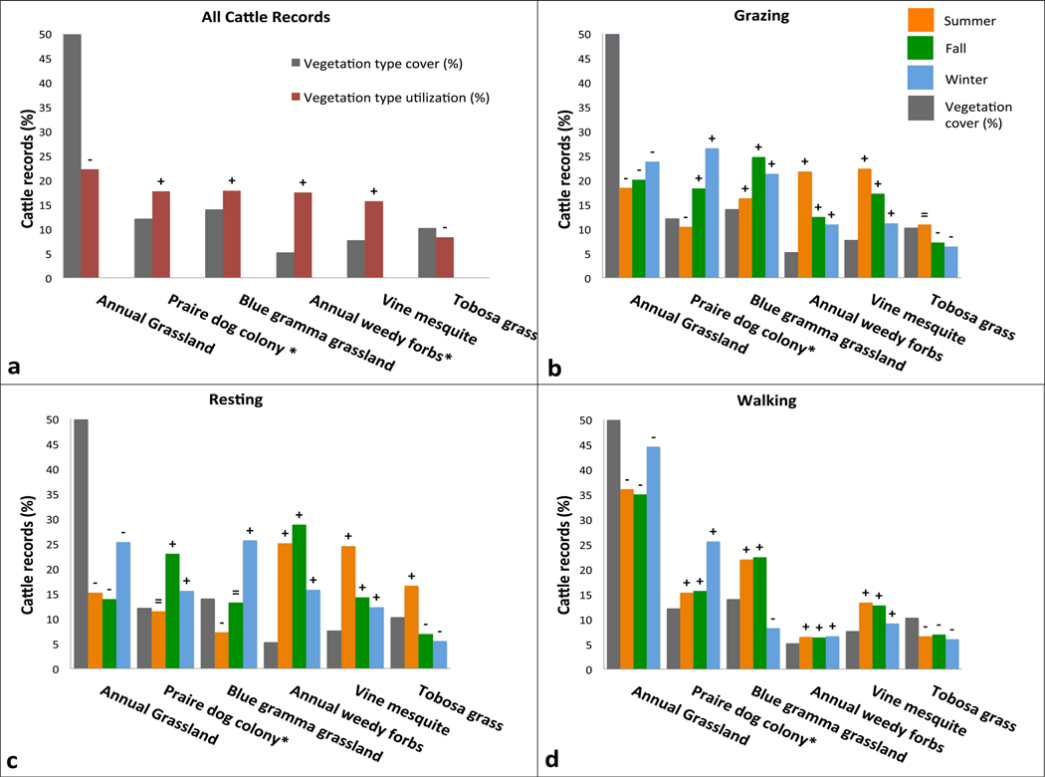
Large-scale experiment. Cattle record’s percentage by vegetation type, activity, and season. a) Total time cattle utilized the different vegetation types v.s. vegetation type % cover; b) Grazzing records by vegetation by season v.s. vegetation type cover %; c) Grazzing records by vegetation x season v.s. vegetation type cover %. d) Grazzing records by vegetation x season v.s. vegetation type cover %. Bonferroni confidence-interval results (P <0.001) are given by the following signs: “**+**” indicates preference (utilization above availability); “**=**” indicates random (utilization equal to its availability); and “–” indicates avoidance (utilization below its availability); and “*” indicates vegetation types with significant differences in utilization across seasons.

Cattle showed strong use preferences for prairie dog colonies, perennial gramma grassland, vine mesquite grassland and grassland dominated by annual weedy forbs (i.e., habitats used above their availability). In contrast, annual grassland and tobosa grassland were avoided (i.e., utilization below availability) (Fig. 1). Despite prairie dogs occupying only 12% of the landscape, cattle associated with colonies more than 24% of the total time; whereas, while annual grasslands covered 50% of the total area, cattle utilized them less than 20% of the time (Fig. 2 and Table 1).

**Table 1:** Large-scale experiment.

Large-scale cattle habitat preference experiment Bonferroni confidence-interval analysis						
Vegetation Type	Expected proportion	Confidence interval of occurrence (P> 0.001)	Season	Activity	Proportion observed	Utilization
Annual grassland	0.49	0.488 <P< 0.507	Summer	Grazing	0.18	-
				Resting	0.15	-
				Walking	0.35	-
			Fall	Grazing	0.19	-
				Resting	0.13	-
				Walking	0.34	-
			Winter	Grazing	0.23	-
				Resting	0.24	-
				Walking	0.44	-
Perennial gramma grassland	0.14	0.133 <P< 0.146	Summer	Grazing	0.16	+
				Resting	0.07	-
				Walking	0.21	+
			Fall	Grazing	0.24	+
				Resting	0.131	-
				Walking	0.22	+
			Winter	Grazing	0.21	+
				Resting	0.25	+
				Walking	0.08	-
Prairie dog colony	0.12	0.114 <P< 0.127	Summer	Grazing	0.10	-
				Resting	0.114	=
				Walking	0.15	+
			Fall	Grazing	0.18	+
				Resting	0.22	+
				Walking	0.15	+
			Winter	Grazing	0.26	+
				Resting	0.15	+
				Walking	0.25	+
Tobosa grassland	0.10	0.096 <P< 0.108	Summer	Grazing	0.108	=
				Resting	0.16	+
				Walking	0.06	-
			Fall	Grazing	0.07	-
				Resting	0.06	-
				Walking	0.06	-
			Winter	Grazing	0.06	-
				Resting	0.05	-
				Walking	0.05	-
Vine mesquite grassland	0.07	0.071 <P< 0.081	Summer	Grazing	0.22	+
				Resting	0.24	+
				Walking	0.13	+
			Fall	Grazing	0.16	+
				Resting	0.14	+
				Walking	0.12	+
			Winter	Grazing	0.11	+
				Resting	0.12	+
				Walking	0.90	+
Weedy annual forbs	0.05	0.047 <P< 0.056	Summer	Grazing	0.21	+
				Resting	0.25	+
				Walking	0.06	+
			Fall	Grazing	0.12	+
				Resting	0.28	
				Walking	0.06	+
			Winter	Grazing	0.10	+
				Resting	0.15	+
				Walking	0.66	+
Bare ground	0.008	0.007 <P< 0.010	Summer	Grazing	0.001	-
				Resting	0.0	-
				Walking	0.0	-
			Fall	Grazing	0.015	-
				Resting	0.009	=
				Walking	0.01	+
			Winter	Grazing	0.006	-
				Resting	0.01	+
				Walking	0.0	-

Cattle record’s percentage by vegetation type, activity, and season. Bonferroni confidence-interval results (P <0.001) are given by the following signs: “+” indicates preference (utilization above availability); “=” indicates random (utilization equal to its availability); and “–” indicates avoidance (utilization below its availability).

Cattle habitat selection also differed seasonally (MANOVA: P< 0.0001; F_2.8_ = 7.8e-5). Activity locations varied across the three seasons (MANOVA P< 0.035; F_1.6_ = 0.007) and activities (i.e., grazing, resting, walking) (MANOVA: P < 0.001; F_4.27_ = 0.03 (Fig. 1). The chi-square values for cattle activity by season were: Grazing, summer *X* ^*2*^ = 16,406.4, P = 0.05; fall *X* ^*2*^ = 8119.1, P = 0.05; winter *X* ^*2*^ = 8,282.67, P = 0.05; Resting, summer *X* ^*2*^ = 132,806; P = 0.05, fall *X* ^*2*^ = 36,234.4, P = 0.05; winter *X* ^*2*^ = 15,014.60, P = 0.05; Walking, summer *X* ^*2*^ = 32,806.1, P = 0.05; fall *X* ^*2*^ = 978.7, P = 0.05; winter *X* ^*2*^ = 1,724.6, P = 0.05 (Fig. 2 and Table 1). Seasonal changes in location x vegetation by activity, were detected only for prairie dog colonies (GLM: P = 0.02, F = 4.86; P 0.02, F = 8.40, respectively) and vine mesquite grassland (GLM: P = 0.05, F = 3.44; P <0.0001, F = 22.4, respectively). Use of annual weedy forbs differed by activity but not by season (GLM: P = 0.001, F = 10.32).

### Small-scale cattle grazing preference experiment

Cattle used prairie dog colonies for grazing and resting at rates above their availability during both the high forage availability season, in fall, and the grass dormancy season, in winter (P <0.001), (Fig. 2 and Table 1).

There were no significant differences among the three plots across the three blocks (MANOVA F_1.2_ = 0.79, P < 0.2), meaning no variation between treatments and prairie dog colonies, but strong seasonal preferences for particular foraging zones were detected (MANOVA F_4.5_ = 0.2035, P <0.0001). The chi-square values for cattle grazing locations by foraging zone were: *X* ^*2*^ = 619.2, summer; *X* ^*2*^ = 851.51, fall; *X* ^*2*^ = 2, 813.6, winter, denoting a strong non-random utilization pattern.

**Fig 3:**
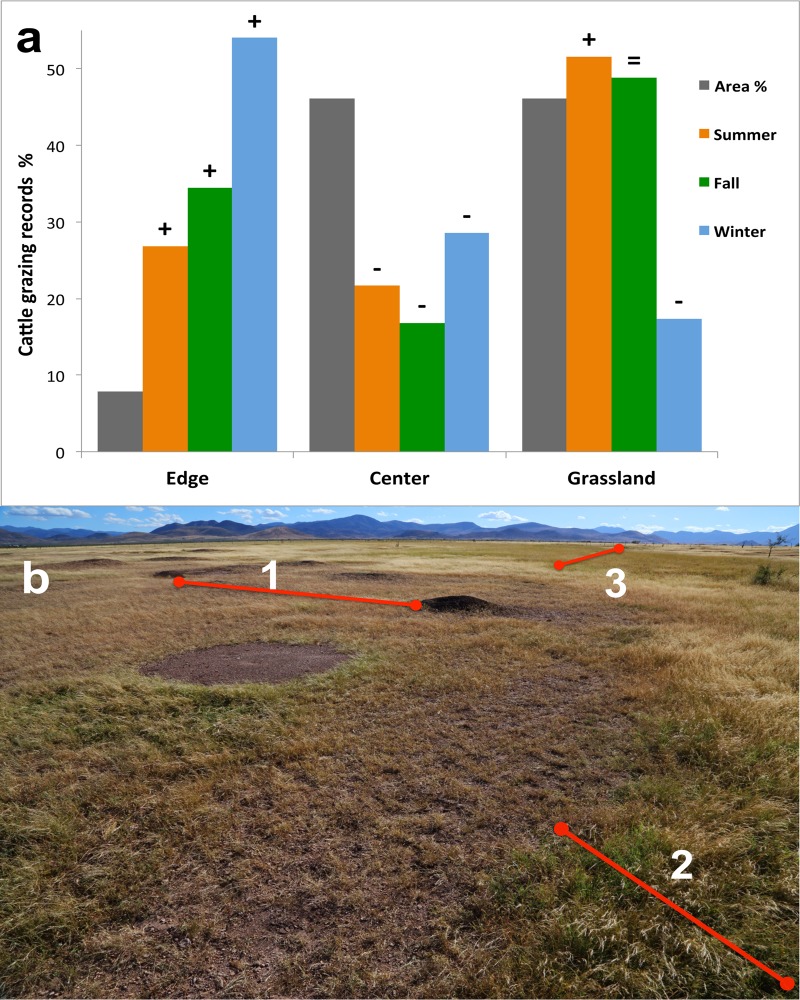
Small-scale experiment. Cattle grazing record’s percentage by foraging zone and season v.s. foraging zone area cover %. (a) Bonferroni confidence-interval results (P <0.001) are given by the following signs: “**+**” indicates preference (utilization above availability); “**=**” indicates random (utilization equal to its availability); and “–” indicates avoidance (utilization below its availability); (b) image of a typical prairie dog colony in the Janos grasslands, showing vegetation height and cover increasing away from the colony center (1) towards the colony edge (2) and the surrounding climax grasslands (3).

Prairie dog colony edges were preferentially selected for grazing by cattle across all seasons and were the most utilized foraging zone during the winter season (P< 0.001, Fig. 3, Table 2). More than 50% of the grazing events occurred in only 7% of the total experimental area, being represented by the colony edges. Unlike the colony edge, cattle consistently avoided grazing within the prairie dog colony center across all seasons (P < 0.001) (Fig. 3, Table 2).

**Table 2:** Small-scale experiment.

Small-scale cattle grazing preference experiment Bonferroni confidence-interval analysis					
Season	Foraging Zone	Expected Proportion	Proportion observed	Confidence interval of occurrence (P> 0.001)	Use
Summer *x* ^*2*^ = 619.2 P<0.005	Margin	0.071	0.268	0.048 <P< 0.094	+
	Center	0.463	0.216	0.419 <P< 0.509	-
	Grassland	0.463	0.515	0.419 <P< 0.509	+
Fall *x* ^*2*^ = 851.51 P<0.005	Margin	0.068	0.343	0.041 <P< 0.095	+
	Center	0.464	0.168	0.415 <P< 0.518	-
	Grassland	0.465	0.487	0.415 <P< 0.518	+
Winter *x* ^*2*^ = 2813.6 P<0.005	Margin	0.095	0.540	0.072 <P< 0.228	+
	Center	0.452	0.285	0.413 <P< 0.490	-
	Grassland	0.452	0.173	0.413 <P< 0.490	-

Cattle grazing record’s percentage by foraging zone and season v.s. foraging zone area cover %. Bonferroni confidence-interval results (P <0.001) are given by the following signs: “+” indicates preference (utilization above availability); “=” indicates random (utilization equal to its availability); and “–” indicates avoidance (utilization below its availability).

## Discussion

We found that cattle preferred foraging on prairie dog colonies in Chihuahuan desert grasslands, especially during the winter grass dormancy season but also during the fall. Results from this large-scale habitat selection experiment also were consistent with our small-scale grazing preference experiment on cattle foraging behavior in relationship to prairie dog colonies. During winter and fall seasons when cattle preferred prairie dog colonies for grazing, cattle spent most of their time grazing along the edges of colonies where forage quality is often higher than in off colony areas and where quantity is higher compared to colony centers [19,23,25]. In summer, when cattle did not prefer prairie dog colonies for grazing, cattle spent most of their foraging time off colonies, within the surrounding grassland, resembling the previously described American bison-prairie dog grazing association [7,12,18,19,23,25,27].

In our study, cattle grazed and rested on prairie dog colonies in the fall during the grass-growing season and especially in the winter during the grass dormancy season, suggesting that prairie dog colonies provide an important forage resource within the greater grassland landscape and during one of the harshest seasons for cattle grazing. These patterns probably resulted from prairie dog activities during the grass growing season that enhance forage quality, such as removal of standing dead biomass and clipping of vegetation that increases plant nitrogen uptake, in addition to the lack of other more palatable forage options during the winter season [20,52].

Foraging behavior is strongly influenced by dietary preferences, and large herbivores spend more time grazing in plant communities that have higher quantities of preferred forage [53]. While cattle showed strong preferences for foraging on prairie dog colonies in our study, they also associated with other vegetation types. They spent considerable time in perennial gramma grassland, vine mesquite grassland and annual weedy forb habitat, but avoided tobosa grassland and annual grassland, the latter being the most abundant vegetation type within the Janos region. In North American grasslands, cattle prefer perennial grasses like blue gramma (*Bouteluoa gracilis*) and vine mesquite (*Panicum obtusum*), which have their highest nutritional value during the summer and fall [49,54,55]. Annual grasses, such as the six weeks three awn have comparatively limited nutritional value; and other, more desert-adapted perennial grasses, such as tobosa grass, contains an abundant accumulated dead plant material which make them unpalatable [56].

Cattle foraging behavior varies seasonally with plant phenological and nutritional changes [10]. Perennial grasses and weedy annual forbs begin to green-up at the end of the spring into the beginning of summer, and are at this time, preferred forage by cattle. Growth of perennial grasses accelerates following the summer rains through fall, developing new foliage and pushing off dead remnants from the preceding dormancy season, increasing their palatability [57]; whereas in summer weedy forbs flower, form sharp spines and accumulate anti-herbivore compounds, so cattle avoid them [58]. During winter, nutritional quality of perennial grasses declines, reducing their consumption by cattle, and consumption of seasonal forbs increases [59]. Our findings, consequently, demonstrate the importance of heterogeneity within semi-arid grasslands, which includes prairie dog colonies, for the provision of multiple forage alternatives temporally and spatially within semi-arid grassland landscapes [34,60]. The preferential foraging on prairie dog colonies by cattle highlights not only the role of prairie dogs in creating heterogeneous grassland landscapes, [8,27] but also their important contribution to supporting local communities that depend on cattle grazing for their livelihoods.

Our results not only support previous studies showing that cattle occur more commonly on prairie dog colonies in the Chihuahuan Desert grasslands [40], but also that cattle preferentially forage on them. However, these ecological relationships between cattle and prairie dogs probably vary across the geographic ranges and different species of prairie dogs, and with variation in precipitation and plant productivity [52]. For example, in the mixed-grass prairie, cattle spend significantly more time in pastures with prairie dog colonies compared to pastures without colonies [39]. In contrast, cattle do not appear to associate with prairie dog colonies in shortgrass prairies [38]. When comparing the weights of cattle that graze in areas with prairie dog colonies (at < 30% pasture occupancy) and areas without prairie dog colonies, no significant difference in cattle weight gains have been found, presumably because the reduction of available forage on colonies is compensated for by the improved vegetation quality [44,45]. Nevertheless, weight gains can decline in shortgrass prairie when colonies occupy more than 30% of the total area, and when prairie dog colonies occupy more than 60% of the total area, cattle weight gain is reduced further (14%) [61,62].

Our work suggest that prairie dogs and cattle can have a positive, mutualistic relationship, in North America’s desert grasslands [8,37]. Cattle appear to benefit from modified vegetation structure and composition and increased nutritional value on prairie dog colonies [12,52,61]. Similarly, prairie dogs are known to benefit from the presence of large grazers, like bison and cattle [12,19,23,25,27,37]. For example, black-tailed prairie dog density increased about 2-fold under conservative cattle grazing, on a companion study located adjacent to ours [8]. Cattle grazing positively affected prairie dog abundances, likely by improving their ability to see predators [8,12,27,39]. Similar results have been reported for Utah prairie dogs (*C*. *parvidens*), which prefer foraging in areas grazed moderately by cattle compared to non-grazed areas [63]. Additionally, others have observed that prairie dogs establish their colonies in areas that are intensively grazed by livestock [39]. Similar to the effects of American bison, cattle stimulate nitrogen uptake and lower leaf age through their grazing, and increase available nitrogen by depositing dung and urine [13,34]. So, like bison, cattle also may positively impact prairie dogs by increasing forage quality [23,38,63,64]. This ecological relationship is similar to the grazing association between prairie dogs and bison [19,23,25].

### Management

This positive relationship between cattle and prairie dogs in the Chihuahuan Desert grasslands, challenges the long-standing view of prairie dogs as an undesirable pest species in rangelands [31]. The presence of prairie dogs can have a positive impact on cattle that is beneficial to the livestock industry, by prairie dog colonies providing favorable grazing habitat for cattle and reducing shrub invasion into grassland environments [22,28]. In turn, cattle can be used as a management tool to strategically graze areas where prairie dogs are needed to help promote biodiversity, enhance forage quality, and reduce shrub encroachment [34]. In sum, our work, along with that by Davidson, 2010, suggests that prairie dogs and cattle can have a mutualistic relationship. And, when their abundances are managed so that they interact synergistically together, they can enhance the productivity and biodiversity of grassland ecosystems [8], supporting local communities that depend on livestock grazing for their livelihoods and the livestock industry more generally.

A paradigm shift is needed on how rangelands are managed, from simply promoting maximization of livestock production and creating homogenous grassland landscapes dominated by only a few desirable forage species, to more integral management that benefits biodiversity, enhances habitat heterogeneity, and improves ecosystem services on which humans depend [34,60]. Given the widespread degradation of grasslands and loss to shrublands, the results of our work provide new insights into novel management strategies for grassland conservation and a potential win-win scenario for biodiversity and productive human activities. In addition to the possible direct benefits to cattle ranching and increasing grassland biodiversity, prairie dogs also increase groundwater recharge, forage availability, soil carbon storage, regulation of soil erosion, and regulation of soil productive potential [16]. Despite increasing awareness of the important functional role of prairie dogs, they are still considered a pest by range managers and still commonly subject to lethal control in both the US and Mexico [12]. Our research supports the argument that conservation and restoration of prairie dog populations should be key components of sustainable grassland management, and that conservation-guided cattle ranching can be a productive human enterprise, compatible with grassland biodiversity conservation objectives.

## Supporting Information

S1 File Norma Oficial Mexicana NOM-051-ZOO-1995, Trato humanitario en la movilización de animales. (PDF)

S2 File Norma Oficial Mexicana NOM-062-ZOO-1999, Especificaciones técnicas para la producción, cuidado y uso de los animales de laboratorio. (PDF)
