# Supplementary material for: Black-Tailed Prairie Dogs, Cattle, and the Conservation of North America’s Arid Grasslands
Source: PLoS One. 2015 Mar 11;10(3):e0118602. doi: 10.1371/journal.pone.0118602 (PMC4356543; doi:10.1371/journal.pone.0118602)

SECRETARIA DE AGRICULTURA, GANADERIA Y DESARROLLO RURAL

NORMA Oficial Mexicana NOM-051-ZOO-1995, Trato humanitario en la movilización de animales.

Al margen un sello con el Escudo Nacional, que dice: Estados Unidos Mexicanos.- Secretaría de Agricultura, Ganadería y Desarrollo Rural.

NORMA OFICIAL MEXICANA NOM-051-ZOO-1995, TRATO HUMANITARIO EN LA MOVILIZACION DE ANIMALES.

La Dirección General Jurídica de la Secretaría de Agricultura, Ganadería y Desarrollo Rural, con fundamento en los artículos 35 fracción IV de la Ley Orgánica de la Administración Pública Federal; 4o. fracciones I y III, 12 fracción XIV, 17 y 47 de la Ley Federal de Sanidad Animal; 1o., 38 fracción II, 40 fracciones III y XI, 41 y 47 fracción IV de la Ley Federal sobre Metrología y Normalización; 12 fracciones XXIX y XXX del Reglamento Interior de esta Dependencia, y

CONSIDERANDO

Que es función de la Secretaría de Agricultura, Ganadería y Desarrollo Rural fomentar programas y elaborar normas oficiales de sanidad animal, así como atender, supervisar y fomentar el trato humanitario a los animales durante su aprovechamiento, movilización y sacrificio.

Que los animales, cualquiera que sea su función (abasto, trabajo, compañía, exhibición u otra) o el motivo para su movilización (destino al sacrificio, cambio de instalaciones, traslado a exposiciones o atención médica), indistintamente de su edad, raza, sexo o condición física, se requieren movilizar bajo las mejores condiciones posibles que permitan su bienestar.

Que cuando las maniobras para la movilización de animales (desde el arreo, embarque, traslado y desembarque) son adecuadas según el caso, se disminuyen los factores de estrés que los hacen susceptibles a contraer enfermedades infecciosas.

Que los animales de abasto movilizados bajo condiciones mínimas de estrés y que favorezcan su bienestar, sufrirán menos traumatismos o golpes y habrá menos riesgo de muerte, y la calidad de sus productos y subproductos será mejor, evitándose pérdidas económicas por decomiso de canales provenientes de animales maltratados.

Que el objeto de las disposiciones señaladas en esta Norma es la implantación de sistemas y diseños para equipos de arreo, rampas, contenedores vehículos especializados para movilización de animales, que permitan cumplir cada vez mejor con todos los propósitos aquí mencionados y los que se adicionen en el futuro.

Que para alcanzar los objetivos señalados en los párrafos anteriores, con fecha 31 de octubre de 1996, se publicó en el **Diario Oficial de la Federación** el Proyecto de Norma Oficial Mexicana NOM-051-ZOO-1995, Trato humanitario en la movilización de animales, iniciando con ello el trámite a que se refieren los artículos 45, 46 y 47 de la Ley Federal sobre Metrología y Normalización, por lo que el 2 de enero de 1998, se publicaron las respuestas a los comentarios recibidos en relación a dicho Proyecto.

Que en virtud del procedimiento legal antes indicado, se modificaron los diversos puntos del proyecto que resultaron procedentes, por lo que a petición del Presidente del Comité Consultivo Nacional de Normalización de Protección Zoosanitaria se expide la presente Norma Oficial Mexicana, NOM-051-ZOO-1995, Trato humanitario en la movilización de animales, para quedar de la siguiente manera:

INDICE

1. Objetivo y campo de aplicación

2. Referencias

3. Definiciones

4. Requisitos generales para la movilización de animales domésticos

5. Requisitos particulares de movilización por especie

6. Requisitos para la movilización de fauna silvestre

7. Movilización de abejas productoras de miel (*Apis mellifera l.*)

8. Sanciones

9. Concordancia con normas internacionales

10. Bibliografía

11. Disposiciones transitorias

Apéndices normativos

1. Objetivo y campo de aplicación

1.1. La presente Norma Oficial Mexicana tiene como objetivo primordial establecer los sistemas de movilización de animales que disminuyan su sufrimiento, evitándoles tensiones o reduciéndolas durante todo el proceso.

1.2. La presente Norma Oficial Mexicana es de observancia obligatoria en todo el territorio nacional y es aplicable a la movilización de animales.

1.3. Las responsabilidades derivadas de esta Norma recaerán sobre el propietario de los animales que se movilicen, así como en la persona o empresa comercializadora, el transportista, el encargado de los animales o cualquier persona responsable de su movilización, según sea el caso.

1.4. La vigilancia de esta Norma corresponde a la Secretaría de Agricultura, Ganadería y Desarrollo Rural y a los Gobiernos de los Estados, en el ámbito de sus respectivas atribuciones y circunscripciones territoriales, y de conformidad con los acuerdos de coordinación respectivos.

1.5. La aplicación de las disposiciones contenidas en esta Norma, compete a la Dirección General de Salud Animal, así como a las Delegaciones Estatales de la Secretaría de Agricultura, Ganadería y Desarrollo Rural y a los Gobiernos de los Estados, en el ámbito de sus respectivas atribuciones y circunscripciones territoriales.

2. Referencias

Para la correcta aplicación de esta Norma deben consultarse las siguientes normas oficiales mexicanas:

NOM-024-Z00-1995, Especificaciones y características zoosanitarias para el transporte de animales, sus productos y subproductos, productos químicos, farmacéuticos, biológicos y alimenticios para uso en animales o consumo por éstos.

NOM-033-Z00-1995, Sacrificio humanitario de los animales domésticos y silvestres.

NOM-045-Z00-1995, Características zoosanitarias para la operación de establecimientos donde se concentren animales para ferias, exposiciones, subastas, tianguis y eventos similares.

NOM-059-ECOL-1994, Que determina las especies y subespecies de flora y fauna silvestre, terrestre y acuática, amenazadas, raras y aquellas sujetas a protección especial y que establece especificaciones para su protección.

NOM-011-SSA2-1993, Para la prevención y control de la rabia.

3. Definiciones

Para efectos de la presente Norma, se entiende por:

3.1. Abeja: Insecto himenóptero, *Apis mellifera l.*

3.2. Acopio: Acción de reunir o juntar a los animales.

3.3. Animales: Seres orgánicos que viven, sienten y se mueven voluntariamente o por instinto.

3.3.1. Anfibios: Clase de vertebrados que pueden vivir en medio terrestre o acuático de manera indistinta.

3.3.2. Domesticado (amansado o adiestrado): Animal sacado de su medio ambiente natural y que requiere del hombre para su subsistencia.

3.3.3. Domésticos: Todas aquellas especies que están bajo el cuidado del hombre.

3.3.4. Invertebrados: Aquellos animales que carecen de columna vertebral.

3.3.5. Mamíferos: Vertebrados que tienen glándulas mamarias para criar a su prole.

3.3.6. Reptiles: Clase de los vertebrados de respiración pulmonar que se desplazan arrastrándose y que poseen escamas en el cuerpo.

3.3.7. Venenosos: Aquellos que por contacto, piquete o mordida ocasionan trastornos al hombre y hasta pueden causarle la muerte.

3.4. Amarre: Diferentes nudos que se realizan para sujetar animales.

3.5. Arreo: Procedimiento por medio del cual se moviliza a los animales de un sitio determinado a otro.

3.6. Aves: Clase de vertebrados que se reproducen por medio de huevos, con cuerpo recubierto de plumas y que en su mayoría se desplazan volando.

3.6.1. Canoras: Las aves que se mantienen cautivas por lo melodioso de su canto.

3.6.2. De ornato: Aquellas aves que se mantienen cautivas por lo vistoso de su plumaje.

3.6.3. De presa: Especies de aves que poseen poderosas garras o picos para poder cazar su alimento; se incluyen todas las rapaces.

3.6.4. De corral: Aquellas aves que se crían para abasto, tales como gallinas, gansos, patos, guajolotes, entre otras.

3.7. Cama: Material que se pone en el suelo para que los animales pisen o se echen.

3.8. Certificado de salud: Documento en el que se asienta que un animal se encuentra clínicamente sano. Debe estar firmado por un médico veterinario que posea cédula profesional.

3.9. Certificado zoosanitario: Documento oficial expedido por la Secretaría de Agricultura, Ganadería y Desarrollo Rural o por quienes estén aprobados o acreditados para constatar el cumplimiento de las normas oficiales mexicanas. Tratándose de animales, será signado por un médico veterinario de la Secretaría o aprobado o acreditado.

3.10. Contenedores: Receptáculos con piso, paredes y techos sólidos, independientes del vehículo de transporte, diseñados para movilizar animales.

3.11. Corrales de descanso: Instalaciones bien acondicionadas para el reposo de los animales, con facilidades para alimentarlos y darles de beber y, en su caso, contar con los medios para realizar sacrificios de emergencia

3.12. Cuarentena: Medida zoosanitaria de control, basada en el aislamiento temporal, observación y restricción de la movilización de animales como medida profiláctica por sospecha o existencia de una enfermedad o plaga de los mismos.

3.13. Densidad de carga: Número de animales que se movilizan en condiciones óptimas en relación al volumen disponible del vehículo.

3.14. Duración del transporte: Tiempo total empleado en el traslado de los animales.

3.15. Embarcar: Acción de introducir a los animales en los vehículos o contenedores, para su movilización.

3.16. Embarcadero: Sitio destinado para embarcar.

3.17. Embarque: Número total de animales movilizados durante una maniobra de traslado.

3.18. Excreciones: Descarga o deposición de materia fecal, orina, vómito u otros.

3.19. Fauna silvestre: Todos aquellos animales que no son domésticos.

3.20. Fleje: Tira o banda de material resistente para sellar un contenedor.

3.21. Gestación: Tiempo que dura la preñez o embarazo.

3.22. Hacinamiento: Amontonamiento.

3.23. Haces luminosos: Conjunto de rayos emitidos o reflectados por una fuente luminosa.

3.24. Huella: Señal que deja un pie o pata o espacio para pisar.

3.25. Insensibilización: Acción con la que se induce rápidamente a un animal a un estado en el que no sienta dolor.

3.26. Jareta: Dobladillo donde pasa una cinta que amarra un costal.

3.27. Jaula: Armazón de madera, mimbre, alambre o barras de metal que sirve para encerrar animales y poderlos movilizar.

3.28. Jaula de abejas a granel: Embalaje para transporte cubierto en dos de sus caras con malla mosquitera.

3.29. Lesión: Daño o alteración morbosa, orgánica o funcional de los tejidos.

3.30. Manejo: Actividades o manipulación de los animales desde el acopio, embarque, movilización, hasta el desembarque.

3.31. Movilización: Traslado de animales de un lugar a otro por medio de un vehículo destinado para este fin, ya sea por vía terrestre, marítima, aérea o a pie.

3.32. Núcleos de abejas: Conjunto de abejas en desarrollo que consta de 2 a 5 bastidores, cría, abejas adultas y reservas alimenticias.

3.33. Peralte: Altura en relación con el piso.

3.34. Perchas: Barras de madera o cualquier otro material que imite superficies naturales para que las aves posen sus patas y descansen sobre ellas. Deben atravesar de un lado a otro del contenedor o jaula y tener un diámetro que corresponda al tamaño de las patas de las aves movilizadas, para las aves que así lo requieran.

3.35. Periodo de descanso: Comprende desde el momento en que ha sido desembarcado el último animal, hasta el momento en que se embarca de nuevo al primero. Si los vehículos están construidos para fines de traslado, el descanso de los animales puede permitirse en el interior de los mismos.

3.36. Periodo de movilización: Tiempo de permanencia de los animales dentro del vehículo durante su movilización.

3.37. Persona o empresa comercializadora: Persona física o moral que realiza actividades de compra y venta de animales, que para lo cual tiene que movilizarlos.

3.38. Propietario: Dueño de un animal.

3.39. Punto de verificación: Sitio aprobado por la Secretaría de Agricultura, Ganadería y Desarrollo Rural, donde se constata el cumplimiento de las normas oficiales mexicanas vigentes en materia de salud animal a través de la inspección documental y/o física.

3.40. Sacrificio de emergencia: Sacrificio necesario con métodos humanitarios que se practica en cualquier animal que haya sufrido lesiones traumáticas o afecciones que le causen dolor y sufrimiento; o bien para aquellos animales que al estar fuera de control puedan causar algún daño o lesión al hombre u otros animales.

3.41. Salud: Estado normal de las funciones orgánicas de los animales.

3.42. Secretaría: La Secretaría de Agricultura, Ganadería y Desarrollo Rural.

3.43. Trato humanitario: Conjunto de medidas para disminuir la tensión, sufrimiento, traumatismos y dolor de los animales durante su captura, movilización, exhibición, cuarentena, comercialización, aprovechamiento, entrenamiento y sacrificio.

3.44. Traumatismo: Acción violenta del exterior que produce una lesión local o general y que puede causar hasta la muerte.

3.45. Trayecto: Espacio a recorrer entre un punto y otro.

3.46. Toldo: Cubierta de lona o hule sostenida sobre un camión por medio de arcos para resguardar su contenido del sol o la lluvia.

4. Requisitos generales durante la movilización de animales

4.1. Referente a los animales.

4.1.1. Durante todas las maniobras de movilización, la seguridad y comodidad con que se manejen y viajen los animales, son factores de atención prioritaria.

4.1.2. Por lo general no se debe restringir a los animales el consumo de alimento y agua antes de su movilización, a excepción de los casos mencionados en los capítulos que tratan de cada especie.

4.1.3. No debe ser movilizado ningún animal que no pueda sostenerse en pie, que se encuentre enfermo, herido o fatigado, a menos que la movilización sea por una emergencia o para que los animales reciban tratamiento médico y siempre que su movilización no represente un riesgo zoosanitario. En caso de hembras no se movilizarán cuando se tenga la certeza de que el parto ocurrirá durante el trayecto.

4.1.4. No deben movilizarse crías de animales que para su alimentación y cuidados aún dependan de sus madres, a menos que viajen acompañadas por ellas.

4.1.5. Cuando los animales se movilicen en grupos no homogéneos se deben subdividir en lotes, ya sea según especie, sexo, edad, peso o tamaño, condición física, función zootécnica o temperamento, y si se alojan en el mismo vehículo se usarán divisiones en su interior.

4.1.6. En el caso de las especies ectotermas como invertebrados, anfibios y reptiles, se tomarán las precauciones necesarias para facilitar la ventilación y mantenimiento de los animales en temperaturas cercanas al rango óptimo para cada caso en particular. En el caso de las especies endotermas como las aves y mamíferos, no serán transportadas en condiciones climáticas extremas.

4.2. Referente al manejo.

4.2.1. El manejo comprende todas las maniobras necesarias para la movilización de los animales, que incluyen: el acopio, arreo, enjaulado, embarco, traslado y desembarco, que en todos los casos se realizarán con precaución y con calma.

4.2.2. El periodo de movilización comprende desde el momento en que se embarca al primer animal, hasta el momento en que se ha desembarcado al último. Los periodos máximos de confinamiento de los animales en vehículos equipados con bebederos o comederos, o que permitan el descanso en su interior, están recomendados en los capítulos de la especie que se trate.

4.2.3. En los puntos de inspección sanitaria los trámites de inspección deberán ser expeditos, ya que si hay aglomeración de vehículos, el periodo de espera afecta la condición de los animales.

4.2.4. De preferencia, los responsables del manejo serán cuidadores o vaqueros a los que estén acostumbrados los animales y los reconozcan fácilmente.

4.2.5. Los responsables del manejo para la movilización de los animales, deben mantenerlos tranquilos en todo momento, actuando sin brusquedad, evitando hacer ruido excesivo o dar gritos o golpes, para que los animales no sufran tensión ni se lastimen, agredan o peleen.

4.2.6. Durante el arreo no debe golpearse a los animales con ningún objeto que pueda causarles traumatismos.

4.2.7. Las maniobras de embarco y desembarco de animales deberán hacerse bajo condiciones de buena iluminación, tanto dentro como fuera del vehículo. Se debe evitar durante estas maniobras el contraste brusco entre la luz y la oscuridad, o dirigir haces luminosos de luz directamente a los ojos de los animales.

4.2.8. Para la maniobra de embarco y desembarco de animales, el vehículo se debe retroceder lentamente, cuidando que no quede espacio entre su piso y la rampa, donde puedan quedar atrapadas las patas de los animales, evitando así que se caigan o fracturen.

4.2.9. No deben sobrecargarse con animales los vehículos de movilización, debiendo respetarse las densidades de carga indicadas para cada especie animal en el capítulo correspondiente.

4.2.10. Para movilizar en el mismo vehículo a uno o varios animales de diferente procedencia, tamaño, condición física, edad o sexo, se debe contar con suficientes divisiones que permitan separarlos dentro del vehículo, según sea el caso.

4.2.11. En caso de tener que amarrar a los animales para su movilización, nunca se sujetarán por las patas ni se utilizarán nudos corredizos que puedan causar su estrangulación.

4.2.12. Deben inspeccionarse los animales periódicamente a lo largo del recorrido, para detectar aquellos que estén echados o caídos, tratando de evitar que sean pisoteados o sufran mayores lesiones, como hematomas o fracturas.

4.2.13. Cuando se amerite un sacrificio de emergencia deberá procederse conforme a la Norma NOM-033-ZOO-1995, Sacrificio humanitario de los animales domésticos y silvestres, en los capítulos correspondientes.

4.2.14. Si el trayecto durante la movilización es largo, se recomiendan periodos de descanso, con o sin desembarco de los animales para que reciban agua o alimento periódicamente, según lo señalado en los capítulos correspondientes.

4.2.15. En el caso de vehículos equipados adecuadamente para abrevar y alimentar a los animales en su interior, los periodos de descanso durante el trayecto se deben cumplir siempre con el vehículo estacionado bajo la sombra.

4.2.16. Solamente se desembarcan a los animales para que descansen durante el trayecto cuando el certificado zoosanitario vigente para esa movilización así lo permita, y si además existen lugares apropiados o corrales de descanso a lo largo del camino, que estén aprobados por la Secretaría.

4.2.17. Los tiempos de espera de animales de abasto en los rastros, deberán cumplirse con los vehículos estacionados bajo la sombra, y para disminuir los tiempos de espera en los rastros:

a) El arribo de los animales deberá programarse cada vez que sea posible.

b) Los rastros deberán contar con suficientes corrales de desembarco.

4.2.18. Para movilizar ganado que recientemente haya sido sumergido en agua o baño garrapaticida, deberá dejarse escurrir a los animales antes de ser embarcados. No deben embarcarse nunca animales aún mojados cuando se vayan a movilizar bajo condiciones de clima frío.

4.2.19. Nunca se deben movilizar animales junto con sustancias en el mismo vehículo, y especialmente cuando éstas sean tóxicas o peligrosas.

4.3. Referente a los vehículos, contenedores y jaulas.

4.3.1. La movilización de los animales, ya sea por vía terrestre carretera o ferrocarril, aérea o marítima, debe realizarse con vehículos y/o contenedores diseñados o adaptados para este fin.

4.3.2. La movilización aérea de cualquier especie animal se hará de acuerdo con las normas establecidas por la International Air Transport Association (IATA).

4.3.3. La selección del tamaño, diseño, material y resistencia del vehículo, contenedor o jaula más apropiado, debe hacerse con base en la especie, número, tamaño, edad, sexo, fin zootécnico o comportamiento de los animales que se vayan a movilizar, incluyendo un método seguro para mantenerlo cerrado para evitar escapes de los animales o accidentes a terceras personas.

4.3.4. Los contenedores o jaulas deberán sujetarse firmemente a los vehículos durante su movilización, para evitar:

a) Que se muevan durante el viaje y se lesionen los animales ocupantes.

b) Que cualquier objeto alrededor obstaculice su ventilación o caiga sobre ellos.

Ejemplo: Jaulas de aves u otros animales que van sobre plataformas.

4.3.5. En el interior de vehículos, contenedores o jaulas no deben existir clavos, alambres, salientes, pasadores o cualquier objeto punzocortante que pueda lesionar a los animales durante el manejo.

4.3.6. Los vehículos para la movilización de animales deberán contar con mantenimiento adecuado para evitar descomposturas durante el trayecto.

4.3.7. Los vehículos o contenedores estarán diseñados y construidos de manera que:

a) Los animales sean embarcados y desembarcados fácilmente.

b) La ventilación sea de acuerdo con el clima y requerimientos de las especies animales de que se trate.

c) Sean fáciles de limpiar.

d) Estén equipados con rampas portátiles o dispositivos de emergencia que permitan el desalojo rápido de los animales.

4.3.8. El piso deberá ser antiderrapante y estar en buenas condiciones; si no permite el drenaje y para absorber las excreciones, antes de embarcar a los animales, se acondicionará cubriéndolo con un material de cama limpio y seco, como arena, paja o aserrín, cuya cantidad será proporcional a la duración del viaje para prevenir superficies húmedas o lodosas antes de concluirlo.

4.3.9. Si no tienen techo, deben contar con sistemas de cobertura como lonas o toldos, con la finalidad de proteger a los animales del sol, del frío y la lluvia, cuando se requiera.

4.3.10. Las separaciones físicas en el interior de los vehículos, para la movilización de lotes de animales, deben ser de material resistente y estar bien sujetas en el interior para evitar que se muevan o sean derribadas fácilmente por los propios animales.

4.3.11. Los interiores de los vehículos deberán tener la altura suficiente para prevenir golpes en la cabeza y el dorso de los animales que viajan, y además deben estar diseñados de tal forma que no haya escurrimientos de orina, estiércol, vómitos u otras excreciones. En el caso de vehículos de 2 pisos, los animales más pesados se colocarán en el piso de abajo y los más ligeros en el piso superior.

4.4. Referente a las rampas y embarcaderos.

4.4.1. Los embarcaderos deben estar ubicados en lugar accesible en el corral de manejo o cerca de éste, para evitar arreos innecesarios y manejo excesivo.

4.4.2. En aquellos lugares donde no coincidan la altura del piso del vehículo y de las rampas fijas de embarcaderos, deberán existir rampas móviles.

4.4.3. Características de las rampas fijas:

a) En el extremo más alto debe haber una plataforma de aproximadamente 2 metros de largo.

b) La altura de los pisos de la rampa y del vehículo deben coincidir.

c) Las paredes serán muy sólidas y el piso firme y antiderrapante.

d) El ancho de la plataforma y las rampas, entre las paredes, será aproximadamente de 2.5 m para desembarcar y guiar al ganado cómodamente.

e) Las rampas de concreto se recomienda que tengan escalones aproximadamente de 10 cm de altura o peralte y 30 cm de ancho o huella. Cada escalón debe tener algunas canaladuras profundas o el piso antiderrapante.

f) Su inclinación no debe exceder los 20 grados.

4.5. Referente al transportista, el recorrido y la documentación.

4.5.1. El chofer debe contar con experiencia para manejar el vehículo y la carga que transporta, conduciendo con precaución, evitando arrancar y detenerse bruscamente y siempre deberá ir acompañado de un asistente.

4.5.2. Debe llevar consigo la siguiente documentación:

a) Licencia de manejo vigente, que corresponde al tipo de vehículo que conduce y acorde al servicio prestado.

b) Los datos y dirección completos del destinatario del embarque.

c) Los datos y manera de comunicarse, ya sea con el propietario o el destinatario de los animales que moviliza, para avisar sobre cualquier emergencia.

d) Todos los documentos o certificados correspondientes a los requisitos de la normatividad oficial vigente de la especie animal que se moviliza.

e) Debe contar con un registro para el control de los tiempos de recorrido durante la movilización.

4.5.3. El chofer se debe cerciorar de que las chapas o pasadores de las puertas del vehículo sean seguros y estén bien cerrados antes de iniciar la marcha.

4.5.4. El transportista debe evitar las paradas innecesarias. En caso de que el vehículo se detenga por causas de fuerza mayor y el viaje no pueda reanudarse, se debe desembarcar a los animales o solicitar un reemplazo de vehículo, siempre y cuando el certificado zoosanitario de movilización lo permita. Cuando las disposiciones zoosanitarias impidan el desembarco de los animales por viajar en un vehículo con fleje, se dará aviso a la autoridad de sanidad animal más próxima para observar las normas correspondientes.

4.6. Referente a las condiciones durante estancias de animales en terminales de viaje, puertos y aduanas.

4.6.1. En el caso de animales que sean retenidos por más de 12 horas durante el trayecto de su movilización o al arribar a su destino, ya sea por razones fortuitas de decomiso, administrativas o accidentales, se les debe proporcionar alojamiento amplio y ventilado, agua suficiente para beber y alimentación propia de su especie hasta que sea solucionado el problema y puedan proseguir su destino, o sean rescatados y devueltos, o bien entregados a instituciones autorizadas para su custodia y disposición.

4.6.2. Estos animales, en ningún caso deben alojarse junto a la carga general, debiendo habilitarse en los lugares mencionados un espacio previsto para su estancia, en el cual existan las condiciones arriba señaladas.

5. Requisitos particulares de movilización por especie

En todos los casos que aplique, habrán de considerarse además los incisos correspondientes a los puntos 4.1., 4.2., 4.3., 4.4. y 4.5. de esta Norma.

5.1. Movilización de aves domésticas.

5.1.1. Para su manejo y movilización no se colgará nunca a las aves de sus alas, ni se les mantendrá amarradas de las patas.

5.1.2. Las jaulas o contenedores con aves no se deben arrojar ni azotar, evitando su excesiva manipulación, procurando manejarlas con sistemas mecánicos.

5.1.3. Cuando se movilicen grupos de aves en una misma jaula, deberá evitarse en todos los casos su hacinamiento dentro de la misma.

a) La mayoría de las jaulas para movilizar aves tienen una altura en su interior que impide que viajen paradas, deberán contemplarse diseños más altos para permitir que puedan pararse sin que tope su cabeza con el techo de la jaula.

b) Si el piso de la jaula cuenta con orificios, éstos tendrán una forma y tamaño que impida que los dedos y patas de las aves se atoren en ellos.

5.1.4. Pollos y gallinas: La mayor proporción de aves que se movilizan son los pollos y gallinas con destino al rastro.

a) Previo al momento del sacrificio se deberá considerar un tiempo de ayuno de 10 horas en promedio, para disminuir la contaminación de las canales por el contenido de alimento aún presente en el aparato digestivo.

b) El tiempo de ayuno incluye:

- Periodo de restricción al acceso de alimento en la granja.

- Tiempo que dure el acopio de las aves.

- Enjaulado.

- Traslado y tiempo de espera del vehículo en andén del rastro.

- Desembarco y periodo de sacrificio.

c) El periodo de movilización para estas aves no debe ser mayor de 12 horas.

No se recomiendan periodos de descanso durante la movilización de aves, ya que no se obtienen beneficios.

d) Las jaulas para movilización de pollos y gallinas por lo general son de las siguientes dimensiones: 96 cm de largo, 57 cm de ancho y 23-26 cm de altura libre en el interior. En este tipo de jaulas no deberán introducirse más de 10 pollos o gallinas (con promedio de peso de 2.5 Kg).

e) Las jaulas con pollos y gallinas se movilizarán siempre sobre plataformas planas y se acomodarán apiladas en columnas, ya que por su diseño se ensamblan unas con otras, lo que favorece que se mantengan fijas y se muevan menos durante el trayecto.

f) Si se movilizan jaulas apiladas, éstas deberán tener un diseño con salientes a los lados, de manera que al ensamblarlas queden espacios de cerca de 4 cm a todo su alrededor para permitir el paso del aire entre ellas y hacia su interior.

g) Para favorecer aún más la ventilación no se formarán columnas de más de 4 jaulas de ancho y entre cada columna se respetarán espacios por lo menos de 15 cm de separación.

5.1.5. Pollitos recién nacidos.

Al salir de las plantas de incubación se movilizan gran cantidad de pollitos de 1 a 2 días de nacidos, en cajas que varían en tamaño y construcción.

a) No deben movilizarse más de 100 pollitos/caja.

b) El interior de cada caja contará con separadores fijos que permitan subdividirla al menos en cuatro compartimentos para evitar que se hacinen en las esquinas.

c) Las dimensiones mínimas en centímetros para las cajas serán: 56 X 46 X 15.

d) El fondo de las cajas se debe recubrir de un material inocuo y absorbente.

e) La movilización más frecuente se hace en camiones y por vía terrestre, debiendo contar los vehículos, en todos los casos, con ventiladores especiales con tomas y salidas de aire, o con sistema de aire acondicionado, pudiendo requerirse en verano hasta sistemas de refrigeración para contrarrestar la generación de calor cuando el embarque es de miles de pollitos.

f) No se recomienda movilizar pollitos recién nacidos por periodos más largos de 16 horas.

g) Para movilización aérea de pollitos se usan las mismas cajas y solamente viajarán un máximo de 85 pollitos/caja.

5.1.6. Pavos, patos, gansos, codornices y otras aves de corral.

Para su movilización se requieren jaulas con diseño y dimensiones de acuerdo al tamaño y número de aves a movilizar, evitando en todos los casos el hacinamiento.

5.2. Movilización de ganado bovino.

5.2.1. El periodo de movilización para el ganado bovino no debe exceder de 18 horas sin descanso y sin darles agua de bebida.

a) Los periodos de descanso sin desembarcar al ganado durante los viajes por vía terrestre, deben ser por lo menos de 3 horas y conforme a los incisos 4.2.13. y 4.2.14. de esta Norma.

b) En el caso de movilizaciones más prolongadas de 24 horas, además de los descansos cada 18 horas, se les ofrece alimento a los animales.

c) Las vacas en producción o recién paridas deben ser ordeñadas cada 12 horas.

5.2.2. En el caso de vacas gestantes dentro de los dos primeros tercios de la gestación y de becerros hasta de 6 meses de edad, no deben movilizarse por más de 8 horas sin descanso, y sin ofrecerles agua y alimento.

5.2.3. Los sementales deben movilizarse individualmente o separados del resto de los animales por medio de divisiones en el interior del vehículo.

5.2.4. En vehículos o contenedores con techo, el espacio mínimo entre el piso y techo será de aproximadamente un tercio más alto de la altura promedio a la cruz de los animales del embarque (ejemplo: altura promedio a la cruz: 1.50 m = 2.00 m espacio interior del piso al techo).

5.3. Movilización de ovinos y caprinos.

5.3.1. No debe usarse el bastón eléctrico para el arreo de ovinos y caprinos, y durante su manejo se evitará en todos los casos levantarlos tomándolos de la lana.

5.3.2. Las jaulas o contenedores para movilizarlos deben tener una altura mínima en el interior de 1.10 m.

5.3.3. Los ovinos machos o moruecos y los machos cabríos adultos, deben movilizarse individualmente y si corresponden al mismo hato pueden viajar en grupo.

5.3.4. Todas las maniobras de embarque de ovinos o caprinos deben hacerse utilizando rampas con las características señaladas en el inciso 4.4. de esta Norma.

5.3.5. El periodo de movilización no debe exceder de 18 horas sin periodos de descanso y sin dar de beber a los animales.

a) Los periodos de descanso sin desembarcar al ganado durante los viajes por vía terrestre, deben ser por lo menos de 2 horas y conforme a los incisos 4.2.13 y 4.2.14 de esta Norma.

b) Los corderos y cabritos deben recibir agua y alimento cada 8 horas durante los periodos de movilización.

5.4. Movilización de porcinos.

5.4.1. La mayoría de los cerdos que se movilizan son animales para pie de cría o de abasto con destino al rastro, si son adultos, toleran bastante bien las temperaturas frías, en cambio el calor resulta un inconveniente, y si es extremo, se recomienda darles un baño de aspersión durante viajes largos, y aquí la presencia de lluvia resultaría conveniente, como una excepción de lo recomendado en el inciso 4.1.5.

5.4.2. El periodo de movilización en cerdos no debe exceder de 20 horas.

a) El periodo de movilización sin descanso no debe exceder de 8 horas.

b) Los periodos de descanso durante el trayecto se cumplirán por lo menos cada 8 horas con los animales dentro del vehículo, estacionándolo bajo la sombra por periodos que no excederán de 1 hora.

5.4.3. Los pisos de vehículos para movilización de cerdos se recomienda que cuenten con una serie de soleras o tiras de metal con bordes redondeados de 2-3 cm de alto bien fijas y que corran a lo ancho y largo del camión, con la finalidad de favorecer el apoyo de las patas para evitar que resbalen y para favorecer que se levanten, disminuyéndose así las pérdidas por fracturas y golpes.

5.4.4. El espacio mínimo recomendado para movilizar cerdos es de 0.45 m2 por cerdo con promedio de 100 Kg de peso vivo.

5.5. Movilización de equinos.

5.5.1. No se permite la utilización de vehículos de 2 pisos para su movilización.

5.5.2. Los vehículos o remolques deben ser lo suficientemente largos para que los animales quepan cómodamente.

5.5.3. El ancho y largo de las paredes de los remolques para la movilización de equinos será:

Peso promedio del animal en kgs	Espacio entre las paredes en cm	Largo mínimo del remolque en cm
menos de 500	67	2.50
más de 500	81	3.00

5.5.4. La altura mínima del techo de los vehículos para movilizar equinos será de 75 cm por encima de la cruz de los animales más altos.

5.5.5. La altura mínima de las paredes de vehículos sin techo debe ser suficiente para evitar en todos los casos que los animales puedan brincar. Se recomienda que las paredes lleguen a la altura del cuello.

5.5.6. Para arrear a los equinos nunca se debe usar el bastón eléctrico.

5.5.7. Los garañones deben ser movilizados en forma individual o separados por medio de divisiones del resto de los animales.

5.5.8. Los burros deben ser movilizados separadamente de los caballos y las mulas.

5.5.9. Los equinos deben ser movilizados por periodos máximos de 18 horas, seguidos de un periodo de descanso de 8 horas realizado de preferencia en instalaciones adecuadas.

5.5.10. Si la movilización es más prolongada, los equinos deben viajar por periodos hasta de 12 horas, permitiéndose paradas cada 6 horas, con duración de una hora, para proporcionarles agua y alimento.

5.5.11. Para la movilización de grupos de equinos sueltos, se dividirán en lotes homogéneos según el sexo, peso o alzada.

5.5.12. A los equinos movilizados en grupo para su venta y/o sacrificio, se les deben quitar las herraduras de los cuatro cascos antes de iniciar el viaje para que no se resbalen o evitar que se dañen unos a otros.

5.6. Movilización de perros y gatos.

5.6.1. Todos los perros y gatos deben ser movilizados en jaulas adecuadas excepto cuando van acompañados de sus dueños en vehículo particular evitando movilizarlos en espacios muy reducidos o en posturas incómodas.

5.6.2. Es una práctica frecuente que los dueños dejen a sus perros y/o gatos en el interior de sus vehículos estacionados. En estos casos se sugiere lo siguiente:

a) Procurar que el tiempo de permanencia del animal en el vehículo sea mínimo.

b) Siempre que sea posible, estacionar el vehículo en la sombra.

c) Abrir por lo menos dos ventanas del vehículo a una altura que permita la entrada de aire y a la vez evite que el animal escape.

5.6.3. Queda prohibido movilizar perros o gatos dentro de cajuelas, aun en trayectos muy cortos para evitar cualquier riesgo de asfixia o sobrecalentamiento.

5.6.4. El tamaño de las jaulas debe ser suficiente para que el animal pueda moverse libremente en su interior y recostarse en una posición natural.

**Tipo de animal**		**Dimensión mínima establecida de la jaula**		
Gatos	Perros	Ancho	Largo	Altura
chicos	muy chicos	30 cm	50 cm	37 cm
grandes	chicos	42 cm	60 cm	30 cm
	medianos	52 cm	75 cm	50 cm
	grandes	57 cm	87 cm	65 cm
	muy grandes	65 cm	1.10 m	75 cm

5.6.5. Las jaulas deben estar construidas con materiales resistentes e impermeables, provistas de orificios en las paredes y/o techo que permitan una suficiente ventilación, con una puerta de acceso fuerte y resistente, cerrada firmemente para evitar que el animal escape.

5.6.6. El piso debe cubrirse durante la movilización con varias capas de papel periódico que permitan la absorción de las excretas y su eliminación periódica.

5.6.7. Los perros y gatos no deberán permanecer más de 24 horas sin ingerir alimento durante los periodos de movilización.

5.6.8. En el caso de movilizaciones durante más de 6 horas, se debe fijar fuertemente al interior de la jaula un receptáculo que contenga agua potable. La forma y material del bebedero deben impedir que el agua se vacíe y que el animal se lastime.

5.6.9. En el caso de perros capturados en cercos epidemiológicos, su transporte se hará de acuerdo a los reglamentos que sobre los centros de control canino, expidan los servicios estatales de salud.

5.7. La jaula o contenedor debe contar siempre con una identificación o etiqueta visible y bien adherida que cuente con la siguiente información:

* Datos del destinatario y del remitente.

* Contenido: nombre común y científico del animal y número de ejemplares.

* Datos relevantes acerca de la temperatura o alimentación para el mantenimiento de los ejemplares durante el periodo de movilización.

* Indicaciones especiales, como ejemplo: productos utilizados para sedación.

* Documentación que se acompaña.

* Flechas dibujadas que señalen la posición correcta de la jaula o contenedor.

* Leyendas de importancia como: "Animales vivos", "Manejar con cuidado".

6. Movilización de fauna silvestre

Se ubican dentro de la fauna silvestre los siguientes:

Invertebrados: terrestres como tarántulas, alacranes, escarabajos, entre otros.

Anfibios: como ranas, salamandras, ajolotes, entre otros.

Reptiles: como víboras, cocodrilos, tortugas, saurios, entre otros.

Aves: canoras y de ornato, de presa, palmípedas y corredoras.

Mamíferos: carnívoros, herbívoros, insectívoros tanto terrestres como voladores.

a) Para evitar que los animales escapen o produzcan lesiones al personal que los maneja, en todos los casos deberán tomarse las medidas de seguridad pertinentes.

b) Cualquier contenedor donde se movilicen animales silvestres de preferencia se colocará en lugares oscuros o con poca luz para disminuir el estrés durante el viaje, pero siempre evitando los objetos que obstaculicen su ventilación o que pudieran caer sobre él.

c) Cualquier caja, jaula o contenedor donde se movilicen animales silvestres debe permitir una abundante ventilación de los ocupantes en su interior; deben contar con orificios en la tapa y en las paredes, los cuales serán de un diámetro tal que impida la salida de la cabeza o de alguna extremidad.

d) En todos los casos cuando se movilicen animales silvestres en grupo deberá evitarse el hacinamiento.

e) La jaula o contenedor debe contar siempre con una identificación o etiqueta visible y bien adherida que cuente con la siguiente información:

* Datos del destinatario y del remitente.

* Contenido: nombre común y científico del animal y número de ejemplares.

* Datos relevantes acerca de la temperatura o alimentación para el mantenimiento de los ejemplares durante el periodo de movilización.

* Indicaciones especiales, como ejemplo: productos utilizados para sedación.

* Documentación que se acompaña.

* Flechas dibujadas que señalen la posición correcta de la jaula o contenedor.

* Leyendas de importancia como: "Animales vivos", "Manejar con cuidado" u otros.

* Cuando se movilizan animales venenosos, debe incluirse una leyenda visible que diga: "Peligro Venenoso", como se indica en el Apéndice A (Normativo)

* En su caso, incluir una leyenda de "animales peligrosos".

* Incluir una leyenda de "fauna silvestre".

6.1. Invertebrados terrestres, como se indica en el Apéndice B (Normativo)

a) Deben movilizarse en cajas con divisiones para cada uno de los individuos, ya que la mayoría de estas especies son de hábitos carnívoros.

b) Si el viaje es prolongado de más de 24 horas, se debe colocar en una esquina de cada división de la caja una esponja con tamaño similar al del cuerpo del animal, embebida con agua potable colocada sobre una superficie impermeable para que puedan beber.

c) En el caso de caracoles para consumo, se deben transportar en cajas con cama de salvado o aserrín debidamente ventiladas y permitiéndoles libertad de movimiento.

6.2. Anfibios de abasto (Ranas, ajolotes).

6.2.1. Pueden viajar en cajas de unicel o de madera que tengan en las paredes y la tapa un buen número de orificios para lograr la mayor ventilación posible. El piso debe ser de consistencia suave o lisa e impermeable cubierta la mitad de una capa de hule espuma húmedo para que los animales se posen en la parte seca o húmeda cuando deseen. Sólo se introducirá el número de animales que ocupe la mitad de la superficie del suelo. Los animales deben movilizarse con ayuno de 24 horas, para disminuir la cantidad de excretas. Las cajas deben estar firmemente cerradas, como se indica en el Apéndice C (Normativo).

6.3. Reptiles.

Deben movilizarse con ayuno de 24 horas cumpliendo con lo indicado en el punto 4.1.6. de esta Norma.

6.3.1. Tortugas y saurios: Se colocan en cajas de unicel o madera con piso liso de textura suave. Si se movilizan animales muy grandes, de preferencia deben viajar en forma individual, la caja debe contar con varios orificios, como se indica en el Apéndice D (Normativo).

6.3.2. Serpientes y saurios venenosos: Deben colocarse primero en una caja de acrílico transparente con orificios más pequeños que el diámetro de su cabeza para una ventilación adecuada; esta caja se colocará dentro de otra caja de madera o unicel, también con numerosos orificios que permitan la ventilación de la primera. La utilización de doble caja es muy importante para evitar que escapen y por la seguridad del personal que los maneja.

6.3.3. Serpientes no venenosas: Pueden estar contenidas en cajas de madera, unicel o acrílico con orificios para su ventilación. También pueden estar contenidas en sacos de tela resistente y permeable que permita el intercambio de aire, manteniendo dichos sacos en ambiente ligeramente húmedo. Los ejemplares se colocan de preferencia individualmente en sacos con la parte superior cerrada firmemente a manera de jareta con un cordón y para evitar la salida del ejemplar se debe hacer un segundo nudo bajo la jareta; posteriormente se colocan los sacos en cajas que contengan en el techo y paredes suficientes orificios. Las cajas deben estar bien cerradas.

6.3.4. Cocodrilos, lagartos, caimanes y gaviales: No deben movilizarse animales de diferentes especies en la misma caja. Los animales de gran tamaño deben viajar de manera individual; las cajas deben tener las dimensiones necesarias para que el animal permanezca ahí de manera estática, pero cómodo; evitándose al mismo tiempo que gire su cuerpo de modo circular o sobre su propio eje. La caja debe ser de madera o de otro material resistente, con orificios en paredes y tapa como se indica en el Apéndice E (Normativo). No se requiere alimentar animales adultos durante movilizaciones menores a 72 horas. El ayuno máximo para crías y animales en desarrollo deberá ser de 24 horas y se recomienda colocar cama de aserrín o viruta de madera en la caja.

6.4. Aves silvestres.

Para estas especies debe referirse al punto 4.1.6. de esta Norma.

- Punto 6.4.1.

a) Canoras, de ornato y en general aves de talla pequeña como canarios, pinzones, palomas, periquitos y loros pequeños. Se les confina en cajas o jaulas que pueden contar o no con perchas de un diámetro adecuado para que posen sus patas cómodamente durante el viaje. Las cajas deben contar con bebederos y comederos adecuados, dispuestos de tal forma que no puedan vaciarse mojando la jaula o a los animales.

La altura de la caja depende del tamaño de las aves, considerando siempre que tengan libertad para mantenerse erectas cuando existen perchas, como se indica en el Apéndice F (Normativo). Los barrotes de las jaulas o los orificios de las cajas cumplirán con las especificaciones del punto 6. inciso c). Cuando sea necesario se colocará en el piso una cama inocua y absorbente para que retenga la humedad de las deyecciones.

El transporte de palomas debe realizarse en cestas o jaulas adecuadas para ellas, ya sean individuales o en grupo y podrá llevarse a cabo por particulares o por agrupaciones dedicadas a las competencias o exhibiciones de estos animales.

Cuando se trate de agrupaciones, éstas realizarán el transporte de las palomas en vehículos con ventilación adecuada y se les proporcionará agua y alimento suficiente para el número de animales que transporta, cuando no se pueda realizar la suelta como el caso de las palomas mensajeras.

b) Aves de talla grande, psitácidos como loros, guacamayas, cacatúas u otras. Cuando las aves son muy grandes deben viajar en forma individual o cuando mucho 2 o 3 animales de la misma especie por caja. Las cajas o jaulas pueden contar o no con perchas de un diámetro adecuado para las patas de los animales, con la finalidad de que se posen cómodos durante el viaje, como se indica en el Apéndice F (Normativo). Los barrotes de las jaulas o los orificios de las cajas cumplirán con las especificaciones marcadas en el punto 6. inciso c). Cuando sea necesario se colocará en el piso una cama inocua y absorbente para que retenga la humedad de las deyecciones.

6.4.2. Aves de presa, como halcones, búhos, zopilotes, águilas, entre otras: Deben viajar en cajas que tengan o no percha con el diámetro correspondiente a la medida de la pata del ave para que pueda viajar cómodamente posada sobre ella, como se indica en el Apéndice F Normativo. Las cajas contarán con las especificaciones del punto 6. inciso c) y según la longitud del trayecto contarán o no con bebedero en su interior. El piso debe ser liso y de una textura que no lesione las patas de los animales. Cuando sea necesario se colocará en el piso una cama inocua y absorbente para que retenga la humedad de las deyecciones.

6.4.3. Aves palmípedas como gansos, pelícanos, patos, garzas y otras: Para movilizarlas durante periodos no mayores de 8 horas, se recomiendan pisos lisos sobre los que se posen los animales, y en el caso de movilizaciones prolongadas se recomiendan pisos de rejilla, en cuyas separaciones no puedan atorarse las patas de los animales y para que a través de esa rejilla drenen las excretas, las cuales deben caer en una charola, para poderlas limpiar sin molestar a los animales. Las paredes y techo deben tener numerosos orificios como se indica en el Apéndice G Normativo. Se les debe colocar comedero y recipientes para agua, los cuales estarán fijos a una de las paredes.

6.4.4. Aves grandes o corredoras como avestruz, emú, rhea, casuarios, entre otras: Deben movilizarse en cajas con muy buena ventilación, al igual que se menciona en incisos anteriores, y se recomienda que el interior esté oscuro para disminuir el estrés. El espacio por dentro debe ser suficiente para que el animal pueda moverse, no se deben colocar más de 2 aves por contenedor; o bien, pueden movilizarse en grupo, el piso será de un material suave y absorbente para evitar que se lastimen las patas, preferentemente no se utilizará material de cama ya que los animales pueden ingerirlo; si la movilización no es mayor de 24 horas, no se requiere darles alimento; si la movilización es más prolongada debe haber comedero y bebedero en el interior del contenedor para ofrecerles agua y comida. También pueden movilizarse en camiones para transporte de equinos, siempre y cuando tengan buena ventilación y el piso sea antiderrapante, como se indica en el Apéndice G (Normativo).

6.5. Mamíferos terrestres.

6.5.1. Primates no humanos: Deben movilizarse en cajas o jaulas con poca separación entre los barrotes, o bien, en cajas individuales de madera totalmente cerradas con orificios de acuerdo al punto 6. inciso c) de esta Norma, con abundante cama sin charola y con cerrojos seguros.

La caja debe tener una pequeña puerta o ventana como se indica en el Apéndice H (Normativo), por la que se pueda observar al animal y darle agua y alimento con la frecuencia que se requiera, en viajes mayores de 8 horas generalmente es necesario.

6.5.2. Carnívoros: Las cajas para movilizarlos deben ser lo suficientemente amplias para que los animales se puedan mover y darse vuelta cómodamente. En su diseño se debe considerar una doble puerta de preferencia y los implementos necesarios para cargar o mover la caja en forma segura, como se indica en el Apéndice H (Normativo). El material de construcción debe ser lo suficientemente resistente y seguro, de acuerdo con el tamaño del animal que se desea movilizar, para que resista los movimientos bruscos del mismo, así como la acción de colmillos o garras que puedan dañar esos materiales. Cuando viajen hasta 24 horas no requieren de alimento ni agua, excepto para animales en crecimiento o especies de tamaño pequeño que por lo menos requieren agua, pero si la movilización es de mayor duración siempre se les ofrecerá comida y agua.

6.5.3. Animales de circos y espectáculos: Para su movilización se debe cumplir con los requisitos señalados en esta Norma para cada una de las especies que corresponda.

Los osos entrenados pueden viajar en un contenedor resistente o en la caja del camión, siempre y cuando estén sujetos por el cuello con un collar, el cual se debe sujetar unido a una cadena que se debe fijar a una estructura interna firmemente, como se indica en el Apéndice J (Normativo).

6.5.4. Herbívoros pequeños y grandes como camélidos, cebras, cérvidos y antílopes, entre otros: El transporte puede ser individual o en grupo tomando en cuenta sexos y tamaño de los individuos, los contenedores deben ser resistentes, separados interiormente por divisiones, que ni los compriman ni les permitan moverse en grandes espacios, para evitar que se agrupen en las esquinas. El largo de la caja no debe exceder de 40 cm del largo del cuerpo del animal para sólo permitir que se coloque agua o alimento en caso necesario. Si se trata de cajas individuales se debe considerar el uso de puerta en ambos extremos. Debe colocarse abundante cama sobre el piso no derrapante en cualquiera de los casos, sin separaciones para evitar que los animales se atoren en ellas.

A los cérvidos se les puede o no cortar las astas antes de movilizarlos. No se deben transportar durante la etapa en que las astas están cubiertas por terciopelo (velvet). En el caso de los antílopes se les deben proteger los cuernos para evitar que se fracturen. La altura de la caja será suficiente para que tengan libertad de enderezar la cabeza en forma cómoda, como se indica en el Apéndice J (Normativo).

6.5.5. Jirafas: Los contenedores pueden estar fabricados sin techo para animales adultos ya que así pueden ver a su alrededor y se estresan menos, deben tener una anchura ligeramente mayor a la del cuerpo del animal y una altura que permita que sólo quede libre la cabeza y un tercio del cuello de la jirafa; para crías o animales jóvenes se utilizan cajas o contenedores preferentemente con techo; como se indica en el Apéndice L (Normativo). Debe colocarse abundante cama en el piso no derrapante. Las maniobras de movilización se deben hacer con delicadeza para evitar cualquier riesgo de fracturas en las extremidades o cuellos tan largos.

6.5.6. Rinocerontes, hipopótamos y elefantes: Para movilizar a estos animales se emplean cajas fuertemente construidas con materiales muy resistentes y preferentemente cerrados con puerta en ambos extremos, con piso antiderrapante y suficiente cama. Deben medir sólo un poco más del ancho del cuerpo de cada animal para evitar que puedan darse la vuelta adentro. La longitud de la caja solamente será cerca de medio metro más que el largo del cuerpo del animal para que en viajes largos se les pueda proporcionar alimento y agua. En el caso de la movilización de uno o varios elefantes a la vez, pueden viajar libres en la caja del contenedor siempre y cuando dos de sus patas, una delantera y la contraria trasera, se encuentren sujetas a una cadena sin lastimarlos, las cuales a su vez se fijen en argollas fuertemente sujetas al piso del vehículo como se indica en el Apéndice L Normativo. Cuando se transportan varios animales juntos, se debe considerar siempre la compatibilidad de carácter, talla y sexo.

Los animales muy jóvenes y de talla pequeña pueden viajar en grupo y sin sujeción.

7. Movilización de abejas productoras de miel (*Apis mellifera l*)

Las abejas productoras de miel son susceptibles de movilizarse en: colmenas pobladas para producción de miel, para polinización, núcleos de abejas, paquetes o jaulas con abejas a granel.

Se deben movilizar con suficiente ventilación y proporcionarles agua limpia diariamente, en especial en climas cálidos.

No se expondrán a productos tóxicos como pesticidas, substancias derivadas del petróleo y los gases del escape de los camiones de transporte de carga.

En el caso de las colmenas pobladas, se pueden transportar con tapas de viaje y piqueras cerradas, también con piqueras abiertas sin tapas de viaje, en transportes con cajas refrigeradas con ventilación restringida o en camiones abiertos, en el que el conjunto de colmenas se cubran con una malla resistente que les impida salir.

8. Sanciones

El incumplimiento a las disposiciones contenidas en la presente Norma, se sancionará conforme a lo establecido en la Ley Federal de Sanidad Animal y la Ley Federal sobre Metrología y Normalización.

9. Concordancia con normas internacionales

Esta Norma Oficial Mexicana no es equivalente con ninguna norma internacional.

10. Bibliografía

Animal and Plant Health Inspeccion Service (APHIS), USDA 9 CFR.CH.1 (1-1-92 edition).

Australian National Code of Practice for Welfare of Animals. Land Transport of Horses-1995. Standing Committee on Agriculture and Resource Management.

Arzave Barrera Alfonso. Miembro de la Asociación Mexicana de Médicos Veterinarios Especialistas en Equinos. Comunicación personal.

Commission of the European Communities. Directorate General for Agriculture VI B11.2.

Council of Europe, Committe of Ministers to Member States on the Transport of Horses-1987.

Editorial El Manual Moderno S.A de C.V. Manual de Producción Avícola 1993.

Grandin, T.; 1979. Understanding animal psychology facilitates handling livestock. Vet. Med. Small Anim. Clin. 74:697-706.

Grandin, T.; 1980a. The effect of stress on livestock and meat quality prior to and during slaughter. Int. J. Stud. Anim. Prob. 1:313-337.

Grandin, T.; 1982b. Understanding hog psychology simplifies handling. Vet. Med. Small Anim. Clin. 77:267-271.

Grandin, T.; Applied Animal Behaviour Science. 1990; 28:187-201. Animal Science Department, Colorado State University, Fort Collins, CO 80523, USA.

González Orijel Antonio. Confederación Nacional Ganadera. Comunicación personal.

Guideline for transport and preparation for shipment of live wild animals and plants. CITES.

López Coello Carlos. Departamento de Producción Animal; Aves. FMVZ-UNAM. Comunicación personal.

Nostrand van Reinhold. Commercial Chicken Production Manual ISBN 0-0442-31882-2.

Report of the Scientific Veterinary Comitee.; Animal Welfare Section. On the Transport of Farm Aminals. VI/3404/92-EN Brussels, 18 May 1992.

SAGAR.; Instituto Nacional de Investigaciones Forestales y Agropecuarias. División de Investigación Pecuaria. Recomendaciones para el acondicionamiento del ganado, su transportación y recepción. CENID Fisiología y Mejoramiento Animal-1996.

Stephens, D.B.; Perry, G.C.; Applied Animal Behaviour Science, 1990;28: 1-2, 41-55-44. Royal Veterinary College, Hawskhead Lane, North Mymms, Hatfield, AL9 7TA.UK.

11. Disposiciones transitorias

Las disposiciones contenidas en la presente Norma Oficial Mexicana entrará en vigor al día siguiente de su publicación en el **Diario Oficial de la Federación**, excepto los siguientes puntos que a continuación se indican:

11.1. Los puntos 4.3.10.; 4.4.2.; 4.4.3. inciso d) y 5.1.3. inciso a), entrarán en vigor un año después, contado a partir de la fecha de publicación en el **Diario Oficial de la Federación.**

11.2. Los puntos 4.3.7. inciso d); 4.6.1.; 5.3.4 y 5.4.3,: entrarán en vigor tres años después, contados a partir de la fecha de publicación en el **Diario Oficial de la Federación.**

Sufragio Efectivo. No Reelección.

México, D.F., a 24 de febrero de 1998.- Con fundamento en lo dispuesto por el artículo 65 del Reglamento Interior de esta Dependencia, el Director de Coordinación Jurídica, **Sergio Guerra Beltrán**.- Rúbrica.

APENDICE A (NORMATIVO)

DESTINATARIO	ETIQUETA		REMITENTE
Nombre:	Contenido:	(Venenoso)	Nombre:
Domicilio completo:	Nombre común:		Domicilio completo:
	Nombre científico:		
Tel.:	Número de especímenes:		Tel.:

Temperatura Requerida	Alimentación	Sedación	Documentación que se acompaña
Máx C	Instrucciones:	Producto:	Certificado de salud
			Permiso exportación
		Sí ( )	o importación
Mín C	(No alimentar)	No ( )	Otros

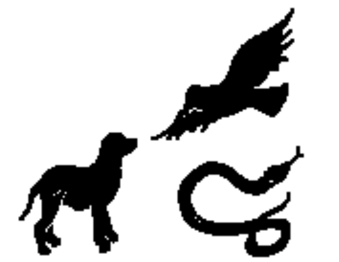

ANIMALES VIVOS MANEJESE CON CUIDADO

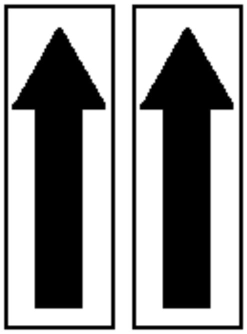

POSICION DE LA CAJA

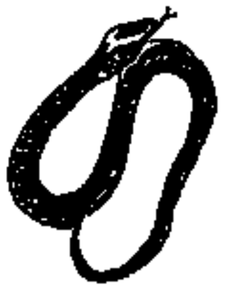

PELIGRO VENENOSO

APENDICE B (NORMATIVO) INVERTEBRADOS TERRESTRES

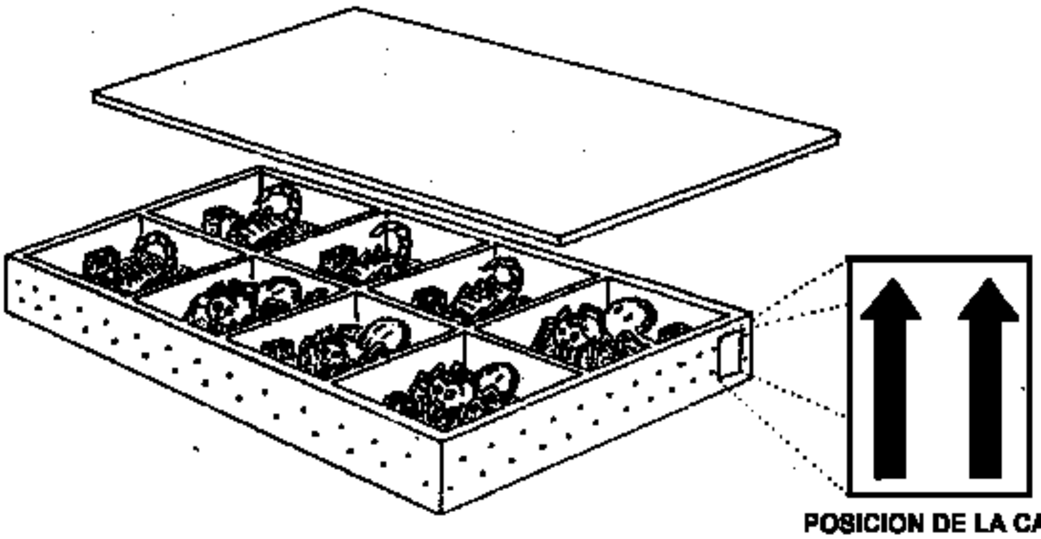

POSICION DE LA CAJA

APENDICE C (NORMATIVO) ANFIBIOS DE ABASTO

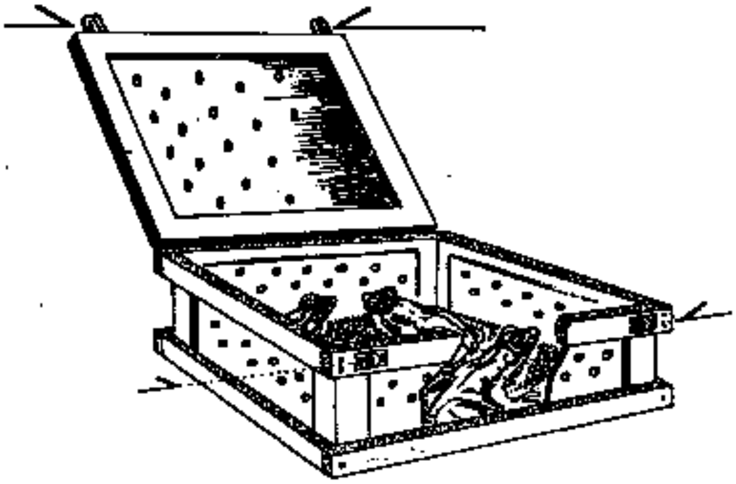

APENDICE D (NORMATIVO) TORTUGAS Y SAURIOS

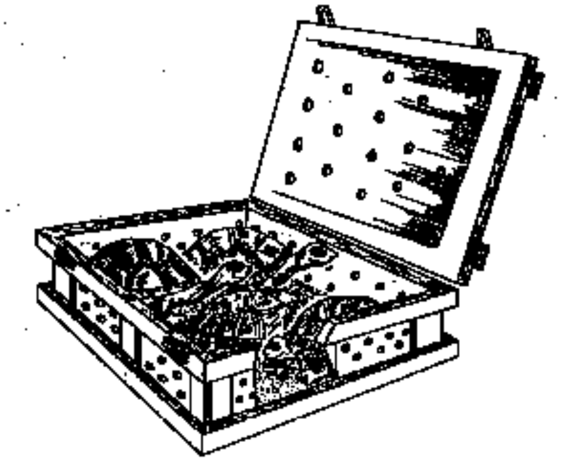

APENDICE E (NORMATIVO) COCODRILOS

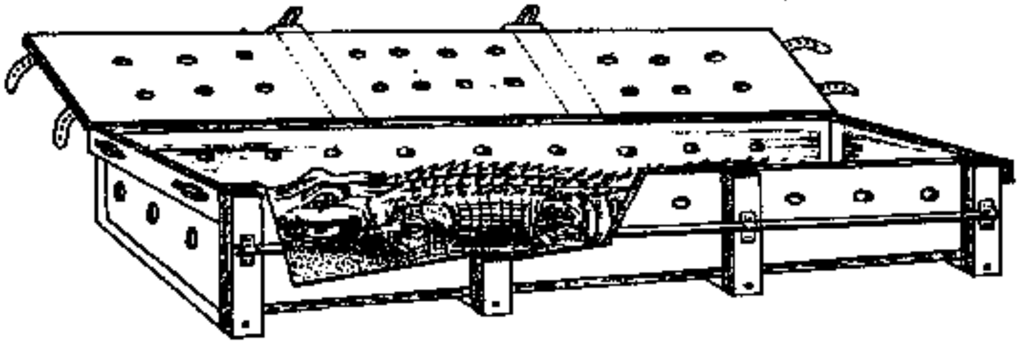

APENDICE F (NORMATIVO)

AVES PEQUEÑAS. CANORAS Y PALOMAS

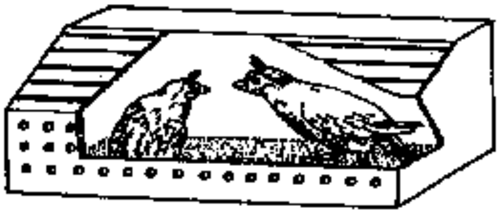

AVES CANORAS O DE ORNATO

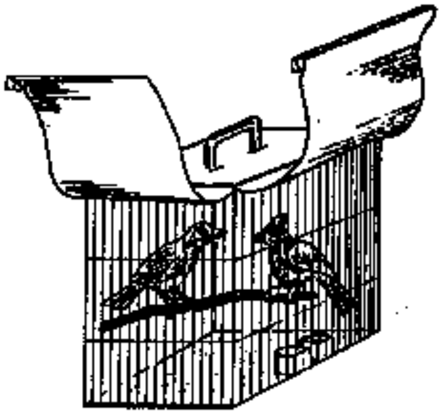

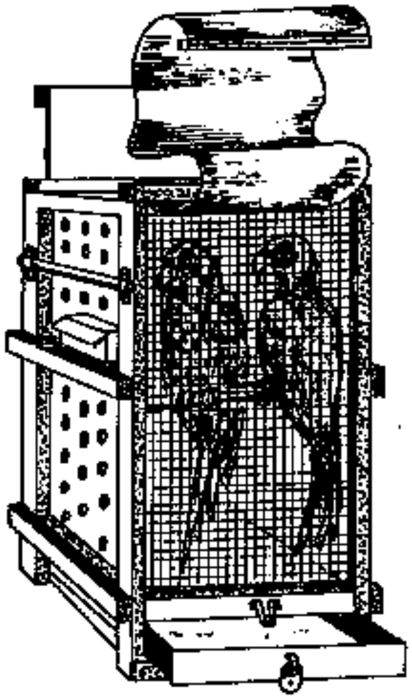

AVES PSITACIDAS

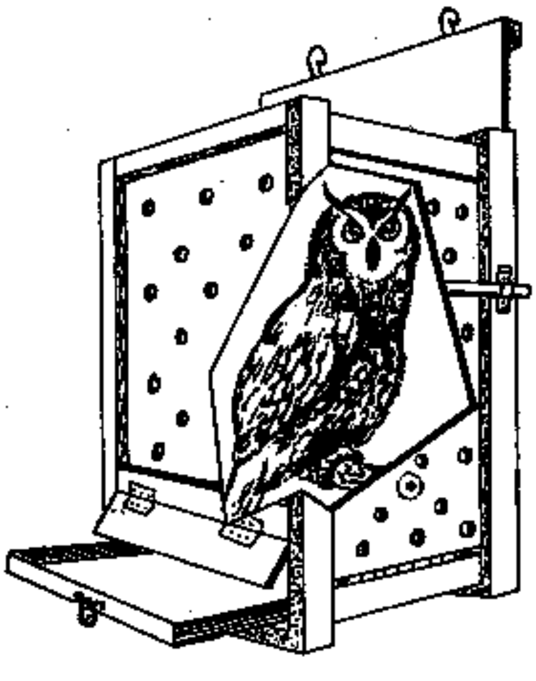

AVES DE PRESA

APENDICE G (NORMATIVO)

AVES PALMIPEDAS

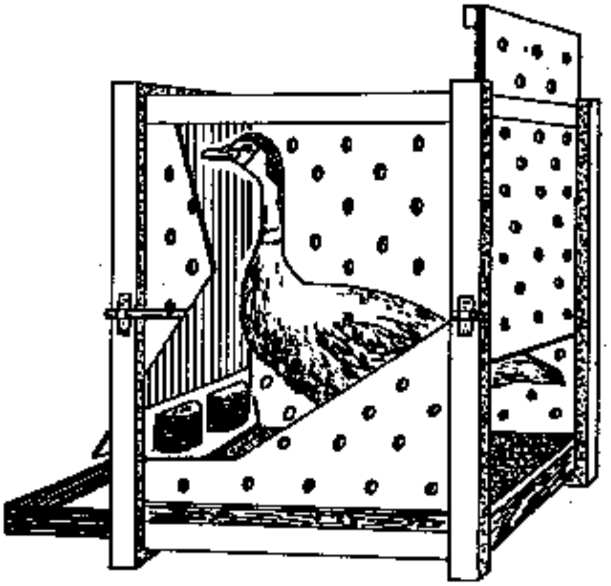

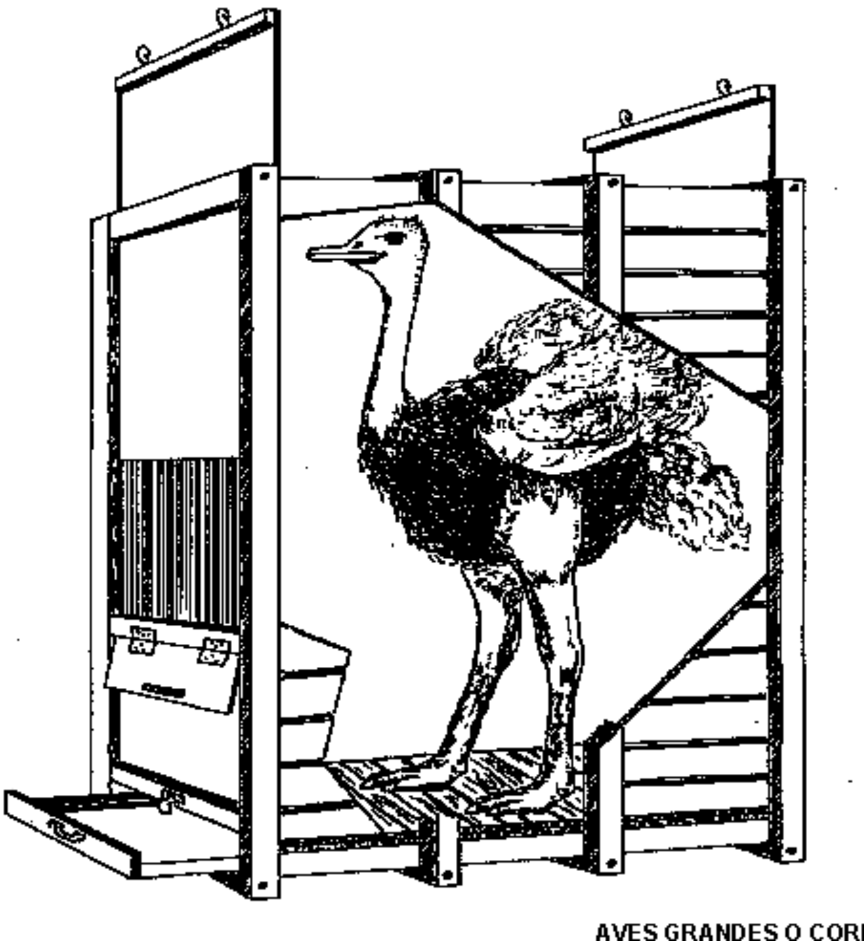

AVES GRANDES O CORREDORAS

APENDICE H (NORMATIVO)

PRIMATES NO HUMANOS

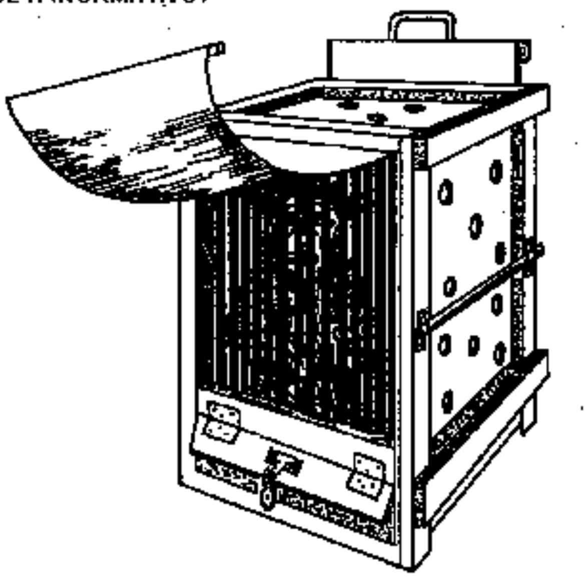

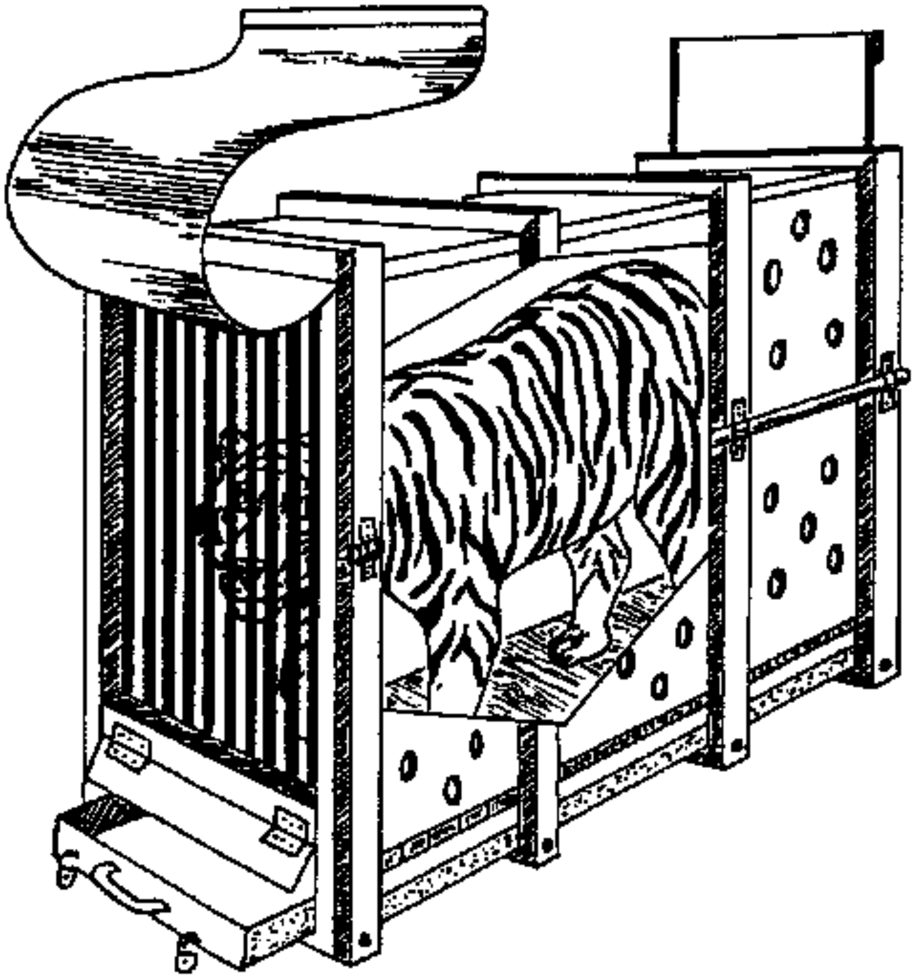

CARNIVOROS

APENDICE J (NORMATIVO) OSOS

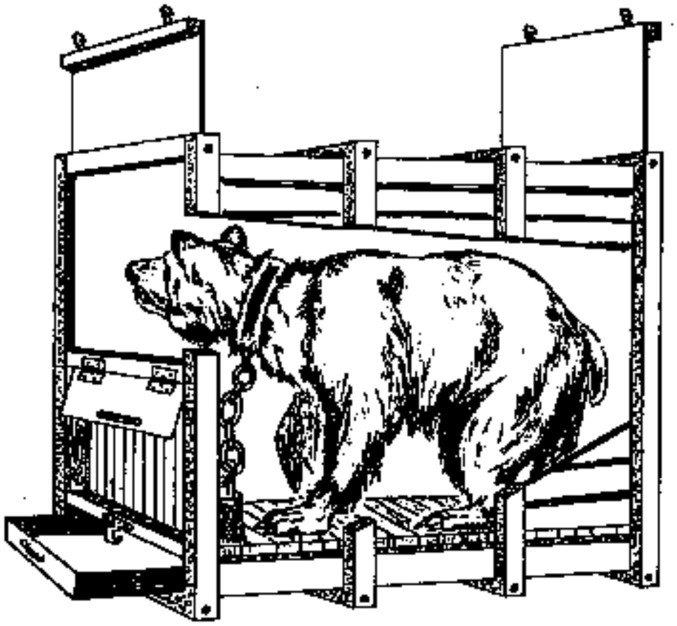

HERBIVOROS PEQUEÑOS Y GRANDES

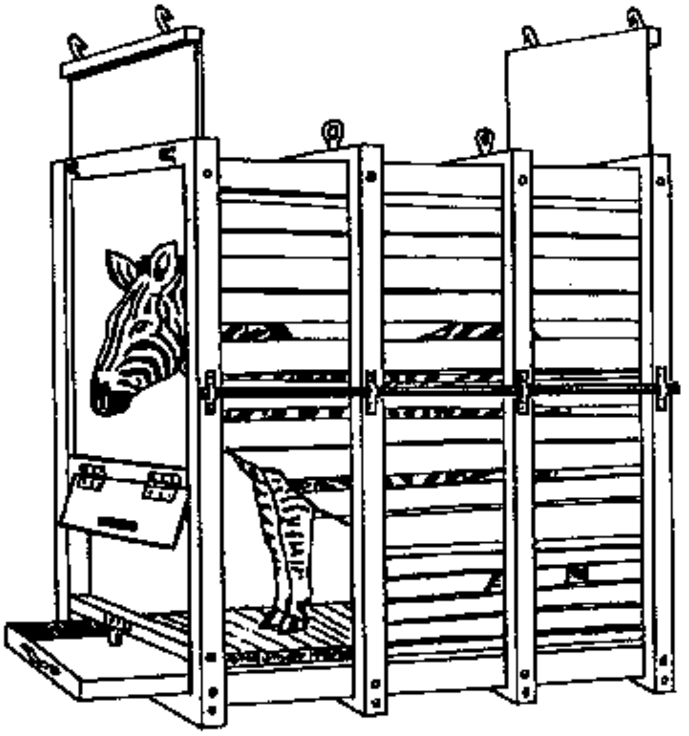

CEBRA

APENDICE L (NORMATIVO) JIRAFAS PEQUEÑAS

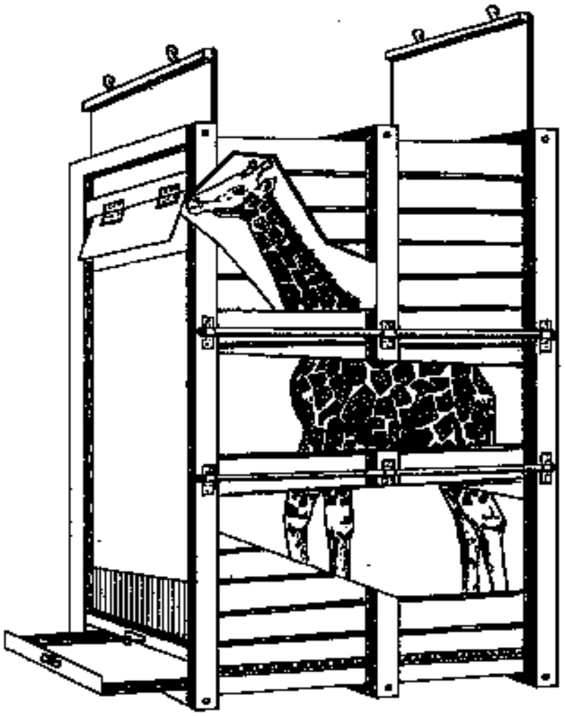

ELEFANTES

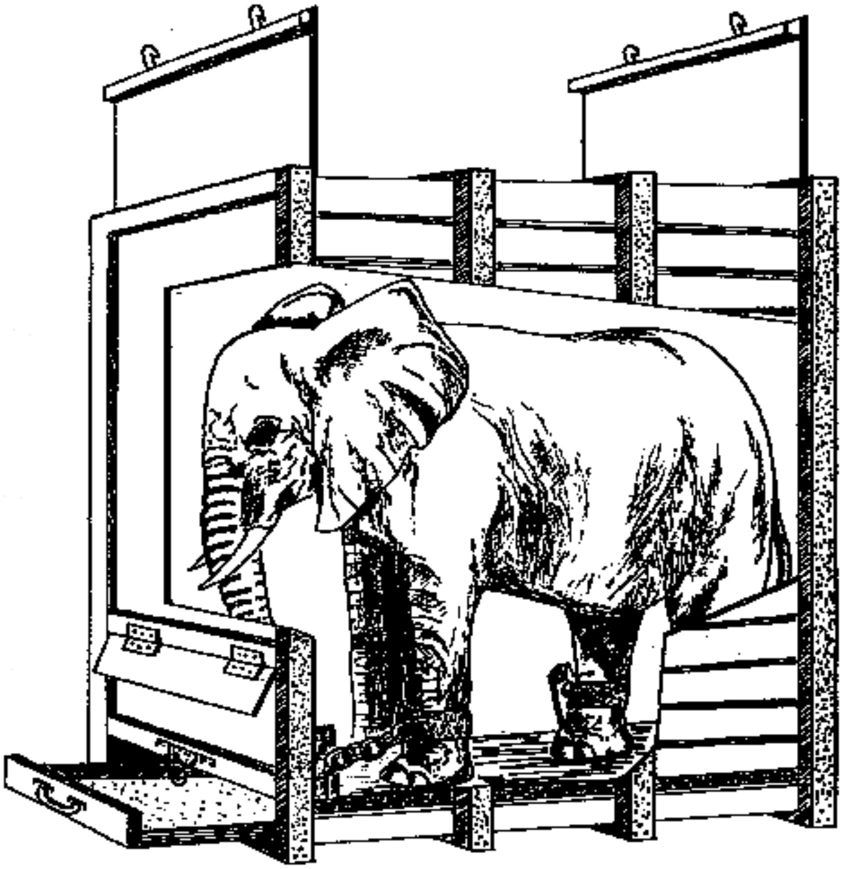

Supplement: S1 File — (PDF) [file pone.0118602.s001.pdf]
