# Supplementary material for: Black-Tailed Prairie Dogs, Cattle, and the Conservation of North America’s Arid Grasslands
Source: PLoS One. 2015 Mar 11;10(3):e0118602. doi: 10.1371/journal.pone.0118602 (PMC4356543; doi:10.1371/journal.pone.0118602)
Supplement: S2 File — (PDF) [file pone.0118602.s002.pdf]

PUBLICADA EN EL DIARIO OFICIAL DE LA FEDERACIÓN EL 22 DE AGOSTO DE 2001

NORMA Oficial Mexicana NOM-062-ZOO-1999, Especificaciones técnicas para la producción, cuidado y uso de los animales de laboratorio.

Al margen un sello con el Escudo Nacional, que dice: Estados Unidos Mexicanos.- Secretaría de Agricultura, Ganadería, Desarrollo Rural, Pesca y Alimentación.

NORMA OFICIAL MEXICANA NOM-062-ZOO-1999, ESPECIFICACIONES TECNICAS PARA LA PRODUCCION, CUIDADO Y USO DE LOS ANIMALES DE LABORATORIO.

LILIA ISABEL OCHOA MUÑOZ, Coordinadora General Jurídica de la Secretaría de Agricultura, Ganadería, Desarrollo Rural, Pesca y Alimentación, con fundamento en los artículos 35 fracción IV de la Ley Orgánica de la Administración Pública Federal; artículos 4o. fracción III, 12 fracción XIV, 17 y 18 fracción VI de la Ley Federal de Sanidad Animal; 38 fracción II, 40, 41, 43 y 47 fracción IV de la Ley Federal sobre Metrología y Normalización; 15 fracciones XXX y XXXI del Reglamento Interior de esta dependencia, y

CONSIDERANDO

Que es atribución de la Secretaría de Agricultura, Ganadería, Desarrollo Rural, Pesca y Alimentación formular, aplicar y, en el ámbito de su competencia, expedir las disposiciones y medidas zoosanitarias necesarias para verificar y certificar el cumplimiento de las mismas.

Que es función de la Secretaría de Agricultura, Ganadería, Desarrollo Rural, Pesca y Alimentación, fomentar la producción, cuidado y uso de los animales de laboratorio mediante la aplicación de técnicas tendientes a garantizar la producción, proteger la salud y favorecer el buen uso de los animales de laboratorio.

Que en la actualidad, la falta de planeación en la producción de animales de laboratorio, la carencia de criterios uniformes relacionados con las actividades encaminadas al cuidado, manejo y utilización de animales con fines de investigación científica, desarrollo tecnológico e innovación, pruebas de laboratorio y enseñanza, han provocado que el cuidado, el trato y la aplicación de técnicas experimentales practicadas en estos animales, sea ejercida en forma inadecuada, representando graves daños en el bienestar de los mismos.

Que para lograr resultados confiables en la investigación científica, la docencia biomédica y el control de calidad, así como utilizar el menor número de animales posible, es necesario contar con animales de laboratorio en condiciones óptimas.

Que cuando se utilizan para fines experimentales procedimientos cuestionables, inaceptables o contrarios a los principios de ética, éstos pueden causar graves daños en el bienestar de los animales.

Que el trato y la atención inadecuada relacionada con las maniobras para la movilización de los animales de laboratorio, contribuye a elevar los factores de estrés que los hacen susceptibles a contraer enfermedades.

Que en virtud de lo anterior y como consecuencia del proceso de globalización en el que México se encuentra inmerso, es necesario establecer criterios uniformes que permitan regular eficientemente la operación de las actividades relacionadas con la producción, cuidado, manejo y uso de los animales de laboratorio, a fin de favorecer el bienestar de éstos, protegiendo al mismo tiempo su salud.

Que para alcanzar los objetivos señalados en los párrafos anteriores, con fecha 6 de diciembre de 1999, se publicó en el **Diario Oficial de la Federación**, el Proyecto de Norma Oficial Mexicana NOM-062-ZOO-1999, Especificaciones técnicas para la producción, cuidado y uso de los animales de laboratorio, iniciando con ello el trámite a que se refiere la Ley Federal sobre Metrología y Normalización; razón por la cual, con fecha 18 de junio de 2001, se publicaron las respuestas a los comentarios recibidos con relación a dicho proyecto.

Que en virtud del resultado del procedimiento legal antes indicado, se modificaron los diversos puntos que resultaron procedentes y por lo cual, se expide la presente Norma Oficial Mexicana

NOM-062-ZOO-1999, Especificaciones técnicas para la producción, cuidado y uso de los animales de laboratorio.

INDICE

1.Objetivo y campo de aplicación
2.Referencias
3.Definiciones y abreviaturas
4.Disposiciones generales
4.1. Manifestaciones del tipo de bioterio
4.2. Responsable de los procesos de operación
4.3. Perfil del personal técnico involucrado en la producción, cuidado y uso de los animales de laboratorio
4.4. Obtención de animales
4.5. Certificado de salud y calidad
4.6. Identificación y registro
4.7. Alimento
5.Animales que comprende esta Norma
5.1. Roedores
5.2. Lagomorfos
5.3. Carnívoros
5.4. Primates no humanos
5.5. Porcinos
6.Instalaciones
6.1. Instalaciones interiores bajo techo
6.2. Instalaciones en exteriores
7.Movilización
7.1. Consignación de los animales
7.2. Confinamiento o encierro primario para transportación
7.3. Vehículo de transporte
7.4. Provisión de agua y alimento
7.5. Cuidados durante la transportación
7.6. Estaciones de carga
7.7. Manejo de los animales
8.Técnicas experimentales
8.1. Analgesia y anestesia
8.2. Administración de fluidos y sustancias
9.Eutanasia
9.1. Objetivo
9.2. Consideraciones generales
9.3. Consideraciones sobre el personal
9.4.Métodos recomendables
9.5.Métodos aceptables condicionalmente
9.6.Métodos inadmisibles
9.7. Prohibiciones
9.8. Cuadros sinópticos
10. Medidas de bioseguridad y salud ocupacional para el personal involucrado con la producción, cuidado y uso de los animales de laboratorio
10.1. Normas generales
10.2. Capacitación del personal
10.3. Instalaciones y equipamiento

10.4. Medidas generales de protección e higiene personal
10.5. Evaluación médica y medidas de medicina preventiva
10.6. Disposición final de productos biológicos, excretas y cadáveres
10.7. Supervisión
11. Verificación
12. Sanciones
13. Concordancia con normas internacionales
14. Bibliografía
15. Disposiciones transitorias
Apéndices normativos e informativos

1. Objetivo y campo de aplicación

1.1. La presente Norma es de observancia obligatoria en todo el territorio nacional y tiene por objeto establecer y uniformar las especificaciones técnicas para la producción, cuidado y uso de los animales de laboratorio que deben cumplir las personas físicas o morales relacionadas en todos los campos con este tipo de animales.

1.2. Esta Norma es aplicable a los bioterios y/o establecimientos que manejen los siguientes animales; roedores: rata, ratón, cobayo, hámster y jerbo; lagomorfos: conejo; carnívoros: perro y gato; primates: primates no humanos; porcinos.

1.3. La vigilancia de esta Norma corresponde a la Secretaría de Agricultura, Ganadería y Desarrollo Rural, así como a los Gobiernos de los Estados y del Distrito Federal, en el ámbito de sus respectivas atribuciones y circunscripciones territoriales, de conformidad con los acuerdos de coordinación respectivos.

1.4. La aplicación de las disposiciones contenidas en esta Norma compete a la Dirección General de Salud Animal, así como a las Delegaciones de la Secretaría de Agricultura, Ganadería y Desarrollo Rural en el ámbito de sus respectivas atribuciones y circunscripciones territoriales.

2. Referencias

Para la correcta aplicación de esta Norma se deben consultar las siguientes normas oficiales mexicanas:

2.1. NOM-003-ZOO-1994, Criterios para la operación de laboratorios de pruebas aprobados en materia zoosanitaria.

2.2. NOM-029-ZOO-1995, Características y especificaciones para las instalaciones y equipo de laboratorios de pruebas y/o análisis en materia zoosanitaria.

2.3. NOM-033-ZOO-1995, Sacrificio humanitario de los animales domésticos y silvestres.

2.4. NOM-046-ZOO-1995, Sistema Nacional de Vigilancia Epidemiológica.

2.5. NOM-051-ZOO-1995, Trato humanitario en la movilización de animales.

2.6. NOM-087-ECOL-1995, Que establece los requisitos para la separación, envasado, almacenamiento, recolección, transporte, tratamiento y disposición final de los residuos peligrosos biológico-infecciosos que se generan en establecimientos que presten atención médica.

2.7. NOM-008-SCFI-1993, Norma Oficial Mexicana. Sistema General de Unidades de Medida.

2.8. NOM-018-STPS-1993, Relativa a los requerimientos y características de los servicios de regaderas, vestidores y casilleros en los centros de trabajo.

2.9. NOM-028-STPS-1994, Seguridad-Código de colores para la identificación de fluidos conducidos en tuberías.

3. Definiciones y abreviaturas

Para efectos de la presente Norma, se entiende por:

3.1. Ad-libitum: A libre acceso.

3.2. Agentes biológicos: Cualquiera de los microorganismos de ciertas clasificaciones o cualquier sustancia tóxica derivada de organismos vivos que pueden producir muerte o enfermedad en el hombre, animales o plantas en desarrollo.

3.3. Agentes físicos: Objetos o estructuras inanimadas o estados de la materia capaces de producir cambios fisiológicos.
3.4. Agentes químicos: Sustancias que producen efectos letales, lesivos o irritantes.
3.5. Alergias: Extrema reactividad de los organismos vivos a exposiciones subsecuentes de ciertos antígenos derivados de diversas sustancias químicas o desechos animales.
3.6. Analgésico: Fármaco que disminuye o suprime el dolor.
3.7. Anestésico: Fármaco que causa la parcial o total ausencia de sensibilidad.
3.8. Animales axénicos: Aquellos que son libres de microorganismos demostrables.
3.9. Animal de laboratorio: Animal usado en la investigación científica, desarrollo tecnológico e innovación, pruebas de laboratorio y enseñanza.
3.10. Animales gnotobióticos: Aquellos que están completamente libres de agentes patógenos o que pueden hospedar uno o más microorganismos claramente identificados.
3.11. Animal neonato: Todo animal recién nacido hasta que se vale por sí mismo.
3.12. Animales SPF: Aquellos que están libres de patógenos específicos para la especie.
3.13. Asepsia: Métodos o procedimientos que impiden o evitan el acceso de gérmenes patógenos o infecciosos.
3.14. Bienestar: Estadio de satisfacción de las condiciones biológicas, ambientales y psicológicas que requiere un animal para desarrollarse, vivir sano y expresar su conducta normal como animal de laboratorio.
3.15. Bioseguridad: Conjunto de métodos, técnicas, aparatos e instalaciones destinados a salvaguardar la salud y la vida de las personas, los animales de laboratorio y proteger el medio ambiente.
3.16. Bioterio: Conjunto de instalaciones, muebles e inmuebles destinados al alojamiento y manutención de animales de laboratorio durante una o varias de las fases de su ciclo vital; esto es, nacimiento, desarrollo, reproducción y muerte.
3.17. Capacitación: Acción y efecto de enseñar o habilitar a las personas para el cuidado y la utilización correcta de los animales de laboratorio.
3.18. Carnívoro: Aquellos mamíferos euterios, terrestres, tetrápodos que pueden alimentarse de carne, carroña, y aún ser omnívoros.
3.19. Comité: Comité Interno para el Cuidado y Uso de los Animales de Laboratorio.
3.20. CONASAG: Comisión Nacional de Sanidad Agropecuaria.
3.21. Confinamiento o encierro primario: Estructura física que limita los movimientos de desplazamiento de los animales de laboratorio y define su hábitat inmediato bajo condiciones normales de bioterio.
3.22. Cuarentena: Periodo de aislamiento al que se someten los animales de laboratorio, en un lugar específico, con el fin de conocer su estado de salud.
3.23. Desinfección: Procedimiento destinado a destruir los agentes patógenos para los animales y el ser humano, que se aplica a los locales, vehículos, así como a los implementos que sean utilizados en los establecimientos. Se debe efectuar posterior a la limpieza.
3.24. Dirección: Dirección General de Salud Animal.
3.25. Eutanasia: Procedimiento humanitario empleado para terminar con la vida de los animales de laboratorio, sin producirles dolor, angustia o sufrimiento.
3.26. Estrés: Reacción de los organismos vivos a diversos estímulos adversos, internos o externos, que tienden a alterar el equilibrio psicológico y fisiológico de un animal, a través de su exposición a condiciones extremas.
3.27. Médico Veterinario certificado: Profesional con reconocimiento válido y vigente, otorgado por un consejo de certificación nacional a través de la evaluación objetiva de sus conocimientos, habilidades, destrezas, valores y actitudes en el área específica de la ciencia de los animales de laboratorio. Dicho consejo debe estar acreditado por la autoridad que corresponda.

3.28. Médico Veterinario responsable: Profesional aprobado por la Secretaría de Agricultura, Ganadería, Desarrollo Rural, Pesca y Alimentación como coadyuvante en las funciones de asistencia técnica y capacitación zoosanitaria de los productores.
3.29. Médico Veterinario Zootecnista: Profesional con cédula expedida por la Dirección General de Profesiones de la Secretaría de Educación Pública.
3.30. Salud ocupacional: Estado de salud, relacionada a la ocupación laboral del individuo.
3.31. Sedante: Agente depresor del sistema nervioso central capaz de abolir estados de irritabilidad o excitación en un animal.
3.32. Secretaría: La Secretaría de Agricultura, Ganadería, Desarrollo Rural, Pesca y Alimentación.
3.33. Tranquilizante: Fármaco capaz de abolir la ansiedad e inducir sedación.
3.34. Zoonosis: Denominación genérica de las enfermedades infecciosas de los animales que pueden ser transmitidas al hombre.

4. Disposiciones generales

4.1. Manifestación del tipo de bioterio.
4.1.1. Toda persona física o moral que aloje, produzca, utilice o distribuya animales de laboratorio con fines de investigación científica, desarrollo tecnológico e innovación, pruebas de laboratorio y enseñanza, debe dar aviso de inicio de funcionamiento a la Secretaría a través de la CONASAG, proporcionando su nombre y el domicilio del establecimiento correspondiente, así como la referencia de lo que maneje o elabore, dentro de los primeros 15 días naturales siguientes a la apertura del mismo. Para el caso de aquellos establecimientos que ya estén en operación, deben dar aviso 15 días naturales a partir de la fecha de entrada en vigor de la presente Norma.
4.1.2. Toda entidad con aviso de inicio de funcionamiento ante la Comisión Nacional de Sanidad Agropecuaria, debe entregar un informe anual de actividades, de acuerdo con el Apéndice A (Normativo), especificando con toda veracidad el tipo de instalaciones con que cuenta de acuerdo con la descripción de tipos de bioterio clasificados con base en esta Norma.
4.2. Responsables del cumplimiento de esta Norma en la institución.
4.2.1. Todos los bioterios independientemente de su tipo tienen que designar como personas encargadas del cumplimiento de la Norma a:
a) Un Médico Veterinario responsable, que estará adscrito tiempo completo o tiempo parcial dependiendo del tamaño y las necesidades del bioterio.
b) Un responsable administrativo que será el director o la persona que éste designe para estos fines.
La institución debe asegurar los servicios médicos veterinarios a cualquier hora del día y de la semana para garantizar la salud y bienestar de los animales.
4.2.2. Comité Interno para el Cuidado y Uso de los Animales de Laboratorio.
Cuando una institución se encuentre en la categoría b (uso en investigación científica, desarrollo tecnológico e innovación, pruebas de laboratorio y enseñanza) o bien en la clasificación c (mixtos), debe conformar un Comité Interno para el Cuidado y Uso de los Animales de Laboratorio de carácter institucional.
4.2.2.1. La responsabilidad de la creación del Comité Interno para el Cuidado y Uso de los Animales de Laboratorio recae sobre el director o titular respectivo de la institución involucrada.
4.2.2.2. Inclusión de sus Miembros. Los miembros del Comité deben incluir:
a) Un Médico Veterinario titulado con experiencia comprobable en la medicina y ciencia de los animales de laboratorio.
b) Un investigador de alta jerarquía de la propia institución con experiencia comprobable en el manejo de animales de laboratorio.
c) Otras personas de acuerdo con las necesidades propias de la institución.
4.2.2.3. Función del Comité Interno para el Cuidado y Uso de los Animales de Laboratorio. Su función principal es la de asegurar la existencia de un mecanismo institucional encargado de

revisar que el cuidado y uso de los animales de laboratorio con propósitos de investigación, pruebas y/o enseñanza, sea de manera apropiada y humanitaria.
Las funciones del Comité deben especificarse en un Manual de Organización y Procedimientos.
4.2.2.4. Atribuciones del Comité Interno para el Cuidado y Uso de los Animales de Laboratorio. Serán atribuciones del Comité las siguientes:
a) Debe reunirse regularmente y realizar un informe anual acerca del estado que guarda el cuidado y uso de los animales de laboratorio en su institución mismo que entregará tanto a las autoridades de la Secretaría como a las propias de la institución.
b) Verificar las normas y guías establecidas para el cuidado y uso de los animales de laboratorio según sus propias necesidades institucionales.
c) Evaluar y aprobar los protocolos de investigación científica, desarrollo tecnológico e innovación, pruebas y enseñanza, que impliquen el uso de animales de acuerdo con los lineamientos expuestos en el Apéndice B (Normativo).
d) Tener autoridad para detener procedimientos relacionados con el uso de los animales, si no cumple con el procedimiento aprobado por el Comité y someter a eutanasia a aquellos animales en los que el dolor/sufrimiento no puede ser aliviado.
e) Resolver situaciones imprevistas no consideradas en la presente Norma.
f) Otras funciones de acuerdo a las necesidades establecidas por la Secretaría.
Las atribuciones del Comité deben especificarse en un Manual de Organización y Procedimientos.
4.2.2.5. Esquema de Funcionamiento del Comité Interno para el Cuidado y Uso de los Animales de Laboratorio.
Ver imagen 22ago01.bmp
4.3. Perfil del personal técnico involucrado en la producción, cuidado y uso de los animales de laboratorio.
4.3.1. Personal técnico.
4.3.1.1. Auxiliar de técnico de bioterio.
Personal capacitado y preferentemente certificado para realizar los procesos de atención diaria de los animales de laboratorio (alimentación, limpieza, inmovilización física) con capacidad para comprender su responsabilidad en el equipo de investigación, así como los aspectos éticos y legales relacionados con el uso de los animales de laboratorio. Asimismo, debe ser capaz de identificar signos de enfermedad, conducta anormal, dolor y sufrimiento.
Efectúa actividades como: limpieza de los animales, limpieza y descontaminación de cuartos, equipo y materiales del bioterio, proporciona alimento, registra las condiciones ambientales, sujeta y sexa animales, identifica y anota datos básicos en los registros, entre otras.
4.3.1.2. Técnico de bioterio.
Personal capacitado y preferentemente certificado para realizar además de los procesos del auxiliar técnico, los relacionados con el cuidado, producción y manejo experimental básico de los animales de laboratorio. Cuenta con conocimientos de anatomía y fisiología de los animales de laboratorio, así como principios de anestesia.
Efectúa actividades de esterilización de equipo y materiales, inmoviliza, toma muestras y administra sustancias y tratamientos bajo indicación del médico veterinario o del investigador, registra los datos reproductivos de la colonia y revisa las actividades del auxiliar técnico.
4.3.1.3. Tecnólogo o supervisor técnico.
Personal capacitado y preferentemente certificado para realizar procesos descritos en las posiciones anteriores (auxiliar y técnico) y los procesos administrativos y de supervisión media relacionados con la producción, cuidado y uso de los animales de laboratorio. El Médico Veterinario responsable del bioterio supervisará dichas actividades. Debe poseer destreza para desarrollar conceptos, habilidades para el manejo de personal y habilidad técnica, pensamiento crítico y capacidad para identificar y resolver problemas.

Realiza actividades como: suministro de dietas, limpieza y uso de equipo especial, manejo de técnicas experimentales y tratamientos especiales, entrenamiento de auxiliares y técnicos, control de insumos, entrega de animales y equipo, entre otras.

4.3.2. Usuarios dentro de la institución.

Es una obligación de la institución donde se realiza investigación científica, desarrollo tecnológico e innovación, pruebas de laboratorio y enseñanza, con animales de laboratorio, asegurar que el personal profesional, técnico y estudiantil, esté capacitado para realizar los procedimientos con animales de laboratorio.

4.4. Obtención de animales.

Todos los animales deben adquirirse conforme a los preceptos jurídicos aplicables tanto a las instituciones como a los particulares que reciben o negocian con animales. Se debe promover invariablemente que todas las transacciones que involucren la adquisición de animales se conduzcan legalmente.

Los animales suministrados por los comerciantes o instituciones públicas con fines de investigación científica, desarrollo tecnológico e innovación, pruebas de laboratorio y enseñanza, deben acompañarse de información que describa el estatus microbiológico y genético de sus colonias o de animales individuales. Criterios similares se deben aplicar para aquellos transferidos de otras instituciones o incluso dentro de la misma institución.

Todos los traslados de animales incluyendo aquellos dentro de la misma institución deben ser planeados para minimizar el tiempo de traslado, el riesgo de zoonosis y antropozoonosis; además, los animales deben ser protegidos contra condiciones climáticas extremas o sofocación debido al transporte en cajas cerradas, en cajuelas de automóviles o similares. Se debe evitar el hacinamiento, brindar agua y alimento cuando esté indicado y protegerlos contra traumatismos. El estrés debe reducirse al mínimo atendiendo a las recomendaciones mencionadas.

Todo embarque de animales independientemente de su origen (centros de control canino, instituciones o comerciantes establecidos), debe ser revisado por el Médico Veterinario Zootecnista, con el fin de comprobar el cumplimiento de las especificaciones de adquisición y la ausencia de signos clínicos de enfermedad, debiéndose establecer a juicio profesional los procedimientos de cuarentena y estabilización de acuerdo a la especie y circunstancias. Es importante la estricta coordinación entre el personal que solicita y el que recibe los animales, así como el que está a cargo de su cuidado diario, para asegurar su recepción apropiada y la disponibilidad de instalaciones para su alojamiento.

4.5. Certificado de salud y calidad.

4.5.1. Todos los animales adquiridos por compra, donación o intercambio deben ir acompañados de documentos que establezcan las condiciones de salud y calidad en que se produjeron, criaron y mantuvieron hasta antes de su embarque o salida del lugar de origen.

4.5.2. Se debe llenar un formato que contenga la siguiente información:

a) Nombre, dirección y razón social del proveedor.

b) Número de expediente que se otorga por la notificación de aviso de funcionamiento.

c) Especie animal a la que se refiere el certificado.

d) Raza, cepa.

e) Cantidad total de animales.

f) Sexo, indicando la cantidad de cada uno.

g) Fecha de nacimiento, cuando se conozca.

h) Pruebas de laboratorio y/o gabinete, incluyendo fecha de la última realización para determinar el estado microbiológico cuando se requiera.

i) Nombre y firma del Médico Veterinario Zootecnista certificado en animales de laboratorio que avala el certificado.

4.5.3. Salud animal.

a) Todas las instituciones donde se alojen animales de laboratorio con fines de investigación científica, desarrollo tecnológico e innovación, pruebas y enseñanza, deberán implantar programas sanitarios para la prevención de enfermedades.
b) Las cajas y jaulas se mantendrán limpias, secas y en condiciones ambientales aceptables.
c) Todos los días se observarán los animales para detectar cambios de comportamiento, enfermedades, heridas o muerte.
d) El agua suministrada a los animales debe ser potable y a libre acceso.
4.6. Identificación y registro.
Toda operación de un bioterio debe contar con registros diversos para el adecuado control de sus poblaciones animales, ya sean colonias de producción o bien de animales bajo experimentación. Estos sistemas incluyen desde tarjetas de jaula individuales o colectivas, hasta hojas clínicas o impresos de computadora que auxilien al veterinario o investigador en dicha tarea. Ver cuadro número 1
Todos los métodos utilizados deben ser selectivos para cada especie animal o circunstancia, de aplicación rápida y de ser posible indoloros. De resultar invasivos, capaces de inducir molestias considerables al sujeto, deberán emplearse sustancias o fármacos sedantes o anestésicos a juicio veterinario.
Las marcas o diseños elegidos en cada caso deben ser acordes a la Norma, para su fácil identificación, en los programas de producción y uso de los animales de laboratorio.
Los métodos aceptables son los siguientes:
Las tarjetas.- Se colocan en las jaulas o cajas y los datos que en ella figuran corresponden a la identificación que llevan los animales que contienen. Se pueden asentar datos sobre la procedencia, método de reproducción, inoculaciones, cirugías a que han sido sometidos y el responsable del proyecto de investigación.
Marcas naturales.- Se consideran las características fenotípicas, siempre y cuando sean fácilmente detectables y sin posible confusión. Deben estar perfectamente identificadas en las fichas, mediante dibujos o señales particulares.
Colorantes o tinturas.- Sólo se recomiendan en casos de identificaciones temporales, en un tiempo no mayor de 20 días y usar colorantes que no sean tóxicos para los animales.
Perforaciones y muescas.- Estas se aplican en las orejas de ratas, ratones y porcinos principalmente, de acuerdo a un código preestablecido.
Aretes.- Se colocan en la(s) oreja(s) y pueden ser de plástico o bien de metal. En ellos se graban o insertan letras, números o una combinación de ambos para facilitar la identificación.
Tatuaje.- Es aplicable en diversas partes del cuerpo del animal de acuerdo con la especie.
Collares.- Lo más importante a tener en cuenta es que los dispositivos de ajuste sean cómodos y sobre todo que contengan los distintivos con los datos de identificación. Se utilizan preferentemente en perros, gatos y primates no humanos.
Transmisores subcutáneos.- Este sistema se puede aplicar a todas las especies señaladas en esta Norma.
CUADRO No. 1
IDENTIFICACION

ANIMAL	TIPO DE IDENTIFICACION	ZONA DE APLICACION
Rata y ratón	Colorantes<br>Perforaciones y muescas<br>Tatuaje	Pelaje y cola<br>Oreja(s)<br>Parte superior interna de la oreja<br>Cola
Jerbo y hámster	Tatuaje<br>Perforaciones y muescas	Parte superior interna de la oreja<br>Oreja(s)

Cuyo	Colorantes<br>Aretes<br>Marcas naturales	Pelaje<br>Oreja (lo más cerca de la base)<br>De acuerdo al fenotipo
Conejo	Tatuaje<br>Aretes metálicos<br>Collares	Parte distal interna de la oreja<br>Oreja (lo más cerca de la base)<br>Cuello
Perro y gato	Collares con medallas<br>Tatuajes	Cuello<br>Parte superior interna de la oreja<br>Cara interna de la pierna
Porcino	Tatuaje<br>Perforaciones y muescas<br>Aretes	Parte superior interna de la oreja<br>Oreja(s)<br>Oreja (lo más cerca de la base)
Primates no humanos	Tatuajes	En jóvenes parte interna del muslo<br>En adultos debajo de la clavícula o pecho
Todos los animales indicados	Transmisor subcutáneo	Región de la espalda.

4.7. Alimento.
El alimento para todas las especies debe cumplir con las siguientes características:
a) Debe estar libre de aditivos, drogas, hormonas, antibióticos, pesticidas y contaminantes.
b) Debe estar dentro de su periodo de caducidad.
c) Almacenado en bodegas o cuartos desinfectados, secos y ventilados, sobre tarimas o en contenedores**.**

5. Animales que comprende esta Norma

5.1. Roedores: Rata, ratón, cobayo, hámster y jerbo.

5.1.1. Confinamiento o encierro primario.

El equipo para alojar a los animales debe estar diseñado para facilitar el bienestar del animal, satisfacer las necesidades de la investigación y reducir o eliminar las variables experimentales, por lo cual el equipo para confinar al animal o encierro primario debe:
a) Proporcionar el espacio adecuado que permita movimientos y adopciones de las posturas normales de la especie.
b) Ser cerrado a prueba de escape y proteger al animal de amenazas externas.
c) Ser adecuado en ventilación y conforme a las necesidades biológicas de la especie.
d) Ser resistente al lavado y desinfección frecuente.
Los roedores deben mantenerse en algunos de los tres tipos de jaulas o cajas existentes:
a) Cajas con pisos y paredes continuas sólidas y con tapa removible de reja o perforadas, en los cobayos pueden ser sin tapa.
b) Jaulas enteramente hechas de malla de alambre.
c) Combinación de los dos tipos:
Las jaulas consideradas en el inciso a) se podrán utilizar para cualquier etapa de desarrollo y experimentación.
Las jaulas y cajas consideradas en los incisos b) y c) con pisos de malla de alambre sólo podrán utilizarse cuando las condiciones experimentales lo exijan y nunca para parición, destete o mantenimiento prolongado. Los hámsteres deben alojarse en cajas con piso sólido.

Las recomendaciones de espacio mínimo que se deben proporcionar a los animales se describen en el cuadro número 2.

CUADRO No. 2
ESPACIO MINIMO PARA ROEDORES DE LABORATORIO MANTENIDOS EN JAULA O CAJA

ANIMAL	PESO EN GRAMOS	AREA DEL PISO POR ANIMAL EN cm^2	ALTURA EN cm DEL PISO AL TECHO DE LA JAULA O CAJA
Rata	<100	110	18
	100-300	187	20
	300-400	258	20
	400-500	387	20
	>500	452	20
Ratón	<10	39	12
	10-15	52	12
	15-25	78	12
	>25	97	12
Hámster	<60	65	18
	60-80	84	18
	80-100	103	18
	>100	123	18
Cobayo	<350	387	18
	>350	652	18
Jerbo	<60	65	15
	Entre 60 y 80	85	15
	Entre 80 y 100	105	15
	>100	125	15

5.1.1.1. Los confinamientos o encierros primarios deben tener las siguientes características; aun cuando en condiciones experimentales pueden variar, previa autorización del Comité.

a) Satisfacer las necesidades fisiológicas (alimentación, defecación, micción u otros) y conductuales de los animales, permitiendo los movimientos normales y ajustes posturales característicos de la especie.

b) Cuando esté indicado, deberá favorecer la reproducción y la crianza.

c) Permitir las interacciones sociales entre los individuos de la especie, el establecimiento de jerarquías y las conductas de escape.

d) Brindar una ventilación e iluminación adecuadas.

e) Favorecer que los animales se mantengan limpios y secos.

f) Ser seguras, impidiendo el escape de los animales o el entrampamiento de sus extremidades.

g) Deben tener bordes y aristas redondeadas.

h) El diseño debe facilitar la limpieza y saneamiento rutinarios y también las faenas de cambio, llenado y suministro de agua y alimento.

i) Permitir la observación de los animales.

j) Los materiales para la construcción de las jaulas deben ser resistentes, durables e impermeables.

k) Deben mantenerse en buenas condiciones de uso.

l) Se recomienda alojar a los roedores en jaulas con piso sólido y material de lecho.

5.1.2. Salud animal.

a) Cada una de estas especies se alojará en cuartos separados de otras especies o de animales con diferente condición microbiológica. Cuando se cuente con sistemas de aislamiento, podrán alojarse en el mismo cuarto.
b) Los roedores deben estar libres de las enfermedades zoonóticas y parásitos externos, a menos que el procedimiento experimental aprobado por el Comité indique lo contrario. En este caso, el bioterio debe contar con las medidas sanitarias y de control que asegure el control absoluto de los animales en experimentación (escape, contacto con roedores silvestres, eliminación de cadáveres u otros), así como protección del personal en contacto con los animales, sus partes o sus desechos.
c) El personal del bioterio debe someterse al menos una vez al año a un examen de salud.
5.1.3. Manejo.
5.1.3.1. Alimentación y provisión de agua.
a) El alimento debe proporcionarse a libre acceso o en forma restringida dependiendo de las necesidades de la cepa y de los procedimientos experimentales.
El alimento debe ser palatable, de una fórmula nutricional constante y certificado en cuanto a su composición. En el cuadro número 3 se describe la composición general de un alimento para roedores de laboratorio en base seca para cubrir las necesidades de crecimiento, gestación, lactación y mantenimiento.
Se podrá proporcionar alimentos frescos a los animales, siempre y cuando el Comité lo apruebe.
b) El agua debe ser potable y suministrarse a libre acceso durante toda la vida del animal.
Los procedimientos que requieran de privación de líquidos deben justificarse plenamente ante el Comité.
En caso de que el procedimiento experimental lo justifique y que sea aprobado por el Comité, se podrá hacer las modificaciones pertinentes a la dieta y forma de suministrar los alimentos.

CUADRO No. 3
COMPOSICION BROMATOLOGICA REQUERIDA PARA UN ALIMENTO DE ROEDORES DE LABORATORIO

ANIMAL	PROTEINA CRUDA %	GRASA CRUDA %	FIBRA CRUDA %	CENIZAS %	CONSUMO DIARIO DE ALIMENTO	CONSUMO DIARIO DE AGUA	VITAMINA C
Cobayo	17-25	4-11	12-16	7-9	25-30 g	12-15 ml/100 g	0.25-1 mg/g
Jerbo	15-24	4-11	4-6	6-8	10-15 g	3-4 ml	-
Hámster	16-24	4-11	3-6	5-8	7-18 g	8-12 ml	-
Rata	12-24	4-11	3-6	6-8	10-20 g	20-45 ml	-
Ratón	17-24	4-11	3-6	5-7	3-6 g	3-7 ml	-

5.1.4. Cama y nido.
Los roedores alojados en cajas con piso sólido deben tener el material de cama suficiente que garantice la absorción de su orina, excremento y desperdicio de agua, favorecer su aislamiento térmico y construcción de nido.
Los materiales de cama deben seleccionarse por su suavidad, capacidad de absorción, laxitud, ausencia de polvo y fragmentación, así como por la constancia de su calidad, neutralidad química, inercia nutricional y carencia de palatabilidad.
5.1.5. Agrupamiento de animales.

Con excepción de las indicaciones justificadas ante el Comité, los roedores deben alojarse en grupos, cuyo tamaño estará limitado por las especificaciones de espacio del cuadro número 2. En ratones y cobayos no deben alojarse más de un macho con una hembra o varias hembras en reproducción.

5.1.6. Manipulación e inmovilización.

Las técnicas de sujeción, manipulación e inmovilización que se realicen en el bioterio deben estar acordes con los principios humanitarios internacionales aceptados, aprobados por el Comité y supervisadas por el Médico Veterinario responsable.

5.2. Lagomorfos.

5.2.1. Conejos.

5.2.1.1. Confinamiento o encierro primario.

Los conejos de laboratorio requieren de jaulas con las siguientes características:

a) El material de las jaulas debe ser resistente, seguro y a prueba de escape. Las jaulas deben de permitir un fácil acceso al alimento y agua. Se recomienda como material: jaula con rejilla de acero inoxidable, con piso de malla de alambre con 1.5 cm de separación.

b) Las jaulas deben tener suficiente amplitud y altura para permitir a los conejos movimientos y posturas naturales. Los requerimientos mínimos de espacio se mencionan en el cuadro número 4.

5.2.1.2. Salud animal.

Los conejos deben estar libres de enfermedades transmisibles al hombre.

Los animales de nuevo ingreso deberán ser sometidos invariablemente a un periodo cuarentenario determinado a criterio del Médico Veterinario.

Los animales con signos clínicos evidentes de enfermedad, serán objeto de diagnóstico, aplicación del tratamiento correspondiente o retirados de la colonia a juicio del Veterinario.

Se debe realizar la inmediata remoción de los animales muertos.

No deben realizarse estudios post mortem en las áreas de los animales.

No se permitirá el reingreso de animales a las áreas de producción del bioterio.

5.2.1.3. Manejo.

5.2.1.3.1. Alimento.

Debe cubrir los requerimientos mínimos nutricionales para las etapas de crecimiento, gestación y lactación:

Proteína cruda	No menos del 16.0%
Grasa cruda	No menos del 2.5%
Fibra cruda	No menos del 14%
Cenizas	No más del 8.0%

5.2.1.3.2. Provisión de agua.

El agua de bebida debe ser potable. Debe administrarse a libre acceso excepto en los casos en que el protocolo experimental indique lo contrario.

5.2.1.3.3. Charolas colectoras de excretas.

a) Cuando el tipo de jaula requiera de charolas, éstas deben tener material absorbente y que cubra toda el área.

b) El cambio de charolas, su lavado y desinfección debe realizarse 2 veces por semana como mínimo.

c) El lavado y desinfección de las jaulas debe realizarse una vez por semana como mínimo o con mayor frecuencia de ser necesario para promover una higiene adecuada y evitar condiciones nocivas para los animales.

d) Los desechos generados por los animales, deben ser removidos y eliminados en condiciones de máxima higiene y seguridad.

e) A las hembras próximas al parto se les debe proporcionar un nido manufacturado de diversos materiales de tipo metálico, plástico u otros que promuevan la higiene. Estos deben

variar en dimensiones de acuerdo al tamaño de la raza. Para las razas medianas como la Nueva Zelanda los nidos tendrán de 45-50 cm de largo; 28-32 cm de ancho y 25-30 cm de altura, esta variación está dada acorde al tamaño de la hembra en gestación y al número de crías por parto. Los nidos deben estar cerrados parcialmente en el techo y completamente cerrados por sus costados.

5.2.1.3.4. Requerimiento mínimo de espacio para conejos.

CUADRO No. 4

REQUERIMIENTO MINIMO DE ESPACIO PARA CONEJOS

ANIMAL	PESO (Kilogramos)	AREA DEL PISO (m2)	ALTURA DEL PISO AL TECHO DE LA JAULA (cm)
Conejo	< 2	0.14	36
	Hasta 4	0.28	36
	Hasta 5.4	0.37	36
	>5.4	0.46	36

5.2.1.3.5. Manipulación e inmovilización.

a) La manipulación y sujeción del conejo deben ser llevadas a cabo con seguridad, firmeza y gentileza. Estas pueden realizarse de diferentes formas dependiendo de la distancia a transportar, talla y peso del animal y de la finalidad del manejo. La más usual consiste en colocar la mano debajo de los miembros posteriores y soportando el peso del animal, con la otra mano se debe sujetar la piel a nivel del cuello para mantener firme al animal.

b) Las técnicas de manipulación, sujeción e inmovilización deben ser realizadas por personal con experiencia o capacitado y/o bajo la supervisión del Médico Veterinario responsable.

5.3. Carnívoros.

5.3.1. Perros y gatos.

5.3.1.2. Confinamiento o encierro primario.

Para el alojamiento de los perros y gatos que se utilizan en investigación científica, desarrollo tecnológico e innovación, pruebas de laboratorio y enseñanza, debe contarse con jaulas, ya sea individuales o colectivas, las cuales deben tener espacio suficiente de acuerdo a las especificaciones señaladas en el siguiente cuadro. Las jaulas individuales deben contar con lugar para bebedero, comedero y tener condiciones adecuadas para limpieza y desinfección.

CUADRO No. 5

ESPACIO MINIMO RECOMENDADO PARA GATOS Y PERROS

Animales	Peso corporal Kg	Area de piso/animal m^2	Altura* cm
**Gatos**	**<4**	**0.28**	**61**
	**>4**	**0.37**	**61**
**Perros****	**<15**	**0.74**	**61**
	**Hasta 30**	**1.11**	**61**
	**>30**	**2.23**	**61**

NOTA: si requiere convertir kilogramos a libras, multiplique por 2.2

- Se considera desde el piso hasta el techo de la jaula. Para el caso de los perros, la altura correcta debe ser aquella que permita que su ocupante se ponga de pié cómodamente.

** Estas recomendaciones pueden requerir modificaciones de acuerdo al volumen corporal de los individuos, sobre todo los que están en el límite superior de cada rango de peso y que requieran de más espacio.

5.3.1.3. Salud animal.

Todos los perros y gatos que se utilicen en investigación científica, desarrollo tecnológico e innovación, pruebas de laboratorio y enseñanza, deben ser obtenidos de proveedores confiables a juicio del Comité. Los animales, al momento del arribo a las instalaciones, deben ser sometidos a un examen general realizado por un Médico Veterinario Zootecnista y ser tratados si lo requieren.

Todo animal antes de ser sometido a cualquier procedimiento debe ser bañado, vacunado contra la rabia y pasar un periodo de cuarentena que no podrá ser menor de 15 días, tiempo durante el cual se debe observar diariamente al animal para detectar signos de enfermedad, comportamientos anormales y heridas. Tanto las áreas de cuarentena, las jaulas individuales, las áreas para ejercicio y demás lugares donde se alberguen los animales deben mantenerse siempre limpias; las áreas de cuarentena deben desinfectarse después de cada utilización.

Sólo se aceptarán animales adultos y no podrán utilizarse hembras gestantes y cachorros a menos que la línea de investigación así lo requiera y para esto debe contarse con la recomendación aprobatoria del Comité.

5.3.1.4. Manejo.

5.3.1.4.1. Alimentación.

Los animales deben alimentarse con productos que cubran las necesidades nutricionales de la especie, edad y peso. En el caso de perros, la alimentación de cachorros debe realizarse desde el destete hasta los 9 meses 2 o 3 veces al día, y posteriormente una vez al día. Los alimentos deben estar acordes con la edad del animal, estado fisiológico del mismo y línea de trabajo o investigación que se esté realizando con ellos. Cuando el procedimiento experimental requiera de ayuno, éste no podrá exceder de 24 horas. En el caso de perros y gatos adultos la alimentación será ad-libitum.

5.3.1.4.2. Provisión de agua.

El agua que se suministre a los perros y gatos debe ser potable y suministrarse ad-libitum. Cuando el procedimiento requiera de restricciones, ésta no excederá de 12 horas excepto cuando el Comité así lo apruebe.

5.3.1.4.3. Agrupamiento de animales.

El procedimiento de agrupamiento consiste en alojar animales compatibles juntos. Nunca se debe hacinar a los animales, pues esto conduce a peleas. Se deben separar a los machos de las hembras para prevenir cruzamientos indeseados y peleas.

Los gatos pueden mantenerse en colonias de 4 a 6 animales. Los grupos deben formarse de acuerdo con las necesidades específicas de la investigación, cuidando siempre que sean grupos compatibles.

5.3.1.4.4. Manipulación e inmovilización.

Las técnicas de sujeción, manipulación e inmovilización que se realicen con los perros y gatos, deben estar aprobadas por el Comité y supervisados por el Médico Veterinario responsable.

5.4. Primates no humanos.

Los primates no humanos son los animales filogenéticamente más cercanos al hombre, lo cual los coloca en una situación ambivalente tanto en su uso con fines de investigación como en el trato y manejo que se les da.

5.4.1. Características generales.

Los primates no humanos requieren alojamiento especial y un buen conocimiento de su cuidado. Las medidas para proteger su estabilidad psicológica deben tener la misma prioridad que aquellas concernientes a su salud física.

El alojamiento, dieta, cuarentena, periodo de adaptación y otras prácticas deben adaptarse a la especie.

Estos animales son individuos que requieren de ambientes sociales y físicos enriquecidos que propicien su bienestar.

5.4.2. Uso en la investigación.

En México, los primates no humanos son utilizados principalmente para la producción de biológicos, en estudios de farmacología, toxicología, enfermedades infecciosas, neuroanatomía, neurofisiología, metabolismo, comportamiento y fisiología de la reproducción.

5.4.3. Adquisición.

Todos los primates no humanos deben ser obtenidos para los lugares donde son legalmente requeridos, de una manera lícita y el proveedor o criador debe contar con licencia. La importación de primates debe cumplir con los requisitos que establezca la Secretaría, sin menoscabo de las atribuciones que les corresponda a otras dependencias.

Cualquier animal importado debe haber sido tratado contra parásitos intestinales, enfermedades relacionadas con el estrés y realizado la prueba de la tuberculina. Deben estar libres de tuberculosis, salmonelosis y shigelosis.

El certificado de salud de cada primate recibido del proveedor debe tener una descripción clara del individuo de manera que pueda ser identificado inequívocamente, también debe tener registrados todos los medicamentos y vacunas aplicadas, así como el resultado de cualquier prueba que se le haya aplicado y las fechas de cuándo fueron realizadas. Deben tener la fecha de llegada del animal del país de origen, la fecha de nacimiento o de llegada al lugar en caso de provenir de algún criadero, también debe tener registrada la prueba de tuberculina certificada por el médico veterinario, avalando que ha sido realizada dentro de los 10 días anteriores a la compra y especificando también el método, tipo y concentración de tuberculina utilizados.

Los animales muertos deben ser acomodados individualmente en bolsas de plástico, bien cerradas e identificadas, y refrigerados hasta que sea realizada la necropsia por un patólogo o por personal calificado; en caso de no existir condiciones de bioseguridad adecuadas, los animales deben ser incinerados a la brevedad posible.

5.4.4. Cuarentena y periodo de adaptación.

La nueva remesa de primates debe ser recibida por personal con experiencia y familiarizado con los primates. Deben ser acomodados inmediatamente en un área de aislamiento previamente preparada y localizada tan distante como sea posible de la colonia. Los animales deben ser propiamente identificados y sus documentos deben ser revisados al momento de ser enjaulados. Los animales muertos durante este periodo deben ser tratados de la misma forma que se menciona en el cuarto párrafo del punto 5.4.3.

La cuarentena constituye un periodo de observación necesario para excluir la posibilidad de que un primate no humano, aparentemente sano, pueda albergar un agente biológico causante de enfermedad que se encuentre en periodo de incubación y que sea potencialmente peligroso para la salud humana y animal. Además, durante esta fase el animal se aclimata a su nuevo ambiente y alojamiento, clima, dieta y otros factores del bioterio.

Todo primate no humano debe someterse a un periodo de cuarentena, el cual en monos silvestres debe ser de un mínimo de 90 días. Esta medida es aplicable para todos los primates, excepto para los adquiridos de importadores o criaderos comerciales, en donde este periodo crítico puede ser reducido de acuerdo con el criterio veterinario. Bajo este esquema, cada sujeto debe ser individualmente monitoreado diariamente para determinar su ingesta de alimento y agua, así como su condición clínica. Además, cada primate no humano debe ser objeto de los análisis clínicos y tratamientos médicos antiparasitarios, antimicrobianos, u otros que sean necesarios a juicio veterinario, enfatizando que a su ingreso, independientemente de su origen, cada animal debe ser sometido al siguiente procedimiento estándar para prevención y detección de tuberculosis:

Cada animal, a su arribo, debe ser examinado por un Médico Veterinario, debiendo prestarse atención a la detección de nódulos linfáticos inflamados, mostrando secreciones (nódulos

axilares e inguinales), o bien a la palpación de masas abdominales inesperadas o signos de caquexia.
Cada animal debe ser tuberculinizado mediante la administración intradérmica de 0.1 ml de una suspensión de 1500 o más Unidades de Tuberculina de la “Vieja Tuberculina de Mamíferos” a intervalos de 2 semanas, en forma alterna en cada párpado, hasta que todos los animales del mismo grupo alcancen 3 pruebas negativas consecutivas. Cada animal debe ser firmemente inmovilizado o bien sedado mediante el fármaco de elección.
a) La lectura para observar la respuesta de la prueba debe realizarse a las 24, 48 y 72 horas. Las reacciones positivas pueden variar desde un mínimo enrojecimiento con ligero edema, hasta una notable inflamación del párpado con descarga purulenta. Asimismo, una reacción severa puede inducir hemorragia y necrosis en el sitio de inyección. De existir reacciones interpalpebrales difíciles de interpretar, la prueba debe repetirse, a las dos semanas, en la piel del abdomen en donde las reacciones pueden ser más fácilmente observadas y palpadas.
b) De existir un reactor positivo a la tuberculina, el animal debe ser sometido a eutanasia y debe realizarse una necropsia confirmatoria mediante el aislamiento del bacilo tuberculoso, por medio de los métodos actuales de diagnóstico.
c) Los animales con reacciones cuestionables deberán ser objeto de aislamiento, seguido de una radiografía de tórax y tuberculinizados a las dos semanas. Si los resultados son nuevamente dudosos se recomienda someterlos a eutanasia y el desarrollo de una necropsia y estudios confirmatorios de tuberculosis.
d) No es aceptable la retención de primates reactores positivos a la prueba de tuberculosis, a excepción de circunstancias extraordinarias que así lo justifiquen. En todo evento el animal debe ser aislado y alojado en un área especialmente equipada para trabajar con agentes infectocontagiosos, al tiempo que tanto los investigadores como el personal de apoyo deberán estar informados y acatar las medidas de bioseguridad que a juicio profesional procedan.
e) Finalmente, cada animal deberá ser tuberculinizado dentro de las dos semanas previas a su ingreso a una colonia de mantenimiento permanente, una vez confirmada la reacción negativa.
5.4.4.1. Normas de cuidado durante la cuarentena.
a) El área de cuarentena deberá estar debidamente equipada con utensilios básicos de limpieza, redes, jaulas, equipo de tatuaje y rasuradoras para uso exclusivo en este sector. Todo el equipo empleado debe ser lavado y desinfectado diario, y el cuarto completo, con todos sus contenidos, debe ser lavado y desinfectado exhaustivamente antes de la introducción de un nuevo grupo.
b) El personal asignado al área de cuarentena debe estar debidamente enterrado de los procedimientos necesarios para prevenir la transmisión de enfermedades entre los animales y de éstos al hombre. Asimismo, debe vestir uniforme de color distintivo único para este sector y artículos de protección como gorros, cubrebocas, guantes, etc. No podrá visitar o atender otra área de animales durante su jornada; si ello fuera preciso, deberá trabajar inicialmente en las colonias permanentes para continuar su rutina en el área de cuarentena.
c) El personal deberá cambiarse de ropa en el momento de ingresar a otra sección de la colonia de primates.
d) Se deberá llevar una historia clínica de cuarentena por individuo y por todo el lote, registrándose todos los eventos sucedidos en este periodo.
e) Cuando un animal del lote enferme o muera, se debe iniciar nuevamente el periodo de cuarentena que, en este caso, no podrá ser menor a 90 días.
f) Una vez concluida la cuarentena, esto debe ser oficialmente informado a las autoridades zoosanitarias.
g) Deberá mantenerse estricto aislamiento en la sección de cuarentena del bioterio, no permitiéndose la entrada de visitas ni de personal no autorizado.
5.4.5. Identificación.

Se utiliza principalmente la identificación permanente por medio del tatuaje y otros similares y la descripción contenida en su registro de llegada.

5.4.6. Alojamiento.

Nunca deben ser alojados juntos animales que pertenezcan a diferentes especies.

5.4.6.1. Requerimientos de espacio.

Aunque cada una de las especies tiene requerimientos particulares, en general, cada individuo necesita de un espacio de entre 0.063 y 0.167 m3/kg, cuando se encuentran en jaulas individuales; el alojamiento en grupo requiere de cuando menos 0.224 m3/kg, por individuo. Se debe tener especial cuidado con las especies cuyo medio de locomoción es braquial, con aquellas que se cuelgan o columpian o con colas prensiles; en estos casos se debe asegurar una altura que permita al animal estar colgado sin que sus pies toquen el suelo cuando estén completamente extendidos.

5.4.6.2. Alojamiento en cuartos.

Debido a que los primates no humanos son particularmente susceptibles a algunas enfermedades, éstos deben ser alojados en áreas o cuartos tan distantes como sea posible de los cuartos de otros animales y mantenerles separados por especie para evitar la transmisión de enfermedades. Debe asegurarse que siempre haya dos puertas entre el cuarto del mono y el corredor del edificio. Deben mantenerse cerrados, con señales designando los cuartos como áreas peligrosas y permitir únicamente el acceso al personal autorizado. Los nombres y números telefónicos de los manejadores experimentados o de las personas responsables deben ser colocados en un lugar visible, en cada cuarto, en toda el área de alojamiento de los animales. Estas notas deben incluir instrucciones para el personal de servicio y seguridad en caso de que se presente alguna emergencia.

5.4.6.3. Jaulas individuales.

Deben ser amplias y altas de tal manera que proporcionen el espacio adecuado para la postura normal y los ajustes de su comportamiento. Deben ser sólidas, sus puertas deben estar provistas con broches o candados para cerrarlas, los cuales no puedan ser abiertos por los monos. Se debe evitar el contacto entre monos de diferentes jaulas.

Deben estar equipadas con pisos de malla de alambre y anaqueles de superficies lisas o perchas en las cuales los animales puedan descansar. El piso debe soportar el peso del animal, los orificios de la malla deben permitir el paso de las heces, asegurándose de que tanto brazos como piernas y cola no queden apresados en ellos. Los monos no deben alcanzar sus excretas.

Los depósitos de agua y alimento deben estar colocados de tal manera que no puedan ser contaminados con el excremento ni movidos por los animales. Son recomendables los sistemas de bebederos automáticos. Las jaulas deben estar equipadas con una puerta interior deslizable permitiendo la compresión de los animales al frente para la aplicación de inyecciones, medicamentos, tranquilizantes u otros. Es deseable que las cajas estén provistas con un entrepaño deslizable en la puerta para permitir la transferencia de los animales dentro de las jaulas de transporte.

5.4.6.4. Alojamiento en grupos.

Sólo los animales que sean compatibles deben ser alojados en grupo. Es más práctico y deseable tener a los animales alojados por grupos. Esta es una alternativa económica que puede ser explotada cuando sea apropiado una vez que los animales hayan completado su cuarentena. Se debe tener cuidado en algunas especies, especialmente en macacos adultos, ya que ocurren peleas frecuentes entre animales que no han sido alojados juntos desde la infancia. En este sistema son importantes factores como la dinámica poblacional, la proporción hembra-macho, la jerarquía y el comportamiento social, los cuales deben ser tomados en cuenta.

5.4.6.5. Condiciones ambientales.

Cuando se estén diseñando los alojamientos deben ser tomados en consideración el comportamiento, las necesidades sociales y emocionales. Cualquier primate alojado de manera individual sufrirá de privación social, lo cual le provocará estrés pudiendo causar alteraciones en los procesos psicológicos, fisiológicos y en su comportamiento.
La iluminación debe ser controlada de manera que se proporcionen tanto periodos de luz como de obscuridad con el fin de imitar el ambiente natural de los animales. En general, los ciclos 12:12 de luz/obscuridad son satisfactorios. Debe tomarse en cuenta si los animales son de actividad diurna o nocturna, si su reproducción es estacional, y si ésta depende de la duración del fotoperiodo.
La ventilación debe proveer de un mínimo de 12 a 15 cambios de aire por hora. Si la densidad de población es elevada, se debe incrementar a 18-20 cambios por hora.
La temperatura ideal depende de la especie. La mayoría de los primates del Viejo Mundo se pueden adaptar fácilmente a una temperatura de 19°C. En contraste, los monos del Nuevo Mundo y los prosimios deben ser mantenidos a temperaturas entre los 22 y 26°C. Los neonatos, por su poca habilidad para la termorregulación, deben ser mantenidos a temperaturas de 30-32°C.
La humedad relativa debe ser mantenida entre 45 y 60% para la mayoría de las especies y nunca se debe permitir su descenso más allá de 30%, ya que esto predispone a la presencia de enfermedades respiratorias. La humedad relativa debe ser mayor al 60% para aquellas especies tropicales del Nuevo Mundo.
5.4.6.6. Saneamiento.
Los vehículos y jaulas de transporte utilizados con estos animales deben ser lavados y desinfectados inmediatamente después de haber sido utilizados y previo a acomodar cualquier otra remesa de animales. Se deben tener precauciones especiales en la desinfección que se lleva a cabo en las jaulas de vivienda de las colonias de primates. Los guantes que se utilizan como protección deben ser desinfectados frecuentemente con óxido de etileno o gas de formaldehído. Toda la excreta debe ser manejada de tal manera que se asegure que no hay riesgos para la salud animal y humana, por ejemplo desinfección líquida o incineración. El instrumental, las jeringas, las agujas hipodérmicas y toda clase de equipo utilizado en los primates no humanos, así como la ropa de protección deben ser sometidos al autoclave antes de llevarse a la rutina de lavado y esterilización. Si se utiliza un desinfectante químico, éste debe tener propiedades viricidas, bactericidas y fungicidas. Cada cuarto debe tener su propia jaula de transferencia y material de limpieza.
5.4.7. Nutrición.
La mayoría de los primates utilizados en la investigación biomédica pueden ajustarse a una amplia variedad de dietas de laboratorio. Se debe tener en cuenta que, en vida libre los primates no humanos consumen una gran cantidad de hojas, frutos y, en ocasiones, insectos. En general, se debe procurar que la dieta basada en alimento comercial sea complementada con frutas y verduras frescas, para asegurar el aporte de fibra, proteína y vitaminas necesarios. Si esto no es posible, deben recibir suplementación con vitamina C (10 mg/kg/día) y los monos del Nuevo Mundo además se suplementen con vitamina D3 (1 U.I./g., de la dieta). También debe tomarse en cuenta que los monos del Nuevo Mundo requieren entre 5 y 10% más proteína cruda que los monos del Viejo Mundo y que las especies folívoras necesitan al menos un 10% de fibra cruda en su dieta. Los primates silvestres, recién importados deben ser sobrealimentados. La variedad es particularmente importante en los primates; sin embargo, deben evitarse los cambios bruscos en la dieta, ya que éstos a menudo provocan alteraciones entéricas. Una buena estrategia es alimentarlos con pequeñas porciones 2 o 3 veces al día para evitar problemas tales como la posibilidad de una distensión gástrica aguda. Se deben evitar los alimentos que contribuyan a incrementar las caries, pudiéndose incorporar dietas de fórmula constante conteniendo ingredientes activos para evitar la formación de sarro y sus secuelas. El consumo de agua para todos los monos debe ser de 80 ml/kg/día.

5.4.8. Reproducción.

Los programas de producción para primates no humanos deben ser llevados a cabo únicamente en aquellas instituciones con instalaciones específicamente diseñadas para propósitos reproductivos, con personal competente y experimentado.

5.4.9. Manejo.

5.4.9.1. Sujeción física.

En caso necesario, las jaulas para primates deben estar equipadas con un mecanismo para comprimir a los animales facilitando la inyección de agentes tranquilizantes. En muchos casos se utilizan jaulas de transferencia, en la cual el animal entra voluntariamente, ésta es mucho más pequeña y la contención manual se puede realizar de una manera más rápida. Los monos pequeños deben ser tomados o sujetados por arriba del codo con los brazos sostenidos atrás de su espalda y con sus piernas extendidas. Este procedimiento inmovilizará completamente al animal y lo pondrá en una posición en la cual le es imposible morder al manejador. Se debe tener cuidado en no fracturar los brazos y las piernas cuando esté siendo removido de su jaula. Los animales criados en cautiverio a menudo llegan a ser entrenados a la captura, contención y toma de muestras. Esto provoca que se ignoren las precauciones que se recomiendan, como es el uso de guantes especiales o un método apropiado de contención y propicia accidentes. Nunca se debe subestimar los peligros que provocan la fuerza, tenacidad e inteligencia de los primates no humanos.

5.4.9.2. Sujeción química.

Esta es aconsejable cuando los grandes monos van a ser removidos de sus jaulas. Se deben tener extremadas precauciones cuando existan manifestaciones de enfermedad o en animales débiles al utilizar los agentes tranquilizantes.

5.4.9.3. Uso de cerbatanas, pistolas o rifles narcotizantes.

El peligro asociado al manejo de primates no humanos puede ser reducido al mínimo, mediante el uso de equipos especialmente diseñados para la inmovilización química a distancia. Esto es realizado a través del empleo cuidadoso y bajo estricto criterio veterinario de cerbatanas, pistolas o rifles para la administración de drogas sedantes o anestésicas. En todo evento se debe procurar un uso en extremo cuidadoso de estos equipos para evitar infligir lesiones en los animales o personas.

5.4.9.4. Aspectos de seguridad.

a) Las jaulas deben cumplir con los requisitos de seguridad tanto para el personal como para los animales.

b) La sujeción física debe ser segura y considerada con el énfasis puesto en la protección animal y humana.

c) La sujeción química debe ser usada en grandes primates.

d) La extracción quirúrgica de dientes caninos puede ser desarrollada en primates que son frecuentemente manipulados. Alternativamente puede realizarse el recorte y la endodoncia de dientes caninos, al nivel del borde incisal de los dientes adyacentes. No es aceptable el recorte o la fractura de caninos sin el procedimiento endodóntico integral.

5.4.10. Toma de muestras y manipulación.

Para la toma de muestras sanguíneas se requiere tranquilizar al animal. Dependiendo de la especie y talla del animal, éstas pueden ser tomadas por la vena radial, la caudal, la femoral, la safena o bien por punción yugular (ver cuadro número 10).

Para la recolección de muestras de orina o heces se utilizan cajas metabólicas.

5.4.11. Anestesia.

Antes de cualquier intervención quirúrgica debe realizarse un perfil hematológico y bioquímico del animal.

5.4.11.1. Medicación preanestésica.

Dependiendo de la talla del animal se recomienda ayuno de sólidos de 12 a 24 horas y ayuno de líquidos de 12 horas antes de ser anestesiado. Se debe realizar premedicación con

tranquilizantes para calmar al animal y facilitar la inducción de la anestesia. Para esto se pueden utilizar los tranquilizantes y anestésicos en las dosis que indique el Médico Veterinario o el protocolo de investigación. Después de aplicar el inductor se deben reducir las secreciones del aparato respiratorio administrando sulfato de atropina.

5.4.11.2. Anestésicos inyectables o inhalados.

Cuando se requiera del uso de anestésicos, debe hacerse siempre bajo la supervisión del Médico Veterinario, previa aprobación del protocolo de experimentación por el Comité.

Siempre que se anestesia a un animal se deben monitorear los signos vitales.

Toda cirugía en primates no humanos debe realizarse de acuerdo con los principios de la misma.

5.4.12. Eutanasia.

La selección del método o forma de eutanasia y el agente que será utilizado necesita ser tomado en consideración de acuerdo con la naturaleza y requerimientos del estudio experimental, aprobado por el Comité. Es absolutamente necesario seleccionar un método que cause el menor sufrimiento a los animales. El método más apropiado para la eutanasia en primates no humanos consiste en administrar un agente inmovilizante o sedante de los señalados en esta Norma. Una vez que es lograda la inmovilización se administra por vía endovenosa una sobredosis de anestésicos.

5.4.13. Cuidados de la salud.

La introducción y el mantenimiento de un programa sanitario completo y un examen clínico regular de todos los primates no humanos, así como la compra de animales sanos y el traslado de animales enfermos a otra área, ayuda a disminuir la presencia de enfermedades en la colonia.

Las medidas preventivas incluyen:

a) La frecuente inspección veterinaria de los animales para detectar signos de enfermedad.

b) Separación y aislamiento de animales enfermos e implantación de diagnóstico y terapia inmediata.

c) Tratamiento rutinario contra endo y ectoparásitos frecuentemente transmitidos por vectores como aves, roedores silvestres u otros.

d) Empleo de vacunas para inmunización contra poliomielitis, parotiditis y sarampión (chimpancés, gorilas y orangutanes). La vacunación contra tétanos o rabia quedará a criterio veterinario, de acuerdo con las condiciones prevalentes.

e) Deberán existir registros detallados de tipo clínico de todos los primates de las colonias, así como de los eventos previos a la muerte de un animal.

f) Se debe congelar suero de los primates no humanos a -70°C para su referencia en caso de presentación de alguna enfermedad de etiología confusa.

5.4.13.1. Cuidados generales.

Los animales nunca deben estar mezclados en una misma área con los de otra especie animal.

Se debe tener control periódico de la potabilidad del agua y el alimento de los animales para evitar problemas de infecciones o parasitosis.

Todo el personal debe bañarse después de manejar a los animales, así como usar ropa específica por área de trabajo; usar gorras y cubrebocas desechables.

El personal que trabaje con primates no humanos debe realizarse, al menos cada seis meses, un estudio coproparasitoscópico, un examen sanguíneo, un examen serológico (hepatitis, VIH, etc.), un exudado faríngeo. Además deben realizarse placas de tórax, así como una inmunización contra tétanos cada 3 o 4 años, o a juicio del médico.

También hay que impedir la entrada de insectos, roedores, así como otros mamíferos, que puedan transportar agentes patógenos para el hombre o los animales, aun cuando no presenten signo alguno.

Es deseable conservar suero a -70°C del personal que labora con primates no humanos desde el primer día que entren en contacto con ellos.

5.4.13.2. Cuidados particulares.
Se debe monitorear cotidianamente el comportamiento de los animales, tanto de los alojados en jaulas individuales, como de los que viven en grupos sociales, con especial atención a conductas estereotipadas o repetitivas, autoagresiones y la manipulación o ingesta de heces; estas conductas son un claro índice de que los animales se encuentran estresados.
La evaluación periódica de salud debe prestar especial atención a la higiene bucal de los primates. La formación de sarro debe ser tratado periódicamente de acuerdo con el criterio veterinario. Asimismo, se puede prevenir esta condición, mediante el uso de dietas comerciales especialmente formuladas para evitar la formación de sarro y con ello sus secuelas tardías como la gingivitis o la enfermedad periodontal.
5.5. Porcinos.
5.5.1. Confinamiento o encierro primario.
Cada corral de las diferentes áreas debe estar equipado con comedero y bebedero o tazón de material no corrosivo o de alimentación automática. Dependiendo de la talla corporal, el espacio disponible del comedero debe tener entre 20 y 40 cm por animal.
Para propósitos experimentales, cuando los porcinos se alojen en lugares cerrados y jaulas especiales, éstas deben ser de material resistente y fácil de limpiar. Los espacios requeridos para porcinos de diferente peso corporal se indican en el cuadro número 6.
Los porcinos que se utilicen para experimentación, deben estar clínicamente sanos y ser tratados de acuerdo al protocolo de experimentación aprobado por el Comité.

CUADRO No. 6
ESPACIO RECOMENDADO PARA PORCINOS DE DIFERENTE PESO CORPORAL

Cantidad de animales	Peso Corporal Kg.	Area de Piso/ Animal m^2
1	<15	0.75
	Hasta 25	1.12
	Hasta 50	1.40
	Hasta 100	2.23
	Hasta 200	4.46
	>200	5.58
2-5	<25	0.56
	Hasta 50	0.93
	Hasta 100	1.86
	Hasta 200	3.72
	>200	4.84
>5	<25	0.56
	Hasta 50	0.84
	Hasta 100	1.68
	Hasta 200	3.35
	>200	4.46

5.5.2. Salud animal.
Los porcinos son particularmente sensibles a temperaturas extremosas. Para proteger a los animales del frío, los pisos y paredes de la construcción deben estar bien aislados y con circulación de aire controlado. Los animales mantenidos en pisos de rejilla o malla de alambre deben tener un sistema de calefacción y ventilación. Los corrales deben lavarse y reemplazarse las camas mojadas diariamente. Por lo menos una vez al mes deben evacuarse los corrales. Los pisos, paredes, jaulas y utensilios deben remojarse con solución desinfectante y lavarlos con agua a presión.
5.5.3. Alimento y agua.

Los requerimientos nutricionales para animales de diferentes edades y estado reproductivo con especial atención en proteína y máximo de fibra cruda se indican en el cuadro número 7. El contenido de lípidos en la mayoría de las raciones es de 3-3.5%.
El agua debe ser potable y estar a libre acceso, se recomienda usar bebederos automáticos.
5.5.4. Cama y nido.
Por razones higiénicas (parásitos internos y externos) los porcinos son mantenidos en pisos sólidos, rejilla o concreto. Dependiendo de la edad de los porcinos, las rejillas varían en anchura de 5-12 cm, con aberturas de 1.5-3 cm. Los pisos sólidos usualmente están provistos de camas de paja, aserrín o viruta de madera.
5.5.5. Agrupamiento.
Los porcinos deben agruparse por edad, peso y talla preferentemente.
5.5.6. Manipulación e inmovilización.
Los porcinos al manejarlos son generalmente temerosos y excitables, tercos e individualistas, pueden mostrar histeria y ser extremadamente ruidosos cuando se les confina. Tanto al verraco adulto como a la cerda en el nido se les debe acercar con precaución.
Para manejar grupos de porcinos, usualmente se hace caminando detrás de éstos con la ayuda de tablas para orientar a los animales. Los lechones de hasta 5 kg deben sujetarse con ambos brazos. Animales demasiado pesados pueden sujetarse con lanza trompas colocados en la región caudal de los dientes caninos. Es recomendable el uso de tranquilizantes en diversos casos, a consideración del Médico Veterinario.

CUADRO No. 7
REQUERIMIENTO DE PROTEINA Y FIBRA EN UNA RACION PARA PORCINO

**Categorías Del porcino**	**Proteína Cruda %**	**Máximo Fibra Cruda %**
1.5-5 kg	23	2
6.0-20 kg	20	6
21.0-40 kg	16	6
41.0-130 kg	14	7
Cerdas <12 Sem. de gestación	12	14
Cerdas >12 Sem. de gestación	16	7
Cerdas amamantando 10 lechones	16	7
Cerdas post destete.	16	7
Sementales	16	7
Grupo en experimentación	12	14

6. Instalaciones.

Las instalaciones destinadas para el alojamiento de animales de laboratorio, serán diseñadas de acuerdo con las necesidades de los sujetos experimentales, de los usuarios y del personal que interviene en su cuidado diario. Un diseño satisfactorio debe permitir la existencia de sectores definidos de alojamiento animal, de experimentación y apoyo a la operación, así como áreas especializadas de atención o cuidado animal en donde así se justifique. Los criterios de diseño de las instalaciones para animales de laboratorio deben ser sancionados mediante la opinión calificada de un Médico Veterinario, a fin de asegurar y favorecer niveles satisfactorios de cuidado animal.
6.1. Instalaciones interiores bajo techo.
Los bioterios que se encuentran en recintos interiores, independientemente de su uso, función y especies animales que alojen, podrán ser construidos en un segmento, ala, nivel o espacio físico del edificio o terreno de la institución responsable de su funcionamiento, con la previsión

de que éstos mantengan una independencia física efectiva que asegure tanto una operación eficiente como libre de variaciones indeseables que afecten la respuesta experimental del sujeto.

6.1.1. Localización y ubicación.

Las áreas destinadas al alojamiento animal deben ubicarse en forma independiente a las de ocupación humana, con el fin de asegurar una operación higiénica y favorecer la salud y el confort de ambos.

6.1.2. Areas para animales.

La naturaleza de la actividad y las necesidades de la institución, definirán las áreas específicas de apoyo a la operación que éstos deben poseer. En general todo bioterio debe contar con sectores básicos, que aseguren el alojamiento animal, la separación física de especies o su aislamiento, la experimentación, así como un sector específico de apoyo a la operación.

6.1.2.1. Recepción de animales.

Este sector debe constituir un espacio independiente del resto de las áreas de animales y será destinado a dar alojamiento a aquellos sujetos de nueva adquisición, evitando así el contacto de éstos con los de colonias ya establecidas y la posible transmisión de enfermedades. Sus dimensiones deben contemplar las acciones inherentes al manejo, acomodo y control de los sujetos involucrados, así como permitir una operación higiénica e independiente para cada especie animal empleada.

6.1.2.1.1. Cuarentena y acondicionamiento.

El bioterio debe contar con un sector específico e independiente para este propósito, el cual debe evitar el contacto con colonias ya establecidas y la posible transmisión de enfermedades. Sus dimensiones dependerán de la cantidad y variedad de animales adquiridos de fuentes externas. Este cuarto debe permitir la separación física de especies y la realización de evaluaciones y tratamientos cuarentenarios, indispensables para favorecer un periodo de acondicionamiento óptimo en los animales, previo a su uso experimental, durante el cual se logra su estabilización y se familiarizan con sus nuevas instalaciones.

6.1.2.2. Cuartos para producción y mantenimiento de los animales.

Cualquier local destinado para mantener animales de laboratorio por 24 horas o más, es considerado un cuarto de alojamiento. Las diferentes especies de animales de laboratorio deben ser alojadas en cuartos independientes, a menos que el Comité autorice lo contrario, de acuerdo a las características del protocolo de investigación o el propósito experimental o reproductivo al cual estén sujetos. Los bioterios deben contar con cuartos especiales para el alojamiento y/o aislamiento de sujetos inoculados con radioisótopos, agentes infecciosos o bien sustancias tóxicas.

6.1.2.3. Cuarto para procedimientos diversos o de uso múltiple.

Dependiendo del programa de trabajo del bioterio, éste debe contar con un cuarto de procedimientos diversos, para la manipulación experimental de animales, sus tratamientos, recolección de fluidos corporales o necropsias, identificación, preparación quirúrgica u otros.

6.1.2.3.1. Area de cirugía.

Sector del bioterio destinado para y equipado de acuerdo con el desarrollo de procedimientos experimentales, analíticos o de enseñanza que requieran el empleo de técnicas quirúrgicas con supervivencia del animal en condiciones asépticas. Sus dimensiones y equipamiento estarán en función de las necesidades de la institución. Si se realiza cirugía mayor con supervivencia del animal, se recomienda la existencia de un quirófano. Otras cirugías sólo requieren técnicas asépticas. Si no hay supervivencia sólo se sugieren técnicas “limpias”.

6.1.2.3.2. Area de recuperación.

Destinada y equipada de acuerdo con las necesidades de atención de sujetos sometidos a procedimientos invasivos, de conformidad con el criterio veterinario. Sus dimensiones y equipamiento estarán en función de las necesidades de la institución.

6.1.2.4. Areas de apoyo.

Dependiendo del programa de trabajo, los servicios de los bioterios deben integrarse a través de diferentes sectores de apoyo a la operación, mismos que permiten un funcionamiento armónico.

6.1.2.4.1. Almacén de consumibles.

Debe existir un almacén para guardar el material de cama limpia, como viruta de madera u otros, de los animales de bioterio. Este depósito conjuntamente con el del alimento debe mantener una independencia efectiva (a prueba de fauna nociva y contaminación) de los insumos destinados para animales de laboratorio, a fin de evitar la infestación con roedores silvestres.

6.1.2.4.2. Almacén de equipo.

Los bioterios deben contar con un almacén de equipo que permita guardar jaulas y accesorios de uso rutinario.

6.1.2.4.3. Area de preparación de dietas.

En bioterios donde el programa de trabajo así lo justifique y a juicio del Médico Veterinario, debe existir una zona destinada y equipada de acuerdo con esta actividad. Su diseño debe permitir el mantener los insumos en un lugar independiente, fresco y libre de contaminación o fauna nociva indeseable.

6.1.2.4.4. Area de lavado.

Debe ser localizada en forma independiente a las áreas de alojamiento animal, evitando que el ruido generado en ésta disturbe secciones de ocupación animal o humana. Contará con el equipo necesario para satisfacer los requerimientos de limpieza y desinfección del bioterio.

6.1.2.4.5. Depósito y eliminación de desechos del bioterio.

Debe ser localizada de manera independiente a las áreas de alojamiento animal y equipada con refrigeradores y otros equipos, de acuerdo con la necesidad de mantener un manejo higiénico de la misma. Los desechos tales como cadáveres, órganos o sus partes, tejidos, líquidos corporales, materiales punzocortantes, jeringas y agujas hipodérmicas, entre otros, considerados residuos peligrosos biológico-infecciosos, serán manejados y eliminados de acuerdo con la Norma NOM-087-ECOL-94, así como a la Ley General del Equilibrio Ecológico y la Protección al Ambiente. Los desechos de los bioterios que no son contaminantes o biológicos infecciosos, deben ser destinados al depósito general de basura.

6.1.2.4.6. Areas de descanso y vestidores para el personal.

El bioterio debe contar con un sector independiente que permita al personal involucrado en su operación, el disponer de un sector para descansar y tomar sus alimentos, de manera independiente a los cuartos de animales. Asimismo, deben existir regaderas provistas de agua corriente fría y caliente y sanitarios que permitan una operación de máxima higiene y seguridad en el trabajo. En lo relativo a esto último se debe seguir el criterio de lo dispuesto en la NOM-018-STPS-1993. Las instalaciones contarán con lavabos provistos de agua corriente, jabón, toallas desechables o secadores de aire y un bote de basura. De existir más de siete trabajadores en el establecimiento, se debe contar con un mínimo de dos excusados. En el caso de servicios sanitarios que carezcan de ventilación natural, se proveerán de extractores de aire.

6.1.2.4.7. Area de administración y control del bioterio.

Los bioterios como unidades prestadoras de servicios de apoyo, deben contar con un sector de control de la operación, en donde se procesen todos los aspectos relacionados con su trabajo diario. Sus dimensiones deben ser conmensurables con el volumen de animales y diferentes especies mantenidas.

6.1.3. Especificaciones de acabados sanitarios.

Los bioterios deben ser provistos con acabados sanitarios que primariamente favorezcan la higiene como medio de prevención o diseminación de enfermedad, de acuerdo con el criterio veterinario.

6.1.3.1. Pisos.

Los pisos deben ser de superficie lisa, impermeable y de resistencia satisfactoria, como para permitir durabilidad, facilidad de limpieza y desinfección con sustancias químicas u otro método. Los encuentros de pared-piso deben contar con un zoclo sanitario que evite la anidación de insectos y basura.

6.1.3.2. Paredes y techos.

Las paredes de los cuartos de animales deben poseer resistencia e impermeabilidad, sus acabados deben estar libres de juntas imperfectas y oquedades. Los techos deben ser de superficie lisa y carentes de grietas.

6.1.3.3. Puertas y ventanas.

Los cuartos de animales contarán con puertas resistentes y durables y sus características de construcción deben impedir la entrada de fauna nociva. Los recintos interiores para el alojamiento de animales de laboratorio no poseerán ventanas colindando con el exterior, dado que ello impacta desfavorablemente en la definición ambiental o en las condiciones medio ambientales que pueden afectar los experimentos. Se podrán tener ventanas para aquellas especies donde este elemento se considere como parte del enriquecimiento ambiental.

6.1.3.4. Pasillos.

Los pasillos deben comunicar de manera eficiente las diferentes secciones del bioterio. La altura y el ancho deben permitir el paso holgado del equipo. Preferentemente los remates de las esquinas deben poseer protección adecuada y los corredores que conduzcan a zonas de gran ruido (secciones de lavado, alojamiento de perros o primates u otros) deben contar con trampas de ruido que procuren la estabilidad ambiental.

6.1.3.5. Tubería y maquinaria.

Las tuberías de las instalaciones deben identificarse de acuerdo con el material que transporten, de conformidad con los códigos de colores estipulados en la NOM-028-STPS-1994. Las líneas correspondientes a servicios diversos, eléctricos o hidráulicos deben colocarse en los corredores de circulación, para evitar sean atendidos en el interior de los locales.

Cada cuarto de animales debe contar con instalaciones de luz eléctrica y contactos que cumplan con lo dispuesto en la Ley de Servicio Público de Energía Eléctrica y su Reglamento.

En caso de que las instalaciones cuenten con una sala de máquinas, ésta debe estar independiente de las áreas de alojamiento animal, evitando con ello que las vibraciones o ruidos indeseables pudiesen afectar la definición ambiental del bioterio.

6.1.4. Control del medio ambiente.

El medio ambiente debe ser objeto de máxima atención para el animal de laboratorio, a fin de evitar que aun pequeñas variaciones de éste afecten negativamente la respuesta experimental.

6.1.4.1. Temperatura.

Las instalaciones de animales de laboratorio ya sean para reproducción o experimentación, deben mantener una temperatura estable dentro de los cuartos, misma que oscilará de acuerdo a lo establecido en el cuadro número 8.

CUADRO No.8
RANGOS DE TEMPERATURA (°C) Y HUMEDAD RELATIVA (%)

Animal	°C	%
Ratón, rata, hámster, jerbo, cobayo	18-26	40-70
Conejo	16-26	40-70
Perro, gato, primates no humanos	18-29	40-70
Porcinos	16-27	40-70

Nota:

Temperatura tomada con termómetro de bulbo seco.

6.1.4.2. Humedad relativa.
Las instalaciones que alojan animales de laboratorio, deben proveer una humedad relativa entre el 40 y 70%.
6.1.4.3. Ventilación.
Las instalaciones para animales de laboratorio deben poseer un sistema de ventilación eficaz, que permita un recambio de aire ambiental que cubra un rango mínimo de 15 a 18 recambios de aire por hora. El sistema debe funcionar ininterrumpidamente las 24 horas del día, a fin de favorecer una definición ambiental aceptable que no afecte negativamente la salud animal y la respuesta experimental.
6.1.4.4. Iluminación.
Las instalaciones del bioterio estarán iluminadas mediante luz artificial tipo luz de día usando lámparas fluorescentes. El control de los ciclos de luz se efectuará por medio del uso de relojes interruptores automáticos, ajustados de acuerdo con las necesidades de los animales en cuestión. La intensidad lumínica no debe exceder de 1,345 lúmenes para el desarrollo de tareas generales de limpieza, observación y registro dentro de los locales. Sin embargo, debe considerarse la recomendación de mantener 300 lúmenes de intensidad lumínica, para áreas de alojamiento de roedores.
6.1.4.5. Ruido.
Las instalaciones dedicadas al alojamiento de animales de laboratorio deben contar con dispositivos de contención y control de ruido en equipos rodables, carros de servicio y en áreas que generan ruidos excesivos, ya sean de lavado o bien de especies como perros y primates no humanos. La intensidad de ruido no debe ser mayor a 85 dB. El control anterior debe ser alcanzado mediante buenas prácticas de cuidado animal y la orientación del personal de apoyo.
6.2. Instalaciones en exteriores.
Los refugios o alojamientos al aire libre tales como corrales, pastizales, etcétera, son el método común de alojamiento para algunos animales y es aceptable. Se debe brindar protección contra temperaturas extremas y otras condiciones climáticas adversas, así como mecanismos protectores y de evasión para los animales subordinados. Los refugios deben estar accesibles a todos los animales, tener ventilación suficiente y evitar la acumulación de desperdicio y humedad excesiva. Los pisos pueden ser de tierra, material de lecho, arena, grava, paja o materiales similares que puedan ser reemplazados. Los materiales de construcción deben ser resistentes a la intemperie y fáciles de limpiar, y el diseño debe evitar en lo posible las lesiones en los animales.
Los ambientes naturales pueden ser los idóneos para el mantenimiento o reproducción de algunas especies animales y de algunos tipos de investigación, por lo tanto son aceptables.
6.2.1. Localización.
Los bioterios que se encuentran en recintos exteriores, independientemente de su uso, función y tipo de animales que alojen, deben ser ubicados en un segmento independiente de edificios no relacionados con su actividad, a fin de minimizar el acceso o contacto de personal no autorizado con los animales ahí alojados.
6.2.1.1. Relación física entre instalaciones y oficinas.
Las áreas destinadas al alojamiento animal deben ubicarse en forma independiente a las de ocupación humana, con el fin de asegurar una operación higiénica y favorecer la salud y el confort de ambos. El sitio elegido debe estar libre de contaminación de cualquier tipo, ruidos y vibraciones causados por maquinarias cercanas y debe estar debidamente resguardado de inundaciones y fenómenos meteorológicos.
Deben considerarse los factores ambientales del área seleccionada en cuanto a vientos dominantes, precipitación pluvial, sombra, así como la posición del edificio(s) con relación a la luz del sol.

6.2.2. Areas separadas.
Los bioterios exteriores deben contar con la capacidad de aislamiento para animales que resultaren enfermos, agredidos por sus congéneres, o bien que requieran a juicio veterinario o del proyecto de investigación de dicha independencia temporal o permanente. Estas deben estar debidamente acondicionadas para favorecer prácticas idóneas de alojamiento y cuidado diario de los sujetos experimentales.
6.2.3. Areas del bioterio.
La naturaleza de la actividad, así como las especies animales involucradas, definirán las áreas específicas de apoyo a la operación que el bioterio debe poseer. En general se debe contar con sectores básicos que aseguren el alojamiento animal, la separación física de especies o su aislamiento, la experimentación, así como un lugar específico de apoyo a la operación.
6.2.3.1. Recepción.
Este sector debe constituir un espacio independiente del resto de las áreas de animales y será destinado a dar alojamiento a aquellos sujetos de nueva adquisición, evitando así el contacto de éstos con los de colonias ya establecidas y la posible transmisión de enfermedad. Sus dimensiones deben contemplar las acciones inherentes al manejo, acomodo y control de los sujetos involucrados, así como permitir una operación higiénica e independiente para cada especie animal empleada, de acuerdo con el criterio veterinario.
6.2.3.1.1. Cuarentena y acondicionamiento.
El bioterio debe contar con un sector específico e independiente para este propósito. Sus dimensiones dependerán de la cantidad y variedad de animales adquiridos de fuentes externas. Este cuarto debe permitir la separación física de especies y la realización de evaluaciones y tratamientos cuarentenarios, indispensables para favorecer un periodo de acondicionamiento óptimo en los animales, durante el cual se logra su estabilización y se familiarizan con sus nuevas instalaciones, previo a su uso experimental.
Las instalaciones podrán contemplar el alojamiento en grupo o en forma individual, con la previsión de que todo sector de alojamiento posea dimensiones conmensurables con el número de animales involucrados, así como un albergue apropiado, de acuerdo con el criterio veterinario.
6.2.3.2. Locales para producción y mantenimiento de los animales.
Cualquier local destinado a la manutención de animales de laboratorio por 24 horas o más, debe considerarse un cuarto de alojamiento. Las diferentes especies deben ser alojadas en cuartos independientes, de acuerdo con los proyectos de investigación o el propósito experimental o reproductivo al cual estén sujetos. Estos deben contar con sectores específicos para el alojamiento y/o aislamiento de sujetos inoculados con radioisótopos, agentes infecciosos o bien sustancias tóxicas.
El alojamiento en jaulas tipo corral de perros, gatos, primates no humanos y otras especies en grupos puede resultar en gestaciones indeseables, lesiones por agresiones, así como en la transmisión de enfermedades, por lo que no serán aceptables estas condiciones para animales sometidos a experimentos crónicos.
Debe proveerse espacio suficiente durante etapas críticas como la gestación y la lactancia, para que los animales se reproduzcan y se mantengan en condiciones apropiadas, considerando invariablemente la necesidad de un albergue en contra de condiciones climáticas adversas.
6.2.3.3. Cuarto para procedimientos diversos de uso múltiple.
Dependiendo de la orientación del programa de trabajo del bioterio, éste debe contar con un cuarto de procedimientos diversos para la manipulación experimental de animales, sus tratamientos, recolección de fluidos corporales, identificación, preparación quirúrgica u otros.
6.2.3.3.1. Area de cirugía.
Sector del bioterio destinado y equipado de acuerdo con el desarrollo de procedimientos experimentales, analíticos o de enseñanza e investigación, que sugieran el empleo de técnicas

quirúrgicas en condiciones asépticas. Sus dimensiones y equipamiento estarán en función de las necesidades de las especies animales mantenidas por la institución, de conformidad con el criterio veterinario.

6.2.3.3.2. Area de recuperación.

Destinada y equipada de acuerdo con las necesidades de atención de sujetos sometidos a procedimientos invasivos. Su diseño, dimensiones y equipamiento estarán en función de las necesidades de las especies animales de la institución, de conformidad con el criterio veterinario.

6.2.3.3.3. Areas de radiología y diagnóstico clínico.

Destinadas y equipadas de acuerdo con las necesidades de atención de esta actividad, de conformidad con el criterio veterinario. Su existencia puede omitirse en lugares o instituciones en donde exista apoyo externo comprobable.

6.2.3.4. Areas de apoyo.

Los servicios de los bioterios deben integrarse a través de diferentes sectores de apoyo a la operación, mismos que permiten un funcionamiento armónico. Las dimensiones de éstos dependerán del programa de trabajo de la institución y de su magnitud.

6.2.3.4.1. Almacén de consumibles.

Debe existir un almacén para la guarda de material de cama limpia (viruta de madera, paja, u otros) de los animales. Este depósito conjuntamente con el del alimento debe mantener una independencia efectiva (a prueba de fauna nociva y contaminación), a fin de evitar la infestación con animales silvestres. En lugares de clima extremoso debe existir un cuarto frío y seco que mantenga en condiciones óptimas los alimentos. Su localización debe estar cercana a los sitios de suministro y manejo de los insumos, y poseer corredores de enlace que favorezcan una distribución eficiente mediante carros de servicio, carretillas u otros dispositivos apropiados para este fin.

6.2.3.4.2. Almacén de equipo.

Debe existir un almacén de equipo limpio independiente, que permita guardar jaulas y accesorios de uso rutinario. El espacio proporcionado para esta actividad debe cubrir un mínimo de 11% de la superficie total de las instalaciones, con la excepción de bioterios cuya orientación de trabajo y número de especies requiera de un mayor porcentaje de espacio.

6.2.3.4.3. Area de preparación de dietas.

En bioterios en donde el programa de trabajo así lo justifique a juicio del médico veterinario, debe existir una zona destinada y equipada de acuerdo con esta actividad. Su diseño debe permitir el mantener los insumos en un lugar independiente, fresco y libre de contaminación o fauna nociva; su equipamiento debe permitir la preparación de dietas o raciones.

6.2.3.4.4. Area de lavado.

Los bioterios exteriores podrán contar con un área de lavado central para el procesamiento de jaulas y estantes rodables, misma que debe ser localizada en forma independiente a las áreas de alojamiento animal.

Su equipamiento puede contar con máquinas de lavado y sanitizado mecánico, fosas de inmersión y desinfección de jaulas o un dispositivo equivalente. Sus servicios incluirán agua fría, caliente, aire comprimido, vacío y vapor para uso de autoclave o equipos mecánicos de lavado, así como una separación física de áreas limpia y sucia. Los accesos a esta área deben contemplar corredores de tránsito, hechos de piso de concreto, asfalto o bien otro material equivalente.

Los recintos acondicionados para el alojamiento colectivo y permanente de diversas especies, deben contar con dispositivos y equipos de lavado consistentes en mangueras de rocío de alta presión o equipo portátil equivalente, para la limpieza y desinfección de los locales.

6.2.3.4.5. Depósito y eliminación de desechos del bioterio.

Debe ser localizado de manera independiente a las áreas de alojamiento animal y equipada con refrigeradores y otros equipos, de acuerdo con la necesidad de mantener un manejo

higiénico del mismo. Los desechos tales como cadáveres, órganos o sus partes, tejidos, líquidos corporales, materiales punzocortantes, jeringas y agujas hipodérmicas, entre otros, considerados residuos peligrosos biológico-infecciosos, serán tratados y eliminados de acuerdo con la Norma NOM-087-ECOL-94, así como a la Ley General del Equilibrio Ecológico y la Protección al Ambiente.

6.2.3.4.6. Areas de descanso y vestidores para el personal.

Debe existir un sector independiente que permita al personal involucrado en su operación, el disponer de un lugar para descanso y toma de alimentos de manera independiente a los cuartos de animales.

Asimismo, deben existir regaderas con agua fría y caliente y sanitarios que permitan una máxima higiene y seguridad en el trabajo. En lo relativo a esto último se debe seguir el criterio de lo dispuesto en la Norma Oficial Mexicana NOM-018-STPS-1993. Las instalaciones contarán con lavabos provistos de agua corriente, jabón, toallas desechables o secadores de aire y un bote de basura. De existir más de siete trabajadores en el establecimiento, se debe contar con un mínimo de dos excusados. En el caso de servicios sanitarios que carezcan de ventilación natural, se proveerán de extractores de aire.

6.2.3.4.7. Area de administración y control del bioterio.

Los bioterios como unidades prestadoras de servicios de apoyo, deben contar con un sector de control de la operación, en donde se procesen todos los aspectos relacionados con su trabajo diario. Sus dimensiones deben ser conmensurables con el volumen de animales y las diferentes especies mantenidas.

6.2.4. Especificaciones de acabados sanitarios.

Los acabados sanitarios primariamente deben favorecer la higiene como medio de prevención o diseminación de enfermedad, de acuerdo con el criterio veterinario.

6.2.4.1. Pisos y drenajes.

Los animales susceptibles de mantenerse en lugares exteriores, deben mantenerse en instalaciones cuyos pisos en general sean monolíticos y antiderrapantes. Su acabado podrá ser a base de concreto o un equivalente que permita su fácil limpieza y resista la frecuente desinfección y el ataque de sustancias químicas o el uso de rocío de alta presión.

Los encuentros de pared-piso deben contar con un zoclo sanitario que evite la anidación de insectos y la acumulación de basura. Los pisos deben poseer una pendiente apropiada cercana al 2% y una superficie lisa, que evite la formación de charcos de agua u orina. De existir coladeras, éstas deben estar apropiadamente rematadas y aseguradas al piso, para impedir el retorno de líquidos residuales, olores, así como lesiones a los animales. Las líneas de desagüe deben contar con hasta 18 cm de diámetro.

6.2.4.2. Paredes y techos.

Las paredes de los cuartos de animales podrán ser mixtas constando de una parte de mampostería de 0.90 cm de altura o completas, a la vez que poseer una separación de tipo malla ciclónica en el resto de la pared, apropiadamente anclada en sus extremos con tubos galvanizados libres de aristas o salientes agudas. Las paredes de las instalaciones deben poseer resistencia e impermeabilidad, sus acabados deben estar libres de juntas imperfectas y oquedales y podrán ser pintadas con agregados de tipo epóxico de colores claros. Los techos pueden estar formados por láminas de asbesto o losa de concreto, cubriendo éstos una superficie considerable de la jaula para proveer a los animales de abrigo en contra del sol o la lluvia.

Las jaulas en su conjunto, podrán poseer de acuerdo con el criterio veterinario, un sistema de doble pared exterior, hecho de tela de alambre o nylon tipo mosquitero, que impida en ciertas áreas la contaminación del alimento por moscas, pájaros silvestres, roedores, o bien el ingreso de mosquitos.

6.2.4.3. Puertas.

Los recintos para animales contarán con puertas resistentes y durables. Debe existir invariablemente una doble puerta o corredor de ingreso que evite el escape de los animales.
6.2.4.4. Ventanas.
Los recintos exteriores sin ventilación mecánica podrán tener ventanas que deben colocarse en uno de los lados contrarios a los vientos dominantes, a fin de evitar corrientes de aire; éstas podrán ser provistas de cortinas de plástico o lona para ser usadas en las noches. Las salidas de aire en estas instalaciones deben colocarse en los puntos más altos del edificio para remover en forma efectiva el aire caliente y la humedad del recinto. En instalaciones mecánicamente ventiladas, las salidas de aire deben ser colocadas de manera que el aire no tenga que recorrer más de 12 m para llegar al ventilador y su colocación debe ser hacia el lado de los vientos dominantes. En instalaciones para primates no es recomendable la existencia de ventanas.
6.2.4.5. Pasillos.
Los pasillos deben comunicar de manera eficiente las diferentes secciones de las instalaciones. La altura y el ancho deben permitir el paso holgado del equipo. Preferentemente los remates de las esquinas deben poseer protección adecuada y los corredores que conduzcan a zonas de gran ruido (secciones de lavado, alojamiento de perros o primates u otros) deben contar con trampas de ruido que procuren la estabilidad ambiental.
6.2.4.6. Tubería y maquinaria.
Las tuberías de las instalaciones deben identificarse de acuerdo con el material que transporten, de conformidad con los códigos de colores estipulados en la NOM-28-STPS-1994. Las líneas correspondientes a servicios diversos, eléctricos o hidráulicos deben colocarse en los corredores de circulación, o bien en el exterior de los cuartos para evitar sean atendidos en el interior de los locales. Esta recomendación es especialmente necesaria en instalaciones alojando primates.
Cada cuarto de animales debe contar con instalaciones de luz eléctrica y contactos que cumplan con lo dispuesto en la Ley de Servicio Público de Energía Eléctrica y su Reglamento. Los contactos eléctricos no deben ser colocados en sitios donde puedan ser salpicados por líquidos de cualquier origen, debiendo colocarse a 1.20 m de altura del nivel del piso, o estar suspendidos del techo. En el caso de primates, estas instalaciones deben estar apropiadamente resguardadas y no ser accesibles a ellos. En caso de que las instalaciones cuenten con una casa de máquinas, ésta debe estar independiente de las áreas de alojamiento animal, evitando con ello que las vibraciones o ruidos indeseables afecten la salud animal.
6.2.5. Control del medio ambiente.
En recintos exteriores situados en climas extremosos debe existir un sistema de ventilación o calefacción que asegure el confort de los animales. Las jaulas-corral que combinan la posibilidad de un refugio efectivo para los animales, deben asimismo contar con un sistema de control de la temperatura en donde a juicio del médico veterinario ello fuese indispensable.
6.2.5.1. Temperatura.
En instalaciones exteriores en donde no sea posible lograr un punto de equilibrio de temperatura compatible con el confort animal, debe proveerse con albergues apropiados que promuevan una condición aceptable. En lugares con climas extremos debe implementarse el uso de calentadores o ventiladores capaces de mantener dicha condición.
6.2.5.2. Iluminación.
Las instalaciones que combinan alojamientos tipo corral interior-exterior, deben poseer luz artificial tipo luz de día usando lámparas fluorescentes. El control de los ciclos de luz se efectuará por medio del uso de relojes interruptores automáticos, ajustados de acuerdo con las necesidades de los animales en cuestión.
6.2.5.3. Ruido.

Las instalaciones exteriores dedicadas al alojamiento de animales de laboratorio no deben construirse en forma aledaña a casas de máquinas o lugares capaces de emitir ruidos indeseables que alteren la conducta y hábitos de los animales.

7. Movilización

7.1. Consignación de los animales.

7.1.1. Aceptación del transportista o intermediario.

7.1.1.1. Lapso previo a la partida.

Ningún transportista o intermediario aceptará del consignador, privado o gobierno, animales con un máximo de seis horas antes de la hora de partida programada. Se recomienda que los animales permanezcan menos de cuatro horas en las estaciones de embarque.

7.1.1.2. Condiciones del confinamiento o encierro primario.

Ningún transportista o intermediario aceptará para transportación animales recluidos en confinamientos o encierros primarios que no cumplan con la presente Norma.

7.1.2. Condiciones de la terminal de embarque.

7.1.2.1 Los transportistas o intermediarios responsables del transporte de animales de laboratorio, deberán asegurar y, en su caso, tomar las precauciones necesarias, para evitar que los animales objeto de embarque, en cualquiera de sus modalidades, permanezcan a la intemperie o bien se expongan a temperaturas extremas que afecten su salud y bienestar.

7.1.3. Notificación al consignatario.

7.1.3.1. El transportista o intermediario debe notificar al consignatario la llegada de los animales inmediatamente después del arribo. En la documentación correspondiente se anotará la hora y método de cada uno de los intentos de notificación, así como la notificación exitosa al consignador y nombre de la persona que hizo la notificación.

7.2. Confinamiento o encierro primario para transportación.

7.2.1. Características del confinamiento o encierro primario.

Ningún transportista o intermediario debe trasladar animales recluidos en confinamientos o encierros primarios que no ofrezcan las siguientes características.

7.2.1.1. Solidez e inocuidad.

a) Los confinamientos o encierros primarios tales como compartimentos, jaulas para transporte, cajas de cartón o embalajes deben estar construidos de tal forma que ofrezcan la fuerza estructural suficiente para contener animales vivos y para soportar los rigores normales del transporte.

b) El interior del confinamiento o encierro primario debe estar libre de protuberancias que puedan causar lesiones a los animales ahí contenidos.

c) Para el transporte de roedores: ratones, ratas, hámsteres y cobayos, las superficies interiores de los confinamientos o encierros primarios que estén construidos de lámina, de fibra, de cartón o de plástico, deben estar recubiertas con malla y pantalla de alambre para evitar el escape de los animales.

7.2.1.2. Salida de emergencia.

Las aberturas de los confinamientos o encierros primarios deben estar fácilmente accesibles, en todo momento, para sacar rápidamente a los animales en caso de alguna contingencia.

7.2.1.3. Ventilación.

Aplicable al confinamiento o encierro primario que no se encuentra permanentemente fijo al área de carga para animales del vehículo de transporte

a) En caso de que las aberturas para la ventilación se encuentren en dos paredes laterales del confinamiento o encierro primario, éstas deben ser opuestas, y las aberturas representarán mínimo, la quinta parte del área de cada una de las paredes.

b) Si las aberturas para ventilación se localizan en las cuatro paredes del confinamiento o encierro primario, cada una de ellas deben constituir como mínimo la décima parte de la superficie total de cada pared.

c) Por lo menos un tercio de la superficie total mínima para ventilación debe estar localizada en la mitad superior del confinamiento o encierro primario y por lo menos otro tercio del área total mínima requerida para ventilación se localizará en la mitad inferior del confinamiento o encierro primario.
d) Los confinamientos o encierros primarios deben presentar características de diseño tales como rebordes, costillas u otros artefactos en las superficies exteriores de las paredes que presentan aberturas para ventilación, con el fin de evitar la obstrucción de estas aberturas y brindar un espacio mínimo de dos centímetros para la circulación del aire entre el confinamiento o encierro primario y la carga adyacente o bien la pared del vehículo.
7.2.1.4. Otras características de diseño.
El confinamiento o encierro primario debe presentar en su exterior agarraderas u otros artefactos que permitan levantarlo.
7.2.1.5. Confinamiento o encierro primario fijo con excepción de roedores.
a) Cuando el confinamiento o encierro primario se encuentra permanentemente fijo en el área de carga para animales del vehículo de transporte de tal forma que la abertura frontal es la única fuente de ventilación, ésta debe abrir hacia el exterior, hacia un corredor que no tenga obstrucciones o hacia un pasaje del vehículo de transporte.
b) La abertura frontal de ventilación debe ser de por lo menos el 90% de la superficie total de la pared frontal y estar cubierta con barrotes metálicos, tela de alambre o metal desplegado liso.
c) A los animales socialmente dependientes (por ejemplo: hermanos carnales, madres y otros miembros de grupos familiares) se les debe permitir contacto visual y olfativo.
d) Las hembras en calor o aquellas en que la presencia del macho les induce al coito, deben transportarse en ausencia de animales machos.
7.2.2. Consideraciones sobre la reclusión de los animales.
7.2.2.1. Espacio mínimo para los animales.
a) Los confinamientos o encierros primarios para la transportación de animales vivos deben ser lo suficientemente grandes para asegurar que cada uno de los animales ahí recluidos tenga espacio suficiente para voltearse libremente y para llevar a cabo los ajustes posturales normales.
b) En ciertas especies animales pueden restringirse los movimientos, cuando la libertad de acción constituya un peligro para ellos mismos, sus manejadores o bien para otras personas; con base en opiniones profesionalmente aceptables.
c) El área de piso mínimo y la altura que se debe brindar a cada una de las especies animales de laboratorio durante su reclusión en el confinamiento o encierro primario, así como el número máximo de animales será de acuerdo a lo establecido en el Reglamento para el transporte de animales vivos de la Asociación del Transporte Aéreo Internacional (IATA), cuando se trate de transportaciones de distancias largas. Si las distancias son cortas se podrán utilizar los confinamientos primarios para su transportación.
7.2.2.2. Limpieza y saneamiento.
a) Los confinamientos o encierros primarios para la transportación de animales deben tener piso sólido para evitar el goteo de los desechos y excrementos.
b) Los materiales y las características de construcción de los confinamientos o encierros primarios podrán ser tales que los hagan apropiados para emplearlos una sola vez (desechables). En el caso de que se usen más de una vez (permanentes) deben permitir entre cada uso la limpieza y saneamiento que establece la presente Norma.
c) Los confinamientos o encierros primarios deben contener un material de cama limpio, seguro, no tóxico y adecuado para absorber la humedad; en cantidad suficiente para mantener a los animales ahí recluidos limpios y secos.
7.2.2.3. Leyendas y documentación.
Los confinamientos o encierros primarios que no estén permanentemente fijos al área de carga para animales de los vehículos de transporte deben estar marcados claramente sobre la tapa y,

por lo menos, sobre la superficie exterior de una de las paredes laterales con las palabras "Animales Vivos" o "Animales Silvestres", según sea el caso. Las letras no serán menores de 2.5 centímetros de altura y la presencia de flechas u otras marcas deben indicar la posición vertical correcta del contenedor.

La documentación que acompaña al embarque de animales debe estar fija en la parte exterior del confinamiento o encierro primario y fácilmente accesible.

7.3. Vehículo de transporte.

Puede ser un vehículo motorizado, ferrocarril, aéreo o marítimo.

7.3.1. Espacio de carga para animales.

7.3.1.1. Seguridad y comodidad.

a) El espacio de carga para animales del vehículo de transporte empleado para trasladar animales debe estar diseñado y construido para preservar la salud y proteger la seguridad y comodidad, en todo momento de los animales vivos ahí encerrados.

b) El espacio de carga para animales debe estar construido y mantenido de tal manera, que evite la entrada de humos y gases generados en el motor del vehículo.

c) Los animales vivos no deben transportarse junto con ninguna sustancia, material o artefacto que pueda potencialmente afectar su salud y bienestar.

7.3.1.2. Ventilación y temperatura.

Se debe asegurar y, en su caso, tomar las precauciones necesarias, para evitar que los animales permanezcan a la intemperie o bien se expongan a temperaturas extremas que afecten su salud y bienestar.

7.3.1.3. Salida de emergencia.

Los confinamientos o encierros primarios deben colocarse en el espacio de carga para animales, de tal forma que en caso de emergencia puedan desalojarse lo más rápido posible.

7.3.1.4. Limpieza.

El interior del área de carga para animales del vehículo de transporte debe mantenerse limpio.

7.4. Provisión de agua y alimento.

7.4.1. Antes del embarque.

7.4.1.1. Se debe ofrecer agua potable a todos los animales dentro de las cuatro horas previas a ser transportados.

7.4.1.2. Los animales que así lo requieran tales como ratones, ratas, hámsteres y cobayos deben tener acceso al agua y al alimento todo el tiempo cuando la transportación exceda un periodo de seis horas. En estos casos el agua y el alimento, o una preparación que satisfaga los requerimientos de agua y alimento en calidad y cantidad suficientes para un periodo mínimo de 24 horas, debe colocarse dentro del confinamiento o encierro primario.

7.4.1.3. Ningún transportista o intermediario aceptará para traslado los animales señalados en el número anterior, sin la provisión adecuada dentro del confinamiento o encierro primario de agua y alimento; o una preparación que cumpla los requerimientos en cantidad de agua y alimento durante el transporte.

7.4.1.4. Los números 7.4.1.1. al 7.4.1.3. no tendrán cumplimiento cuando esté indicado por hibernación, prescripción médico veterinaria, ayuno normal o cualquier otra práctica profesionalmente aceptada.

7.4.2. Durante el transporte.

7.4.2.1. Los animales en tránsito con excepción de los mencionados en el punto 7.4.1.2. deben tener acceso al agua potable por lo menos cada 12 horas, desde el momento de la partida o desde que son aceptados para su transportación.

7.4.2.2. Los animales en tránsito con excepción de los mencionados en el punto 7.4.1.2. deben alimentarse adecuadamente por lo menos cada 24 horas.

7.4.2.3. Aquellos animales que con base en prácticas comúnmente aceptadas requieren agua y alimento con mayor frecuencia, debe satisfacerse este requerimiento.

7.4.2.4. Los números 7.4.1.1. al 7.4.1.3. no tendrán cumplimiento cuando esté indicado por hibernación, prescripción médico veterinaria, ayuno normal o cualquiera de otras prácticas profesionalmente aceptadas.
7.4.3. Indicaciones pertinentes a la provisión de agua y alimento.
7.4.3.1. Cuando sea necesario, el confinamiento o encierro primario para el traslado de animales vivos, debe llevar por escrito las instrucciones relativas a los requerimientos de agua y alimento.
7.5. Cuidados durante la transportación.
7.5.1. Inspección visual.
7.5.1.1. Transporte de superficie.
Durante la transportación de superficie será responsabilidad del chofer o de otros empleados inspeccionar visualmente a los animales con la frecuencia que indiquen las circunstancias, pero no menos de cada cuatro horas, para asegurarse de que: reciban suficiente aire para respirar normalmente; la temperatura ambiente esté dentro de los límites establecidos; que todas las disposiciones sean observadas para determinar si cualquier animal está en obvio sufrimiento físico y en caso necesario para brindarles atención médico veterinaria lo más pronto posible.
7.5.1.2. Transporte aéreo.
Cuando el espacio de carga para animales de la aeronave esté accesible, los animales deben observarse por lo menos cada cuatro horas, en caso contrario la inspección se realizará cada vez que la aeronave cargue o descargue o cuando por otra razón el espacio de carga para animales esté accesible, para realizar las verificaciones enlistadas en el inciso a) del numeral 7.5.1.1.
7.5.2. Seguridad.
7.5.2.1. Los animales silvestres o peligrosos no deben sacarse de su confinamiento o encierro primario excepto en condiciones de extrema urgencia.
Si se emplean confinamientos o encierros primarios temporales éstos deben ser lo suficientemente sólidos para evitar el escape de los animales.
7.5.2.2. Otros animales tampoco deben sacarse de su confinamiento o encierro primario a menos que se coloquen en otros confinamientos o encierros primarios que cumplan las disposiciones de la presente Norma.
7.5.2.3. Seguridad Animal: En el puerto de entrada los animales de laboratorio objeto de transporte no deben ser retirados de sus confinamientos o encierros primarios de origen, tampoco se debe alterar en forma alguna la integridad de sus contenedores, en virtud de que ello invalida la calidad microbiológica original de los sujetos, considerados genéricamente como libres de enfermedad como es el caso de los animales axénicos, gnotobióticos, de flora microbiana definida. El mismo tratamiento se le debe dar a los animales inmunodeficientes o inmunocomprometidos u otros similares.
7.6. Estaciones de carga.
7.6.1. Separación de animales y objetos.
7.6.1.1. Los transportistas e intermediarios no deben mezclar embarques de animales con carga inanimada.
7.6.2. Condiciones medio ambientales.
7.6.2.1. Ventilación.
En todas las áreas donde se alojan animales, se debe asegurar y, en su caso, tomar las precauciones necesarias, para evitar que los animales permanezcan a la intemperie o bien se expongan a temperaturas extremas que afecten su salud y bienestar.
7.7. Manejo de los animales.
7.7.1. Prontitud de las maniobras.
7.7.1.1. La movilización de los animales desde las áreas de carga hasta los vehículos de transporte y viceversa debe realizarse con el empleo de los artefactos apropiados y lo más rápido posible.

7.7.2. Protección contra las inclemencias del clima.
Se debe asegurar y, en su caso, tomar las precauciones necesarias, para evitar que los animales permanezcan a la intemperie o bien se expongan a temperaturas extremas que afecten su salud y bienestar.
7.7.3. Seguridad de los animales.
7.7.3.1. Se debe evitar el manejo brusco o descuidado de los confinamientos o encierros primarios, que pueda causar trauma físico o emocional a los animales.
7.7.3.2. El confinamiento o encierro primario para transporte de animales no debe ser sacudido, arrojado, tirado o inclinado innecesariamente ni tampoco apilado en forma que previsiblemente pueda caerse.
8. Técnicas experimentales
8.1. Analgesia y anestesia.
Cualquier procedimiento que cause mayor dolor o molestia en los animales, que la producida por inyección o marcaje en orejas, requerirá el uso de tranquilizantes, analgésicos o anestésicos. Si es necesario efectuar un procedimiento doloroso sin el uso de anestesia, analgésicos o tranquilizantes, porque su uso afectaría los resultados o propósitos del experimento, éste debe ser aprobado por el Comité.
La elección del fármaco a utilizar debe ser responsabilidad del investigador, quien puede consultar con el Médico Veterinario. Los signos que deben ser evaluados en la medida de lo posible en los animales anestesiados se señalan en el Apéndice C Informativo.
No se permitirá la utilización de relajantes musculares o drogas paralizantes del tipo de la succinilcolina, guayacolato de glicerol o curariformes ya que no son anestésicos. Sólo se podrán utilizar estas sustancias en combinación con analgésicos de efecto conocido en proyectos aprobados por el Comité.
La dosificación seleccionada dependerá primero de los efectos, ya sea por sedación, analgesia o anestesia y después con base en la ruta de administración (I.V., I.M., I.P., S.C., oral). La selección de la dosis dependerá de la especie, cepa o raza, edad, porcentaje de grasa en el cuerpo y condición del animal.
El animal debe de ser manejado siempre con cuidado pero con firmeza, procurando la seguridad del personal que lo manipula. Se debe evitar la lucha y el estrés en todo momento, ya que la excitación prolongada puede alterar la circulación y el estado metabólico del individuo e inducir un estado de choque.
Previo a la anestesia los animales deben someterse a un periodo de ayuno suficiente para vaciar estómago, con el fin de prevenir la regurgitación o aspiración del contenido gástrico.
Además de las consideraciones mencionadas en la elección del anestésico o analgésico, se deben contemplar las siguientes:
•Sustancias como la ketamina pueden utilizarse en combinación con agentes relajantes musculares que potencialicen su acción y optimicen su efecto como los tranquilizantes que producen analgesia y sedación.
•Sustancias como el droperidol no son recomendables para su administración intramuscular en cobayos, debido a que existe evidencia que indica que éste induce un efecto irritativo y de necrosis.
•No se permite en ninguna especie de laboratorio el uso de cloroformo, debido a su bajo margen de seguridad y a la grave toxicidad potencial que sugiere su uso, tanto para los humanos como para los animales.
•No se recomienda el uso del dietiléter por su alto grado explosivo. Se deben implantar medidas de seguridad que garanticen su manejo apropiado. Debido a la fuerte irritación que produce y propensión a producir bronco espasmos no se recomienda utilizar esta sustancia en conejos y roedores de laboratorio que padezcan problemas respiratorios clínicos y subclínicos, particularmente no se debe usar en cobayos y conejos de más de dos kilos de peso corporal por el estrés que produce.

8.1.1. Preanestésicos.

Se recomienda el uso, siempre que el procedimiento experimental lo permita de tranquilizantes, analgésicos y anticolinérgicos antes de suministrar el anestésico. Los preanestésicos, tranquilizantes y analgésicos ayudan a inducir la anestesia más suavemente, dado que reducen o eliminan la etapa de excitación. Los tranquilizantes reducen la aprensión del animal, lo cual facilita su inmovilización para la inyección intravenosa.

Los narcóticos y tranquilizantes que se administran como preanestésicos también reducen la cantidad general de la dosis de anestesia que se requiere y por lo tanto incrementan el margen de seguridad. La única desventaja del uso de estas drogas es que enmascaran las etapas y planos de la anestesia. Los colinérgicos, como la atropina, reducen las secreciones e inhiben el efecto de la estimulación vagal.

8.2. Administración de fluidos y sustancias.

La administración parenteral de fluidos y sustancias deberá hacerse conforme a las prácticas clínicas y científicas generalmente aceptadas en el animal sujeto o inmóvil. Si la técnica causa estrés o dolor, se usarán sedantes, analgésicos o anestésicos, a menos que esté contraindicado en el protocolo experimental, en cuyo caso deberá contar con la aprobación del Comité

CUADRO No. 9

VIAS DE ADMINISTRACION DE FLUIDOS Y SUSTANCIAS

ANIMAL	VENA AURICULAR	VENA CAUDAL	VENA CEFALICA	VENA FEMORAL	* VENA PENEANA	VENA SAFENA	* VENA SUBLINGUAL	* VENA YUGULAR
Rata	-	+	-	-	+	+	+	+
Ratón	-	+	-	-	-	-	-	-
Jerbo	-	-	-	-	-	-	+	-
Hámster	-	-	-	-	-	-	-	+
Cuyo	-	-	-	+	+	+	+	+
Conejo	+	-	-	-	-	-	-	-
Perro	-	-	+	+	-	+	+	+
Gato	-	-	+	+	-	+	+	+
Porcino	+	-	-	-	-	-	+	-
Primates no-humanos	-	-	+	+	-	+	+	+

*** Previa anestesia o sedación**
+ Permitido
- No permitido

8.2.1. Obtención de sangre

CUADRO No. 10

VIAS DE OBTENCIÓN DE SANGRE

ANIMAL	*CORAZON	SENO	VENA	VENA	* VENA

		ORBITAL	AURICULAR	CAUDAL	CAVA ANTERIOR
Rata	+	+	-	+	-
Ratón	+	+	-	+	-
Jerbo	+	+	-	-	-
Hámster	+	+	-	-	-
Cuyo	+	-	+	-	-
Conejo	+	-	+	-	-
Perro	+	-	-	-	-
Gato	+	-	-	-	-
Porcino	-	+	+	-	+
Primates no humanos	-	-	-	-	-

* Previa anestesia o sedación
+ Permitido
- No permitido

ANIMAL	* VENA CAVA POSTERIOR	VENA CEFALICA	VENA FEMORAL	VENA SAFENA	* VENA YUGULAR
Rata	-	-	+	-	-
Ratón	-	-	-	-	-
Jerbo	-	-	-	-	-
Hámster	-	-	-	-	-
Cuyo	+	-	-	-	-
Conejo	+	-	-	-	-
Perro	-	+	+	+	+
Gato	-	+	+	+	+
Porcino	-	+	+	-	+
Primates no humanos	-	+	+	+	+

* Previa anestesia o sedación
+ Permitido
- No permitido

9. Eutanasia

9.1. Objetivo.

Este capítulo tiene por objeto describir los procedimientos empleados para inducir de manera humanitaria la muerte de los animales (eutanasia) empleados en la investigación científica, desarrollo tecnológico e innovación, pruebas de laboratorio y enseñanza, con el propósito de eliminar o disminuir al mínimo el dolor y el estrés previo y durante el procedimiento; para lograrlo cualquier técnica aplicada debe causar en el animal: rápida inconsciencia, paro cardiaco y/o respiratorio y pérdida de la función cerebral. Además, debe reducir al mínimo la perturbación emocional, la incomodidad y/o el sufrimiento experimentado por la persona que lleve a cabo el procedimiento.

9.2. Consideraciones generales.

9.2.1. Concordancia con otras leyes.
Los métodos y agentes descritos deben aplicarse de acuerdo con las leyes federales y estatales aplicables que gobiernan la adquisición, posesión, comercio, transporte, prescripción médica, suministro, empleo, uso y consumo de fármacos y medicamentos; lo relativo a la salud ocupacional; y al impacto medio ambiental.
9.2.2. Selección de métodos.
No existe un solo método de eutanasia que se aplique a todas las especies y a todas las circunstancias. Los criterios para seleccionarlo deberán incluir: la compatibilidad con los propósitos del protocolo experimental; la capacidad de producir inconsciencia y muerte rápidamente; su confiabilidad; y su seguridad para el personal.
9.2.2.1. La responsabilidad de la selección del método de eutanasia recae en el investigador y/o el Médico Veterinario responsable.
En el caso de los métodos de eutanasia no mencionados en esta Norma es indispensable la aprobación del Comité, la cual estará basada en una justificación, escrita, de índole científica, presentada por el investigador.
9.2.2.2. Criterios de elección para métodos de eutanasia.
El método seleccionado para la eutanasia depende de varios factores, entre los que se destacan la naturaleza del estudio, la especie animal involucrada y su número. Dependiendo de la especie, el procedimiento debe ser individual. Sin importar el tipo de grupo animal destinado para eutanasia se establece que el procedimiento seleccionado debe cumplir invariablemente con lo siguiente:
Inducir la muerte sin producir signos de pánico o ansiedad en los sujetos.
Inducir la inconsciencia de los animales en un tiempo mínimo.
Ser un método confiable y reproducible.
Ser seguro para el personal involucrado en su uso.
Poseer compatibilidad con los requerimientos y el propósito del estudio.
Tener un impacto ambiental mínimo.
Ser a prueba de fallas.
Localizarse en un sitio apartado de los cuartos de animales.
9.2.2.3. La selección y aplicación del método deben hacerse de acuerdo a los más altos principios éticos y de conciencia social.
9.2.2.4. Cuando ocurran circunstancias imprevistas en esta Norma.
En el caso de especies exóticas o raras, animales nonatos o neonatos, u otros, la técnica de eutanasia debe ser seleccionada por un Médico Veterinario certificado de acuerdo a su juicio profesional y conocimientos de las técnicas clínicamente aceptables. Sin embargo, la autoridad de aprobación de estos métodos al igual que de aquellos clasificados en esta Norma como aceptables condicionalmente e inadmisibles estará en manos del Comité; siendo esenciales en la aplicación del criterio profesional el tamaño del animal y las características fisiológicas y conductuales específicas de la especie.
9.2.3. Aplicación de la técnica.
9.2.3.1. Cuando el forcejeo durante la captura o la inmovilización cause dolor, lesiones o ansiedad en el animal o peligro para el operario, debe considerarse el uso de tranquilizantes, analgésicos y/o fármacos inmovilizantes.
9.2.3.2. Cuando se lleve a cabo la eutanasia no deben estar presentes otros animales, con excepción de grupos que mueran al mismo tiempo.
9.2.3.3. En algunas especies la inmovilidad no debe interpretarse como inconsciencia.
9.2.3.4. En la eutanasia de perros y gatos, el trato debe ser benévolo, de preferencia en un medio ambiente familiar, el manejo cuidadoso y se sugiere hablarles durante el procedimiento.
9.2.3.5. Después de aplicar la eutanasia, es imperativo verificar la muerte del animal confirmando la cesación de los signos vitales y otros que, a juicio profesional, se tomen en consideración de acuerdo a la especie y al método de eutanasia empleado.

9.2.3.6. Cuando se emplean fármacos eutanásicos que causan la muerte por hipoxia directa o indirecta, la inconsciencia debe preceder a la pérdida de actividad motriz.
9.2.3.7. Cuando el método de eutanasia causa una interrupción física de la actividad cerebral por conmoción, destrucción directa del cerebro o despolarización eléctrica de las neuronas se induce inconsciencia, pero se debe causar la muerte del animal por destrucción del tallo cerebral donde se localizan los centros de control de la actividad respiratoria y cardiaca.
9.3. Consideraciones sobre el personal
9.3.1. Capacitación
9.3.1.1. El personal que lleva a cabo la eutanasia debe tener entrenamiento y/o experiencia comprobable en la técnica que se aplicará. El entrenamiento y experiencia deben incluir: conocimiento de la conducta normal de la especie que va a ser sometida a la eutanasia y de cómo afecta ésta el manejo y la inmovilización y comprensión del mecanismo por el cual la técnica seleccionada induce la inconsciencia y la muerte.
9.3.1.2. Antes de otorgarse la responsabilidad total de realizar la eutanasia, todo el personal debe haber demostrado capacidad y habilidad en el uso de la técnica, bajo estricta supervisión.
9.3.2 Del personal encargado de la eutanasia.
9.3.2.1. Se debe mantener una rotación continua del personal directamente involucrado en la eutanasia.
9.3.2.2. Los bioterios deben tener programas de entrenamiento que permitan al personal desempeñar sus labores competentemente.
9.3.2.3. Deben contar con supervisión y apoyo durante el desempeño de su trabajo.
9.4. Métodos recomendables**.**
9.4.1. Agentes inhalables.
9.4.1.1. Generalidades:
La conveniencia de cada uno de estos agentes depende de la brevedad entre el momento en que se aplica y el momento en que se induce la inconsciencia. Se recomienda que el animal se exponga rápidamente a altas concentraciones del fármaco.
9.4.1.1.1. Los aparatos empleados para suministrar y mantener altas concentraciones de agentes que deben estar en buenas condiciones de operación.
9.4.1.1.2. Se deben tomar las precauciones necesarias para reducir al mínimo los riesgos que la exposición aguda y/o crónica a la mayoría de estas sustancias representan para la salud del hombre, por ejemplo, el éter explosiones, el halotano narcosis, el nitrógeno y el monóxido de carbono hipoxemia y el óxido nitroso adicción.
9.4.1.1.3. Se debe tomar en cuenta que los animales neonatos son más resistentes a la hipoxemia que los adultos de la misma especie.
9.4.1.1.4. Las cámaras de eutanasia deben mantenerse limpias y libres de olores.
9.4.1.1.5. En la cámara de eutanasia sólo se colocarán animales de la misma especie, en número adecuado.
9.4.1.2. Anestésicos.
Los anestésicos inhalables recomendados para la eutanasia de animales que pesan menos de 7 kg, o en aquellos en que la venipuntura es difícil son, en orden de preferencia: halotano, enflurano, isoflurano, metoxiflurano y éter, solos o en combinación con el óxido nitroso. Este puede combinarse con otros inhalables, para acelerar el inicio de la anestesia, pero por sí mismo no induce anestesia en animales, por lo cual no debe usarse solo.
El animal se coloca en un receptáculo cerrado que contenga un algodón o gasa empapada en el anestésico. El anestésico también puede introducirse mediante un vaporizador, pero requiere un mayor tiempo de inducción. Los vapores se inhalan hasta que cesa la respiración y ocurre la muerte; debe haber suficiente oxígeno para evitar la hipoxemia.
La piel del animal nunca debe estar en contacto directo con estos compuestos pudiendo colocarse una rejilla o piso perforado entre el animal y el algodón o gasa con anestésico.

9.4.1.2.1. El éter es el anestésico volátil menos recomendable por ser altamente inflamable y explosivo, representa un grave riesgo para el personal. Además es irritante para ojos y nariz. El éter sólo se puede usar dentro de una campana ventilada o en un área bien ventilada y en ausencia de flamas u otras fuentes de ignición, se deben colocar en lugar visible advertencias de que hay éter presente o en uso, los cadáveres de los animales en los que se utilizó éter deben guardarse en refrigeradores o congeladores a prueba de explosiones y antes de hacerlo se permitirá que el químico se volatilice, también se debe permitir su volatilización antes de incinerarlos.
Las concentraciones en el aire a las que se pueden exponer las personas son: de éter, halotano, metoxiflurano, enflurano e isoflurano, menores que 2 p.p.m., y de óxido nitroso menos que 25 p.p.m.
9.4.1.3. Bióxido de carbono (CO2).
9.4.1.3.1. Es el método más recomendable para la eutanasia de varias especies de mamíferos tales como: ratas, ratones, cobayos, perros, hámsteres, conejos y gatos debido a su rápido efecto depresivo y anestésico que conduce a la muerte por hipoxia en pocos minutos, a que está fácilmente disponible en cilindros de gas comprimido, a que es barato, no inflamable, no explosivo, seguro de operación para el personal, no se acumula en los tejidos y no deforma la arquitectura celular.
9.4.1.3.2. Se debe considerar que los animales neonatos son más resistentes a la hipoxia y que algunas otras especies son extraordinariamente tolerantes al CO2.
9.4.1.3.3. Sólo se permite que la fuente de CO2 sean cilindros de gas comprimido liberado en una cámara cerrada; es preferible que la cámara se gasifique, previo a la introducción de los animales a una concentración mínima de 70%. Con el animal en la cámara, el flujo de CO2 debe reemplazar por lo menos el 20% del volumen total de aire por minuto.
9.4.2. Fármacos no inhalables.
9.4.2.1. Generalidades.
9.4.2.1.1. La administración intravenosa de fármacos que causan la muerte es el método más rápido y confiable de llevar a cabo la eutanasia. El personal debe poseer la habilidad para realizarlo y el animal debe estar inmovilizado.
9.4.2.1.2. Es el método más recomendable siempre y cuando no cause miedo o estrés al animal. Los animales asustados, deben sedarse antes de administrar el agente eutanásico.
9.4.2.1.3. Es aceptable la administración intraperitoneal cuando la vía intravenosa resulte impráctica o imposible, por ejemplo, animales que pesan 7 kg o menos, siempre y cuando el fármaco no sea irritante ni contenga bloqueadores neuromusculares.
9.4.2.1.4. Sólo se permite la inyección intracardiaca en animales bajo sedación profunda, anestesiados o comatosos.
9.4.2.1.5. No se permite la administración intramuscular, subcutánea, intratorácica, intrapulmonar, intrarenal, intraesplénica, intratecal o cualquier otra inyección no intravascular de los agentes eutanásicos.
9.4.2.1.6. Cuando el fármaco se administra por una vía diferente a la endovenosa, el animal debe colocarse en un lugar tranquilo que reduzca la excitación de las primeras etapas de la anestesia, y también la posibilidad de que se lesione.
9.4.2.2. Derivados del ácido barbitúrico.
9.4.2.2.1. Todos los barbitúricos son recomendados para la eutanasia, ya que inducen inconsciencia con sólo el dolor mínimo o transitorio de la venipuntura. Los más deseables son los más potentes, de más larga acción, estables en solución y baratos, tales como el pentobarbital sódico y el secobarbital.
9.4.2.2.2. La velocidad de acción de los barbitúricos depende de la dosis, la concentración, velocidad y vía de administración, las cuales deben combinarse para inducir la eutanasia suavemente y con mínima incomodidad para el animal. La dosis de pentobarbital sódico administrado por vía intravenosa es de 90 a 210 mg/kg, para la mayoría de las especies; ratas

y ratones requieren una dosis mayor (120 a 210 mg/kg). La dosis de secobarbital sódico es de 88 mg/kg.
9.4.2.2.3. La inyección intravenosa de los derivados del ácido barbitúrico es el método de elección para la eutanasia de perros y gatos, otros animales pequeños y los caballos.
9.4.2.2.4. Para los efectos de la Ley General de Salud, el pentobarbital sódico y el secobarbital se consideran sustancias psicotrópicas del grupo II, y por tanto, quedan sujetas en lo conducente a las disposiciones del capítulo V, título decimosegundo de la ley referida.
9.4.3. Métodos físicos.
9.4.3.1. Irradiación con microondas.
9.4.3.1.1. Los aparatos empleados para este procedimiento son completamente diferentes de los hornos domésticos tanto en kilowatts y megahertz, así como en la forma de aplicación de la energía al animal. Está absolutamente prohibido el uso de aparatos domésticos.
9.4.3.1.2. Sólo es aplicable a ratas y ratones, para fijar *in vivo* los metabolitos cerebrales, manteniendo la integridad anatómica del cerebro.
9.4.3.1.3. Se debe lograr la inconsciencia en menos de 100 mseg, y la muerte en menos de un segundo.
9.5. Métodos aceptables condicionalmente.
Para la valoración del método de eutanasia se recomienda, como referencia primaria, el reporte de la American Veterinary Medical Association (edición 1993 y posteriores) que los clasifica en aceptables, aceptables condicionalmente e inaceptables.
9.5.1. Generalidades.
Es indispensable que el investigador obtenga la aprobación del Comité, para la aplicación de los métodos mencionados en este punto, ofreciendo una justificación científica de su selección; habrá circunstancias en que se deban considerar justificaciones no-científicas. Las condicionales que hacen a un método o agente aceptable varían según la circunstancia, influyendo diversas consideraciones tales como: la seguridad del operario, la idoneidad para la especie animal, el impacto psicológico sobre las personas y la capacitación y experiencia del personal.
9.5.2. Agentes inhalables.
9.5.2.1. Nitrógeno y Argón (N2 y Ar).
9.5.2.2. Causan inconsciencia precedida de hipoxemia e hiperventilación y muerte sin dolor en perros, gatos y pollos. No debe usarse en ratas porque les causa estrés.
9.5.2.3. Esta técnica sólo se aplicará cuando se logren rápidamente concentraciones de oxígeno menores al 2%, y el animal esté bajo profunda sedación o anestesia; la muerte puede requerir de exposiciones prolongadas a estos gases.
9.5.3. Métodos físicos.
9.5.3.1. Generalidades.
9.5.3.1.1. Los métodos físicos de eutanasia que se incluyen son: dislocación cervical, decapitación, perno cautivo penetrante y electrocución y se pueden aplicar en las siguientes circunstancias: en animales pequeños de fácil manejo y con características anatómicas compatibles con el método seleccionado, en animales grandes de granja, zoológico o silvestres y cuando otros métodos puedan invalidar los resultados experimentales o interferir con el uso posterior de tejidos o fluidos corporales.
9.5.3.1.2. El personal que ejecute los métodos físicos debe poseer la habilidad y experiencia suficiente para no dejar al animal consciente y lesionado.
9.5.3.1.3. El personal debe ser capacitado en la aplicación de estas técnicas, empleando cadáveres o animales anestesiados.
9.5.3.1.4. Los métodos físicos de eutanasia sólo se aplicarán cuando los métodos recomendados hayan sido excluidos por alguna justificación científica o clínica y de preferencia en animales inconscientes o sedados.
9.5.3.2. Dislocación cervical.

9.5.3.2.1. La dislocación cervical manual ejecutada apropiadamente induce inconsciencia rápidamente, se aplica porque no contamina los tejidos con substancias químicas. Se acepta su aplicación en: ratones, ratas que pesen menos de 200 gr y conejos que pesen menos de 1 kg.
9.5.3.2.2. En la rata y ratón, se toma al animal por la base de la cola con una mano para su acomodo y se coloca sobre una superficie donde el animal se sostenga, con los dedos índice y pulgar de la otra mano o bien en su defecto un instrumento delgado pero rígido, se colocan sobre la base del cráneo y se ejerce tracción hacia atrás del animal a través de la base de la cola, para ocasionar la dislocación cervical.
9.5.3.2.3. En el conejo se lleva a cabo inicialmente colocando al animal sobre una superficie lisa y con una mano se toman y dirigen las patas traseras hacia atrás, con los dedos índice, medio, anular y meñique de la otra mano se colocan sobre la base del cráneo y el dedo pulgar es colocando sobre el maxilar inferior, acto seguido se procede a levantarlo y llevar a cabo la hiperextensión del animal, al ejercer tracción de los miembros posteriores hacia atrás, al tiempo de que la cabeza es detenida y se le da un pequeño giro para ocasionar la dislocación cervical.
9.5.3.2.4. Se acepta el uso de dislocadores mecánicos.
9.5.3.3. Decapitación.
La decapitación es una técnica para la eutanasia de roedores y conejos pequeños, que se realiza con un aparato especialmente diseñado para este propósito llamado guillotina. Es un medio para obtener tejidos y fluidos corporales libre de contaminación química y/o tejido cerebral íntegro.
9.5.3.4. Perno cautivo penetrante.
9.5.3.4.1. Cuando no se pueden emplear sustancias químicas, éste es un método práctico de eutanasia, en circunstancias excepcionales, previa justificación y aprobación del Comité.
9.5.3.4.2. Se recomienda el uso conjunto de otro método como la exsanguinación.
9.5.3.4.3. El perno cautivo penetrante es accionado por aire comprimido o por un cartucho de pólvora y su modo de acción es por contusión y trauma del hemisferio y tallo cerebral; por lo tanto, la colocación correcta del perno en la cabeza del animal es crítica. Además, el animal debe sujetarse apropiadamente como se indica en la Norma Oficial Mexicana NOM-033-ZOO-1995.
9.5.3.5. Electrocución.
9.5.3.5.1. La electrocución con corriente alterna es un método de eutanasia que se puede emplear en varias especies animales. Es imperativo que el animal esté inconsciente antes de ser electrocutado.
9.5.3.5.2. La eutanasia por electrocución es aceptable, sólo si se hace pasar a través del cerebro suficiente corriente eléctrica para inducir inconsciencia seguida de fibrilación cardiaca, hipoxia cerebral y muerte.
9.5.3.5.3. Se requieren habilidades y equipo especial para llevar a cabo este método. No se permite la aplicación de la corriente eléctrica de cabeza a cola o de cabeza a patas.
9.5.3.5.4. No se debe aplicar en animales cuyo peso corporal sea menor a 5 kg, porque la fibrilación ventricular y el colapso circulatorio pueden ser reversibles.
9.5.3.5.5. Se deben reconocer y prevenir los riesgos que este método involucra para los operarios.
9.5.3.6. Exanguinación: Sólo se puede llevar a cabo en animales bajo profunda sedación, anestesia o conmoción.
9.5.3.7. Descompresión: Sólo se puede aplicar en una cámara de descompresión, no se debe aplicar en animales inmaduros, puede ser reversible y puede causar timpanitis, hemorragias, vómito, convulsiones, micción y defecación.
9.5.3.8. Congelamiento instantáneo: Sólo se permite si el animal está anestesiado.
9.6. Métodos inadmisibles.
9.6.1. Generalidades.

Estos métodos sólo deben considerarse bajo circunstancias extraordinarias, se debe ofrecer una justificación científica o de otra índole convincente y deben ser aprobados por el Comité. Su uso es esporádico y solamente se permite cuando no haya otra opción.

9.6.2. Métodos físicos y químicos.

Está restringida la aplicación de los siguientes métodos por no considerarse humanitarios:

9.6.3. La administración parenteral de hidrato de cloral en perros, gatos y otros animales pequeños es inadmisible, debido a que los efectos secundarios tales como: jadeos, espasmos musculares y vocalizaciones pueden ser severos y su efecto sobre las personas indeseable.

9.6.4. Está prohibido el empleo, como único agente eutanásico, de sustancias que inducen parálisis muscular sin causar inconsciencia.

Estas sustancias son: El curare, succinil colina, galamina, estricnina, sales de magnesio y potasio, pancuronio, decametonio, vecuronio, atracurio, pipecuronio y doxacurio.

9.6.5. El empleo de la nicotina, como único agente eutanásico es inadmisible, sólo se podrá aplicar en animales anestesiados o inconscientes por otro medio.

9.7. Prohibiciones.

9.7.1. Está prohibido el uso de los siguientes métodos y sustancias para la eutanasia, debido al riesgo inherente para los operarios:

9.7.1.1. El cloroformo porque es hepatotóxico y probablemente cancerígeno y el cianuro porque es un veneno de extremo peligro para los seres humanos.

9.7.1.2. Las mujeres embarazadas no deben aplicar agentes anestésicos inhalables ya que la exposición a cantidades traza de estas sustancias en los primeros estadios del embarazo se ha asociado con abortos espontáneos y anormalidades congénitas.

9.7.2. Por consideración ética, no se consideran métodos humanitarios y, por tanto, se prohíbe el uso de los siguientes:

9.7.2.1. La contusión en la cabeza, aun cuando pueda causar inconsciencia.

9.7.2.2. El ahogamiento del animal, ya sea apretándole la garganta o sumergiéndolo en el agua.

9.7.2.3. El embolismo gaseoso, provocado por la inyección intravascular de aire.

9.7.2.4. El envenenamiento con o la administración de estricnina.

9.7.2.5. Los agentes que causan la muerte por hipoxia directa o indirecta, induciendo convulsiones antes de la inconsciencia.

9.8. Cuadros sinópticos.

CUADRO No. 11

APLICACION DE LOS AGENTES Y METODOS DE EUTANASIA

ANIMALES	METODOS RECOMENDADOS	METODOS ACEPTADOS CONDICIONALMENTE
Roedores y otros animales pequeños	Anestésicos inhalables, CO_2, Ar, N_2 irradiación con microondas, barbitúricos	N_2, Ar, dislocación cervical, decapitación
Conejos	Anestésicos inhalables CO_2, barbitúricos	N_2, Ar, dislocación cervical, decapitación, perno cautivo penetrante
Perros	Anestésicos inhalables, CO_2, barbitúricos	N_2, Ar, electrocución, perno cautivo penetrante
Gatos	Anestésicos inhalables, CO_2, barbitúricos	N_2, Ar
Primates	Barbitúricos	Anestésicos inhalables, CO_2, N_2, Ar

Porcinos	CO_2	Pinzas eléctricas seguido de sangrado inmediato.<br>Pistola de perno cautivo, seguido de sangrado inmediato (se deben utilizar cartuchos apropiados).
Todas las especies	____________________	Exanguinación

CUADRO No. 12

AGENTES Y METODOS DE EUTANASIA PROHIBIDOS

AGENTE	COMENTARIO
Descompresión	No es un método aceptado porque puede ocurrir la recompresión, muchas cámaras no son apropiadas, los animales inmaduros requieren prolongadas exposiciones y puede causar efectos desagradables en los observadores.
Congelamiento instantáneo	No se considera humanitario cuando se usa como único método. Sólo se acepta en animales anestesiados.
Embolismo gaseoso	Sólo se permite en animales anestesiados ya que puede estar acompañado de convulsiones, opistótonos y vocalizaciones.
Ahogamiento	No se considera humanitario; no se acepta.
Estricnina	No se acepta porque causa convulsiones violentas y dolorosas contracciones musculares.
Agentes curariformes, Sulfato de Magnesio, Clorato de Potasio y Nicotina	No son aceptables porque no causan inconsciencia antes de la muerte, la cual ocurre por asfixia.
Cloroformo	No se acepta por el riesgo que implica para las personas, es hepatotóxico y probablemente cancerígeno.
Cianuro	No se acepta por el sumo peligro que representa, además la forma en que muere el animal causa un efecto desagradable en los observadores.
Contusión	Aun cuando puede causar inconsciencia en el animal, no se considera un método de eutanasia.

10. Medidas de bioseguridad y salud ocupacional para el personal involucrado con la producción, cuidado y uso de los animales de laboratorio

10.1. Normas generales.

10.1.1. Cada institución de investigación que utilice animales de laboratorio establecerá las medidas específicas pertinentes de bioseguridad y salud ocupacional de acuerdo con su situación particular determinada por su Comité de Bioseguridad, su Comité Interno de Cuidado y Uso de Animales de Laboratorio y su área administrativa encargada de las relaciones laborales.

10.1.2. En el establecimiento de dichas medidas específicas se deben contemplar las consideraciones de la presente Norma además de las determinadas en forma interna de acuerdo con sus propias necesidades. Dichas medidas deben quedar plasmadas en un Manual específico de bioseguridad y salud ocupacional para Unidades de Cuidado y Uso de Animales de Laboratorio.

10.1.3. Es responsabilidad de los directivos de cada institución vigilar que se cumplan las disposiciones de bioseguridad y salud ocupacional.
10.2. Capacitación de personal.
10.2.1. El personal expuesto a riesgo debe ser capacitado debidamente. Dicha capacitación debe incluir un conocimiento de los procedimientos rutinarios de su trabajo, entendimiento claro de los riesgos a los que están expuestos y saber implementar las salvaguardas requeridas para su protección.
10.2.2. El manual específico de bioseguridad y salud ocupacional, debe señalar el tipo de capacitación del personal requerido para cada caso, como un elemento de cumplimiento obligatorio y debe indicar el tipo de entrenamiento relacionado con los diferentes agentes físicos, químicos y biológicos, así como las diversas zoonosis, alergias y condiciones especiales dadas por los experimentos a los que puedan estar expuestos.
10.2.3. Se debe capacitar adecuadamente en cuanto al manejo de productos de desecho, higiene personal y medicina preventiva en su área de trabajo.
10.3. Instalaciones y equipamiento.
10.3.1. Las instalaciones y el equipamiento relacionado con las medidas de seguridad laboral variarán de acuerdo con las características propias de cada institución, con el tipo de animales mantenidos y utilizados y con los proyectos específicos de investigación desarrollados.
10.3.2. Las unidades de cuidado animal como mínimo, deben contar con equipo apropiado para lavado de manos.
10.3.3. En el uso de sustancias químicas el personal debe tener conocimiento de las lesiones que pueden producir. Dichas sustancias deben ser manejadas cuidadosamente y almacenadas lejos de donde se encuentran los alimentos y material para la preparación de la cama de los animales.
10.3.4. Los productos volátiles deben ser colocados en áreas bien ventiladas o gabinetes especiales.
10.3.5. Todas las sustancias químicas deben estar propiamente etiquetadas y ser almacenadas en un gabinete especialmente designado para tal fin.
10.3.6. Se debe designar un área específica de la unidad de cuidado animal para el manejo de agentes potencialmente dañinos. Esta área debe estar separada del resto del albergue animal, así como de las áreas de apoyo.
10.3.7. Se debe seleccionar el confinamiento o encierro primario más apropiado de acuerdo con las recomendaciones de esta Norma, los animales utilizados, la naturaleza de los proyectos de investigación y el tipo de agentes potencialmente dañinos a los que pudiera haber exposición. En todo momento, confinamiento o encierro primario debe permitir el manejo controlado de cama, excretas y alimento contaminado.
10.3.8. El acceso a las diversas instalaciones de la unidad de cuidado animal estará restringido al personal autorizado.
10.4. Medidas generales de protección e higiene personal.
10.4.1. Es requisito indispensable que todo el personal mantenga un alto grado de limpieza personal en todo momento.
10.4.2. El personal debe lavarse las manos y cambiarse de ropa con la frecuencia necesaria para mantener constante su limpieza e higiene personal.
10.4.3. La práctica de baño antes y/o después de la jornada laboral por parte del personal, estará determinada con base en las necesidades establecidas por el bioterio, de acuerdo con el criterio del Médico Veterinario responsable.
10.4.4. La ropa de trabajo debe ser específica para su uso en el área de los animales y debe ser provista y lavada por la institución. Cuando sea apropiado se realizará la descontaminación de la ropa utilizada antes de someterla a lavado.

10.4.5. La utilización de guantes desechables, cubrebocas, gorros, batas, overoles, botas o cubiertas para zapato es deseable y dependerá de las circunstancias especiales de cada unidad.
10.4.6. El uso de prendas externas como batas, overoles, botas, gorros, que se utilicen dentro del área de mantenimiento, no deben ser utilizadas fuera de esta área.
10.4.7. La ingestión de comida o líquidos, así como el uso de tabaco y la aplicación de maquillaje dentro del área de los animales estará prohibida. Puede haber un área especialmente diseñada para tal fin en la unidad.
10.4.8. Todo personal potencialmente expuesto a agentes nocivos conocidos debe ser provisto del equipo de protección personal apropiado al agente en cuestión y observar lo señalado en el punto 10.7.1.
10.4.9. Deben existir contenedores especiales para manejo de material cortante.
10.5. Evaluación médica y medidas de medicina preventiva.
10.5.1. Toda institución que maneje animales de laboratorio debe implantar un programa de evaluación médica y medicina preventiva para el personal, que debe ser incluido en el Manual Específico de Bioseguridad y Salud Ocupacional para Unidades de Cuidado y Uso de Animales de Laboratorio.
10.5.2. En la unidad de manejo de animales de laboratorio se debe llevar un expediente de cada uno de sus empleados.
10.5.3. Todo personal de nuevo ingreso será sometido a evaluación médica con el objeto de determinar riesgos potenciales individuales. Esta evaluación incluirá la realización de biometría hemática completa, química sanguínea, exámenes general de orina y coproparasitoscópico, aplicación de PPD y radiografía de tórax.
Otros exámenes de laboratorio y gabinete se realizarán de acuerdo al caso especial y a las características específicas de la unidad de cuidado animal.
10.5.4. Todo el personal será sometido a evaluación médica periódica de acuerdo a las circunstancias particulares de la unidad de cuidado animal.
10.5.5. La obtención de muestras de suero de referencia se hará sólo en circunstancias determinadas de acuerdo con el Comité de Bioseguridad, el Comité Interno de Cuidado y Uso de Animales de Laboratorio y el área administrativa encargada de las relaciones laborales de cada institución.
10.5.6. El programa de evaluación médica y medicina preventiva debe incluir vigilancia de las diferentes zoonosis a las que pueda estar expuesto el personal de acuerdo con el Apéndice B (Informativo).
10.5.7. Se establecerá un sistema de reporte, registro y seguimiento de toda lesión que sufra el personal por parte de algún animal. Dependiendo de la especie y del riesgo se tomarán las medidas pertinentes.
10.5.8. En heridas sufridas por mordedura o rasguño directo de perros, gatos o animales potencialmente infectados con rabia, se debe proceder a aislar al animal para observación por 10 días. En caso de muerte del animal durante el periodo de observación se debe proceder al diagnóstico de rabia.
10.5.9. En caso de riesgo a exposición a virus de hepatitis A (manejo de monos), se debe ofrecer un esquema de inmunización pre-exposición de acuerdo con las vacunas existentes.
10.5.10. En caso de riesgo de exposición a tuberculosis en el trabajo con primates no humanos, se debe repetir el examen de PPD cada año, siempre que el resultado inicial hubiera sido negativo; en caso de que el resultado sea positivo, debe ser reportado al jefe inmediato superior para tomar las medidas necesarias.
10.6. Disposición final de productos biológicos, excretas y cadáveres.
10.6.1 El manejo con respecto a la disposición final de productos biológicos, excretas y cadáveres será determinado de acuerdo con lo establecido en la NOM-087-ECOL-1995 y cualquier otra disposición aplicable en la materia.

10.6.2. Todos los animales de laboratorio, excretas, productos biológicos, alimentos y cama que hayan sido expuestos a radiaciones ionizantes deben ser manejados y dispuestos de acuerdo a las recomendaciones específicas del Comité de Bioseguridad de la institución y dentro de los lineamientos de la Comisión Nacional de Energía Nuclear.
10.7. Supervisión.
10.7.1. Los puntos considerados como medidas de bioseguridad del personal referido en la presente Norma deben quedar registrados apropiadamente en la bitácora y en el expediente de cada trabajador.
10.7.2. La supervisión de las medidas de bioseguridad debe ser parte de las funciones del Comité de Bioseguridad, así como del Comité Interno para el Cuidado y Uso de Animales de Laboratorio.
11. Verificación
11.1. Son objeto de regulación por esta Norma, todas las personas físicas o morales que manejen animales de laboratorio para reproducción, crianza, manutención, venta, distribución, transporte y/o usos en investigación científica, desarrollo tecnológico e innovación, pruebas y enseñanza.
11.2. Todo organismo, institución o individuo que aloje, produzca, utilice o distribuya animales de laboratorio con fines de investigación científica, desarrollo tecnológico e innovación, pruebas y enseñanza, debe dar aviso de inicio de funcionamiento a la Secretaría a través de la CONASAG y entregar un informe anual de actividades.
11.3. Dicho aviso de inicio de funcionamiento debe indicar la actividad primordial a la que se dedique cada bioterio y/o establecimiento, de acuerdo con la siguiente clasificación:
A) Producción: reproducción, crianza, manutención o distribución.
B) Experimentación (uso): investigación científica, desarrollo tecnológico e innovación, pruebas de laboratorio y enseñanza.
C) Mixto: Incluye la combinación de los incisos A y B.
11.4. Todos los bioterios y/o establecimientos independientemente de su tipo, tienen que designar a un Médico Veterinario responsable.
El cumplimiento de las especificaciones establecidas en esta Norma, será verificado por personal oficial de la Secretaría o por las Unidades de Verificación aprobadas que ésta designe, a través del Responsable Administrativo y/o el Médico Veterinario responsable del bioterio y/o establecimiento.
11.5. Para los bioterios y/o establecimientos de producción, es motivo de verificación:
a) El aviso de inicio de funcionamiento ante la Secretaría otorgado a través de la CONASAG.
b) La existencia de un Médico Veterinario responsable.
c) Las instalaciones destinadas para el alojamiento de los animales de laboratorio, así como el perfil del personal que interviene en su cuidado diario.
d) El registro de las especies alojadas.
e) El registro de las rutinas de trabajo.
f) Certificado de salud y calidad de los animales.
g) Los registros de control de atención médica veterinaria.
h) El registro del total de animales producidos por año.
i) Los registros de distribución de los animales.
j) El registro de adquisición, por compra o donación, de animales por año.
k) Los vehículos utilizados para el transporte de animales de laboratorio.
l) Los procedimientos para la movilización de los mismos.
m) El registro de saneamiento de los vehículos.
n) El programa permanente de capacitación para el personal.
o) El Manual de Organización y Procedimientos del Comité Interno para el Cuidado y Uso de los Animales de Laboratorio.

11.6. Para los bioterios y/o establecimientos clasificados como de experimentación, es objeto de verificación:
a) Aviso de inicio de funcionamiento ante la Secretaría otorgado a través de la CONASAG.
b) La existencia de un Médico Veterinario responsable.
c) Las instalaciones destinadas para el alojamiento de los animales de laboratorio, así como del perfil del personal que interviene en su cuidado diario.
d) El registro de las especies alojadas.
e) El certificado de salud y calidad de los animales.
f) El registro de adquisición, por compra o donación, de animales por año.
g) El registro del total de animales utilizados por año.
h) Los informes anuales conforme a lo establecido en los Apéndices B (Normativo) y A (Informativo).
i) El Manual de Organización y Procedimientos del Comité Interno para el Cuidado y Uso de los Animales de Laboratorio.
j) El Manual de Bioseguridad y el Programa de Salud Ocupacional.
k) Un programa permanente de capacitación para el personal.
11.7. Para los bioterios y/o establecimientos mixtos, es motivo de verificación:
a) El aviso de inicio de funcionamiento ante la Secretaría otorgado a través de la CONASAG.
b) La existencia de un Médico Veterinario responsable.
c) Las instalaciones destinadas para el alojamiento de los animales de laboratorio, así como el perfil del personal que interviene en su cuidado diario.
d) El registro de las especies alojadas.
e) Registros de control de atención médica veterinaria.
f) El registro de las rutinas de trabajo.
g) En su caso, registro de adquisición por compra o donación de animales por año y certificado de salud y calidad.
h) El registro del total de animales producidos y/o utilizados por año.
i) Los informes anuales conforme a lo establecido en los Apéndices B (Normativo) y A (Informativo).
j) Manual de Organización y Procedimientos del Comité Interno para el Cuidado y Uso de los Animales de Laboratorio.
k) Manual de Bioseguridad y el Programa de Salud Ocupacional.
l) Los vehículos utilizados para el transporte de animales de laboratorio.
m) Los procedimientos para la movilización de los mismos.
n) El registro de saneamiento de los vehículos.
o) Un programa permanente de capacitación para el personal.

12. Sanciones

El incumplimiento a las disposiciones contenidas en la presente Norma será sancionado conforme a lo establecido por la Ley Federal de Sanidad Animal y la Ley Federal sobre Metrología y Normalización.

13. Concordancia con normas internacionales

Esta Norma no coincide con norma internacional alguna, por no existir referencia al momento de su elaboración.

14. Bibliografía

1.AALAS. Manual para Tecnólogo en Animales de Laboratorio, Córdoba, TN, 1998.
2.CONEVET/CENEVAL. Guía para la Certificación en el Ejercicio Específico de la Medicina Veterinaria y Zootecnia en Ciencia de los Animales de Laboratorio. México, D.F., 2000.
3.Aademia Nacional de Medicina, Academia Mexicana de Ciencias y la Universidad Nacional Autónoma de México. Guía para el Cuidado y Uso de los Animales de Labortorio. México, D.F. 1999 Traducción autorizada de la versión en inglés “Guide for the Care and Use of Laboratory”. Animals. National Academy of Sciences. National Academy Press. Washington, D.C., 1996.

4.Acha, P. and Szyfres B. Zoonosis and Communicable Diseases Common to Man and Animals. Scientific Publication No. 254. PAHO, World Health Organization, Washington, D.C. EUA. 1989.
5.American Veterinary Medical Association: 2001. 2000 Report of the AVMA Panel on Euthanasia. Am. J. Vet. Med. Assoc. 218(5): 669-696.
6.Animal Advisory Committee c/o Ministry of Agriculture. Code of Animal Welfare No. 17: Code of Recommendations and Minimum Standards for the Care and Use of Animals for Scientific Purposes. Wellington, New Zealand. 1995.
7.Animal Welfare Institute. Washington. Comfortable Quarters for Laboratory Animals. Washington, D.C., EUA. 1979.
8.ASHP Technical Assistance Bulletin on Handling Cytotoxic and Hazardous Drugs. Am. J. Hosp. Pharm. 1990, 47: 1033-1049.
9.Aubert A. Sickness and Behaviour in animals: a motivational perspective. Neurosci. Behav. Rev. 1999, 23:1029-1036.
10. Autor Anónimo. Alternatives to Laboratory Animals. ATLA 1996, 24(4): 453-629.
11. Autor Anónimo. Sacrificio Humanitario de los Animales de Laboratorio. Animales de Experimentación 1996, 2(1):10-18.
12. Bateson P. Assessment of pain in animals. Anim. Behaviour 1991, 42: 827-839.
13. Cambridge, A.J., Tobías, K.M., Newberry, C.R., Sarkar, K.D. Subjetive and objective measurements of postoperative pain in cats. 2001, JAVMA, 217(5): 685-690.
14. Canadian Council on Animal Care (CCAC). Categories of Invasiveness in Animal Experiments. A Report, October. Ottawa, Ontario K1P 5H3, Canadá. 1989.
15. Canadian Council on Animal Care. Guidelines on: Choosing an appropriate endpoint in experiments using animals for research, teaching and testing. Ottawa. 1998.
16. Consejo Canadiense de Protección de los Animales. Manual Sobre el Cuidado y Uso de los Animales de Experimentación. Volumen 1. 2a. edición., Otawa, Ontario, Canadá, 1998.
17. Council of International Organizations of Medical Sciences (CIOMS). International Guiding Principles for Biomedical Research Involving Animals. CIOMS, Geneva. 1985.
18. De Grazia D. and Rowan A. Pain, suffering and anxiety in animals and humans. Theoretical Medicine 1991, 12:193-211.
19. Dolan Kevin. Ethics, Animal and Science. Brown, Iowa. 1999.
20. Federación de Asociaciones Europeas de las Ciencias del Animal de Laboratorio (FELASA). Recomendaciones de FELASA sobre los estudios y la formación de las personas que trabajan con animales de laboratorio: Categorías A y C. Laboratory Animals.
21.FELASA. Pain and distress in laboratory rodents and lagomorphs. 1994, Lab. Anim. 18: 97-112.
22. Flecknell, Paul. Manual of Rabbit Medicine and Surgery. British Small Animal Veterinary Association. London, U.K. 2000.
23. Foster, H.L., Small, J.D., Fox J.G. (eds.). The Mouse in Biomedical Research, Vols. I, II, III, IV. Academic Press, Inc. New York, EUA. 1983.
24. Harkness, J.E. and Wagner, J.E. Clinical Procedures. En: The Biology and Medicine of Rabbits and Rodents. Lea and Febiger eds. Philadelphia, PA., EUA. pp. 41- 45. 1977.
25. Hawk, T. and Leary, L. Steven. Formulary for Laboratory Animal. Academic Press. New York. 1999.
26. Hart, B.L. Biological basis of the behaviour of sick animals. Neurosci. Behav. Review 1998, 12: 123-137.
27. Hart, L.A. (editor) Responsible Conduct with Animals in Research. Oxford University Press, New York, 1998.
28. Institute of Laboratory Animal Resources (ILAR) Laboratory Animal Management: Nonhuman Primates. ILAR Committee on Nonhuman Primates, Subcommittee on Care and Use. ILAR News 1980, 23 (2-3): 1-44.

29. Kaplan, J.E. Guidelines for Prevention of Herpesvirus Simiae (B Virus) Infection in Monkey Handlers. The B Virus Working Group. J. Med. Primatol. 1988, 17: 77-83.
30. Manser, C.E. The Assement of Stress in Laboratory Animals. The Royal Society for the Prevention of Cruelty to Animals, London, U.K. 1992.
31. Mc Laughlin, R. Institutional Animal Care and Use Committee Review of Animal Care and Use Programs. Contemporary Topics Lab. Anim. Sci. 1993, 32(3): 12-16.
32. Mount, L.E. and Ingram, D.L. The Pig as a Laboratory Animal. Academic Press, London 1971.
33. National Institute of Health. Institutional Animal Care and Use Committee Guidebook. NIH publication 92-3415. Bethesda, MD, EUA. 1992.
34. National Institutes of Health/Office for the Protection of Research Risks (NIH/OPRR). Animal Care and Use Committee Policy Issues in the 1990's. Proceedings of NIH/OPRR Conference, Bethesda, MD, EUA. 1989.
35. National Research Council. Occupational Health and Safety in the Care and use of Research Animals. ILAR Journal 38(2): 89-93, 1997.
36. National Research Council. A Report of the Institute of Laboratory Animal Resources (ILAR). Committee on Laboratory Anmal Housing. National Academy of Sciences, Washington, D.C., EUA, 1987.
37. New York Academy of Sciences. Interdisciplinary Principles and Guidelines for the Use of Animals in Research, Testing and Education. New York Academy of Sciences, New York, EUA, 1988.
38. Nutrient Requirements of Laboratory Animals. 3rd Rev. ed. Board on Agriculture and Renewable Resources (BARR). Subcommittee on Laboratory Animal Nutrition, Committee on Animal Nutrition, Nutrient Requirements on Domestic Animals Series. National Academy of Sciences, Washington, D.C., EUA, 1978.
39. Olfert, E.D., Cross B.M. and McWilliam, A.A. (editors). Guide to the Care and Use of Experimental Animals. Vol. I., Canadian Council on Animal Care. Ottawa, Ontario KIP 5H3, Canadá. 1993.
40. Orlans, F.B. In the Name of Science: Issues in Responsible Animal Experimentation. Oxford University Press. New York, EUA. 1993.
41. Orlans, B., Simmonds, R.C. and Dodd, W.J. (editores). Effective Animal Care and Use Committees. Scientists Center for Animal Welfare, Bethesda, Md. 1987.
42. Poole, T. (editor). The UFAW handbook on the Care and Management of Laboratory Animals. Blackwell Science, Oxford, UK 1999.
43. Public Health Service (PHS). Policy on Humane Care and Use of Laboratory Animals, U.S. Department of Health and Human Services, Washington, D.C., EUA, 1986.
44. Schwindaman, D. Federal regulation of experimental animal use in the United States of America. Rev. Sci. Tech. Off int. Epiz. 1994, 13(1): 247-260.
45. Simmonds, R.C. Characteristics of Laboratory Animal Facilities. En: Handbook of Facilities Planning Vol. 2, Laboratory Animal Facilities. Editado por T. Ruys, Van Nostrand Reinhold, New York, EUA p. 1-33, 1991.
46. Small, J.D. Environmental and equipment monitoring. En: The Mouse in Biomedical Research, Vol. III, p. 87-89. Editado por H.L. Foster, et al. Academic Press, Inc. New York, EUA, 1983.
47.U.S. Department of Agriculture. Animal Welfare Regulations (U.S.D.A). Animal Welfare Act. Title 9 Code of Federal Regulations, Chapter I. Subchapter A - Animal Welfare. 1994.

15. Disposiciones transitorias

La presente Norma entrará en vigor al día siguiente de su publicación en el **Diario Oficial de la Federación**, excepto los siguientes puntos con las vigencias que a continuación se indican:

Con respecto a la certificación del Médico Veterinario Zootecnista y del personal certificado: 4.3.1.1.; 4.3.1.2.; 4.3.1.3.; inciso i) del 4.5.2.; 9.2.2.1; 9.2.2.4.: 5 años después de la entrada en vigor de esta Norma.

Con respecto al punto 4.4. de obtención de animales, primer párrafo: 5 años después de la entrada en vigor de esta Norma.

En relación con el punto 5.1.1. Cuadro 2, Espacio mínimo para roedores: 3 años después de la entrada en vigor de esta Norma.

En la Ciudad de México, Distrito Federal, a los nueve días del mes de agosto de dos mil uno.- La Coordinadora General Jurídica de la Secretaría de Agricultura, Ganadería, Desarrollo Rural, Pesca y Alimentación, **Lilia Isabel Ochoa Muñoz**.- Rúbrica.

Apéndice A (Normativo) Informe anual de actividades

I.- Tipo de bioterio:

A) Producción: Reproducción, Crianza, Manutención/Distribución.

B) Experimentación (uso): investigación científica, desarrollo tecnológico e innovación, pruebas de laboratorio y enseñanza.

C) Mixto: Incluye la combinación de los incisos A) y B).

II.- Destino de los animales de laboratorio:

A) Venta y distribución.

B) Uso en investigación científica, desarrollo tecnológico e innovación.

C) Uso para producción de biológicos y su calidad.

D) Uso con fines de diagnóstico.

E) Uso en enseñanza.

III.- Manifestación de inicio o continuación de actividades.

IV.- Nombre, dirección y teléfono del responsable administrativo y del Médico Veterinario responsable.

V.- Características físicas del bioterio.

VI.- Condición microbiológica de los animales alojados.

A) Convencionales.

B) Libres de patógenos específicos.

C) Gnotobióticos.

D) Axénicos.

E) Otro (especificar).

VII.- Nombre de las especies animales alojadas.

A) Número promedio de hembras adultas en producción al año por especie.

B) Número total de animales producidos al año por especie.

C) Número total de animales utilizados al año por especie.

D) Número total de proyectos registrados o atendidos al año por especie. En el caso de que un proyecto involucre el uso de varias especies, éstas se manifestarán por separado.

Apéndice B (Normativo) Requisitos mínimos que debe contener la propuesta de uso de los animales de laboratorio:

I. Título.

II. Descripción general.

III. Nombre, dirección, teléfono, fax y correo electrónico del responsable y del suplente designado.

IV. Adscripción.

V. Presentar la justificación de la elección de la especie, raza, línea o cepa de animales de laboratorio, así como del número de animales a utilizar.

VI. Indicar el destino de los animales una vez concluido el trabajo motivo de la propuesta.

VII. Indicar si los animales u órganos pueden tener uso para otra propuesta.

VIII. Indicar el método de eutanasia a emplear.

IX. Indicar, en su caso, el empleo de agentes patógenos o zoonóticos, sustancias radiactivas, cancerígenas u otras potencialmente dañinas.

Apéndice A (Informativo) Clasificación de actividades experimentales de acuerdo al grado de invasión, molestia o daño producido sobre los animales de laboratorio

CATEGORIA A. Experimentos utilizando invertebrados de baja escala: Uso de huevos, protozoarios u otros organismos unicelulares. Uso de metazoarios. Uso de cultivo de tejidos u órganos obtenidos después del sacrificio del animal en el rastro o necropsia.

CATEGORIA B. Experimentos que causan molestia o estrés mínimo: Restricción momentánea del animal con propósitos de observación clínica; toma de muestras de sangre e inyección de sustancias por las vías intravenosa, subcutánea, intramuscular, intraperitoneal u oral. Estudios agudos sin supervivencia del animal encontrándose éste completamente anestesiado. Uso de métodos de eutanasia con inconsciencia rápida del sujeto; por ejemplo, sobredosis de anestésicos. Periodos cortos de abstinencia de agua o alimento equivalentes a lo que pudiera darse en forma natural.

CATEGORIA C. Experimentos que causan estrés menor o dolor de corta duración: Canulación o cateterización de cavidades corporales o vasos sanguíneos mayores bajo anestesia. Procedimientos quirúrgicos menores como toma de biopsias bajo anestesia. Restricción física con objetivos más allá de la simple observación clínica pero sin llegar a producir estrés importante. Periodos cortos de abstinencia de agua o alimento pero que excedan lo observable en la naturaleza. Estos procedimientos no deben traducirse en cambios significativos de la apariencia física del animal, ni tampoco en la alteración de parámetros fisiológicos como la frecuencia cardiaca, frecuencia respiratoria, intensidad de la diuresis y defecación, o bien en su respuesta social o conductual; no debe haber anorexia, automutilación, hiperactividad, somnolencia excesiva, vocalización aumentada, comportamiento agresivo-defensivo o signos de autoaislamiento y retracción social.

CATEGORIA D. Experimentos que causan estrés o dolor moderado a severo: Procedimientos quirúrgicos mayores bajo anestesia general y con sobrevida del animal. Restricción física prolongada (hora-días). Inducción de estrés conductual tales como deprivación materna, agresión, interacciones predador-depredador. Procedimientos que causen alteraciones sensoriales y motoras severas, persistentes o irreversibles. El uso del Adyuvante Completo de Freund; o bien, cualquier procedimiento que, anatómica o fisiológicamente, resulte doloroso. Exposición a estímulos nocivos de los cuales no pueda escapar el animal. Producción de enfermedad por radiación. Administración de medicamentos o químicos que alteren la fisiología del animal.

CATEGORIA E. Procedimientos que causen dolor severo al nivel o por arriba del umbral de tolerancia del animal consciente no anestesiado: No solamente se consideran aquí procedimientos quirúrgicos sino exposición a medicamentos o agentes químicos o estímulos nocivos cuyos efectos son desconocidos. Esta exposición supone una marcada alteración de los sistemas fisiológicos pudiendo causar la muerte, dolor severo o estrés extremo. Se deben considerar en esta categoría cualquier experimento biomédico con alto grado de invasión, estudios conductuales de los que se conozca poco su respuesta y efectos al estrés, el uso de paralizantes musculares sin anestesia, la provocación de trauma o quemaduras en el animal no anestesiado, métodos no aprobados de eutanasia, pruebas de toxicidad e infección experimentalmente inducida que tengan como punto final la muerte del animal.

Apéndice "B" (Informativo)

Principales Zoonosis de Animales Experimentales al Hombre
ENFERMEDADES POR BACTERIAS

Enfermedad en el Hombre	Agente Causal	Huésped (animales vertebrados)	Forma de Diseminación	Vectores y notas sobre ciclo vital

Antrax	Bacillus antracis	Animales de granja, salvajes y de zoológico	Contacto, inhalación, ingestión	Esporas de larga duración en suelo
Brucelosis	B. suis<br>B. abortus<br>B. melitensis<br>B. ovis<br>B. canis	Porcinos<br>Ganado bovino y ovino<br>ovinos,<br>caprinos<br>ovinos perros	Contacto e ingestión de leche y productos lácteos y carne fresca	
Campilobacteriosis	C. fetus, C. jejuni	Conejos, puercos, perros, primates no humanos, aves	Ingestión	
Clamidiosis Psitacosis	Clamydia spp.	Pájaros, aves de corral	Inhalación	
Colibacilosis	E. coli	Ganado bovino, borregos aves	Ingestión	
Leptospirosis Enfermedad de Weil´s	Leptospira spp.	Roedores, perros animales de granja	Por contacto, orina contaminada	
Pasteurelosis	P. multocida<br>P. hemolítica<br>P. neumotrópica	Gatos, perros, conejos, otros mamíferos y pájaros	Contacto, por mordida y por inhalación	
Plaga	Yersinia pestis	Roedores	Contactos: mordida de pulga o por inhalación	Pulgas
Pseudotuberculosis	Yersinia pseudotuberculosis	Roedores, lagomorfos, palomas, pavos, canarios y pájaros	Contacto, por ingestión de comida y agua contaminada	
Fiebre por mordida de rata	S. moniliformis Spirilum minus	Roedores	Mordida de roedor	Saliva infectada
Salmonelosis	Salmonella spp.	Animales de granja, roedores y reptiles, anfibios y animales de zoológico	Ingestión, inhalación y contacto	

Shigelosis	Shigella spp.	Primates no humanos	Contacto materia fecal, ingestión	Directo por fomites
Tétanos	Cl. tetani	Perros, gatos, caballos	Por mordida, por punciones contaminadas	Tierra
Tuberculosis	M. tuberculosis<br>M. bovis.<br>M. avium	Primates no humanos, ganado vacuno, perros, ganado vacuno, perros, aves, porcinos, ovejas	Contacto, ingestión e inhalación	Antropozoonótica
Tularemia<br>Fiebre del conejo	F. tularensis	Lagomorfos, roedores silvestres, pájaros y perros	Inhalación, contacto, piquetes de insectos por comida y agua contaminada	Picadura de insecto y garrapata

ENFERMEDADES POR RICKETTSIA

Enfermedad en el Hombre	Agente Causal	Huésped (animales vertebrados)	Forma de Diseminación
Fiebre Q	Coxiella	Ganado vacuno ovino y caprino	Inhalación, ingestión de leche contaminada, sangre chupada por artrópodos, por contacto con líquido amniótico de la placenta
Viruela rickettsial	R. akari	Ratones salvajes y ratas	Mordidas de ácaros A. sanguineus
Fiebre manchada (de las montañas Rocallosas)	R. rickettsia	Roedores salvajes, conejos y perros	Mordida de garrapata
Tifo siberiano (fiebre de la garrapata asiática)	R. sibérica	Varios roedores salvajes	Mordida de garrapata
Tifo murino	R. typhy	Ratón salvaje y ratas	Mordida de pulga y alimentos contaminados

ENFERMEDADES POR ARBOVIRUS

Enfermedad en el Hombre	Agente Causal	Huésped (animales vertebrados)	Forma de Diseminación	Vectores
Fiebre hemorrágica	Arbovirosis asiática	Roedores salvajes. Liebres monos salvajes	Mordida de garrapata en clima subtropical se favorece el ciclo	

Encefalitis de California	Encefalitis de California	Conejos salvajes y roedores	Ciclo natural en roedores salvajes y conejos/mosquito	
Fiebre colorado	Virus por piquete de garrapata	Ardilla, Deromyscus spp.	Mordida de garrapata	Ciclo natural en roedores
Encefalitis equina del este	EEE	Caballos y pájaros	Picadura de mosco	
Encefalitis Ponwassan	Virus Ponwassan	Conejos salvajes y roedores	Mordedura de garrapata	
Encefalitis de San Luis	ESL	Aves	Ciclo natural en aves	
Encefalitis equina venezolana	Encefalitis equina venezolana	Caballos	Ciclo natural en caballos	
Encefalitis equina del oeste	Encefalitis equina del oeste	Caballos, pájaros	Piquete de mosco	Ciclo natural ave/mosquito /caballo

ENFERMEDADES POR OTROS VIRUS

Enfermedad en el Hombre	Agente Causal	Huésped (animales vertebrados)	Forma de Diseminación
Enfermedad de Marburg, Fiebre hemorrágica de Ebola	Filovirus	Mono verde del Africa<br>Macaca sp.	Contacto directo de tejidos de mono
Fiebre hemorrágica suramericana y coreana	Virus de fiebre hemorrágica	Roedores Salvajes<br>Mastomys ratelensis	Contacto: contaminación de alimentos con excreta de roedores
Hepatitis A	Virus de la hepatitis	Chimpancé	Contacto; enfermedad antropozoonótica
Encefalitis por herpes B	Herpes simae	Rhesus; otros Macaca	Contacto, heridas por mordedura, monos del viejo mundo
Linfocoriomeningitis	Virus LCM	Roedores, otros mamíferos	Contacto, inhalación, transmisión congénita, transmisión por cultivo de tejido
Rabia	Virus de la rabia	Perros, gatos, murciélagos, otros	Mordeduras, contacto con saliva,<br>el virus se concentra en la saliva

ENFERMEDADES POR HONGOS Y PROTOZOARIOS

Enfermedad en el Hombre	Agente Causal	Huésped (Animales vertebrados)	Forma de Diseminación
Balantidiasis	Balantidium coli	Primates no humanos	Ingestión de alimento contaminado, fomites
Coccidioidomicosis	Coccidioides imitis	Ganado, perros	Inhalación de esporas aéreas, hongo presente en tierra del desierto
Amibiasis, disentería amibiana	Entamoeba histolytica	Primates no humanos, perros	Contaminación de alimento, usualmente del hombre (huésped natural) hacia el perro
Giardiasis	Giardia intestinalis	Primates no humanos, perros	El hombre es el principal reservorio, ingestión de quistes en agua o alimentos contaminados
Histoplasmosis	Histoplasma capsulatum	Perros y otras especies domésticas y salvajes	Inhalación del hongo, puede crecer en el suelo
Toxoplasmosis	Toxoplasma gondii	Gatos	Ingestión de ovoquistes del gato, inhalación de carne infectada, puede ocurrir transmisión fetal
Dermatomicosis, ringworm	Trichophyton sp, Microsporum sp, otros dermatofitos	Perro, gato, cobayo, otros roedores, conejos	Contacto directo, ringworm puede transmitirse de hombre a animal y viceversa, suelo puede ser reservorio
Enfermedades sanguíneas por protozoarios	Trypanosoma sp, Plasmodium sp, Leishmania sp	Primates no humanos, roedores	Transmisión saliva-insecto vector; algunas especies transmisión directa

Apéndice C (Informativo)

Indicadores que deben ser evaluados en animales bajo el efecto de anestésicos

I. Patrón respiratorio: Incluye no únicamente al número de respiraciones por minuto, sino también su profundidad y las características de la respiración del animal.

II. Color de las mucosas: Si un animal está recibiendo suficiente oxígeno durante la anestesia sus membranas mucosas permanecen rosadas. Esto puede evaluarse en la mayor parte de los animales observando las mucosas bucal, rectal o vulvar.

III. Tiempo de llenado capilar: Este es después de su liberación de una presión física que los mantuvo vacíos. Un llenado capilar para la mayor parte de los mamíferos es de menos de dos segundos.

IV. Pulso: El pulso debe tomarse para evaluar la fuerza y las características del mismo, como un medio para valorar la respuesta del animal a su actividad cardiovascular.

V. Presión sanguínea: La valoración de la continuidad de la presión sanguínea puede ser monitoreada o valorada por medio de detectores de flujo ultrasónico Doppler.

VI. Temperatura corporal: La anestesia quirúrgica paraliza los centros regulatorios de calor del cerebro, lo que resulta en una caída de la temperatura del paciente. Entre más baje la temperatura es más baja la capacidad del paciente para metabolizar las drogas.

VII. Ojos: Los ojos de un animal anestesiado proporcionan una ventana del estado de anestesia.
VIII. Reflejos: Los reflejos que más frecuentemente se evalúan son el podal y el anal. Estos dos reflejos están presentes durante los planos quirúrgicos tres y cuatro, respectivamente, pero desaparecen cuando se alcanza la etapa cuatro de anestesia.
